# Supplementary material for: Trustworthy Stress Detection from Wearable Sensor Signals for Preventive Anxiety Monitoring: Subject-Independent Accuracy and Calibrated Confidence with the Autonomic-Branch Quantum-Graph Network
Source: Sensors (Basel). 2026 Oct 11;26(20):6425. doi: 10.3390/s26206425 (PMC13709010; doi:10.3390/s26206425)
Supplement: Supplementary file 1 [file sensors-26-06425-s001.zip › sensors-4523090-supplementary/Definitions/sensors-logo-eps-converted-to.pdf]

sensors
