# Supplementary material for: Trustworthy Stress Detection from Wearable Sensor Signals for Preventive Anxiety Monitoring: Subject-Independent Accuracy and Calibrated Confidence with the Autonomic-Branch Quantum-Graph Network
Source: Sensors (Basel). 2026 Oct 11;26(20):6425. doi: 10.3390/s26206425 (PMC13709010; doi:10.3390/s26206425)
Supplement: Supplementary file 1 [file sensors-26-06425-s001.zip › sensors-4523090-supplementary/MDPISensors_main_proof_TrustworthyStressDetectionfromWearableSensorSignalsforPreventiveAnxietyMonitoring.pdf]

Definitions/sensors-logo-eps-converted-to.pdf

MDPI

Article

Trustworthy Stress Detection from Wearable Sensor Signals for Preventive Anxiety Monitoring: Subject-Independent Accuracy and Calibrated Confidence with the Autonomic-Branch Quantum-Graph Network

Spyridon Papamentzelopoulos and Sotirios Nikoletseas *

Computer Engineering and Informatics Department, University of Patras, 26500 Patras, Greece; papamentze@ceid.upatras.gr
* Correspondence: nikole@cti.gr

Highlights

What are the main findings?

- One fixed architecture and feature pipeline inflate by 27–61 macro-F1 points on WESAD and SWELL-KW when subjects or adjacent windows are allowed to straddle the train–test split, and by up to 67 points on a third corpus, AffectiveROAD, on which honest performance is at chance.
- The same leakage corrupts confidence—conventional calibration practice leaves gradient-boosting at 25.6% expected calibration error against 5.4% after subject-disjoint refitting; with temperature scaling, the fitted temperature sits at the grid floor of 0.05 on a subject-sharing split and at 6.0 on a subject-disjoint one.

What are the implications of the main findings?

- A wearable stress monitor cannot be trusted on an unseen wearer unless both the evaluation split and the calibration split are subject-disjoint; reporting the difference costs one extra run.
- Encoding named autonomic branches on fewer than six qubits gives an inspectable model whose two-qubit correlators are not linearly redundant and survive finite-shot sampling and simulated depolarizing noise.

Abstract

Wearable monitoring of autonomic dysregulation preceding anxiety disorders must generalize to unseen wearers; reported accuracy reflects the evaluation protocol as much as the model. We measured it with one fixed instrument, the Autonomic-Branch Quantum-Graph Network (AB-QGN), mapping each autonomic branch onto one named qubit of a shallow entangling circuit. With architecture and features fixed, heart-rate variability (HRV) and electrodermal (EDA) features from WESAD and SWELL-KW went through leakage-free subject-grouped and leave-one-subject-out (LOSO) protocols and two leaky ones: single-modality configurations inflated by 27–61 macro-F1 points. Honest fused macro-F1 was 86.2 (WESAD) and 61.4 (SWELL-KW), 84.0 and 55.1 under LOSO; that distance from near-ceiling figures is the result, not a shortfall. Leakage corrupted confidence: conventional calibration left a gradient-boosting baseline at 25.6% expected calibration error, 5.4% after subject-disjoint refitting. Over structure- and parameter-matched classical twins, the quantum core added 3–12 session-level points on SWELL-KW at every seed, not significant at 51 sessions, and none on WESAD when parameter-matched. On a third corpus, AffectiveROAD, honest performance is at chance per window, while window-overlap leakage lifts tree ensembles to 90–98 macro-F1. We claim no advantage over fixed-configuration tree ensembles: the contribution is an auditable encoder and the protocol needed to trust a number.

Check for updates

Academic Editor: Salvatore Andrea Pullano

Received: 9 August 2026
Revised: 17 September 2026
Accepted: 8 October 2026
Published:

Copyright: © 2026 by the authors. Licensee MDPI, Basel, Switzerland. This article is an open access article distributed under the terms and conditions of the Creative Commons Attribution (CC BY) license.

Keywords: wearable stress detection; heart-rate variability; electrodermal activity; sensor data fusion; data leakage; leave-one-subject-out cross-validation; model calibration; quantum machine learning; hybrid quantum–classical neural network; physiological sensing

1. Introduction

Chronic stress dysregulation and anxiety disorders activate the same stress axis and frequently follow one another [1], and objective physiological markers of affective state remain a clinical priority [2]. The clinical interest in wearables is preventive. Continuous monitoring could flag sustained dysregulation early enough to act on in people who are not yet patients. Wearables expose the two most accessible autonomic channels at a quality sufficient for affect inference [3]: heart-rate variability (HRV), reflecting sympathetic–parasympathetic interaction, and electrodermal activity (EDA), indexing sympathetic sudomotor drive [4–6]. Such a monitor earns its place only if it holds up for someone it has never seen and only if the confidence attached to its output can be acted on.

A mature literature maps these signals to affective labels. Hand-crafted features feed tree ensembles and support-vector machines, or end-to-end convolutional and recurrent models [6,7] of the kind established on the adjacent electroencephalographic problem [8], with multimodal fusion consistently outperforming any single channel [7,9]. Two ceilings recur. Models developed and tested on the same individuals generalize poorly to unseen people [10], because physiological baselines differ markedly between subjects [11]; and much of the field's headline accuracy is a property of the evaluation rather than the model [12].

Subject-dependent splits and overlapping sliding windows (a common construction in time-series pipelines [13]) leak information from training to test partitions and inflate reported accuracy [12,14]. The clearest demonstration sits within a single study. In a stratified split described as subject-independent, a quantum transfer-learning model achieved 99.99% accuracy on WESAD. When tested in a leave-one-subject-out manner on the same data, it yielded 52.1% three-class accuracy [15]. A systematic review of physiological anxiety detection arrives at the same conclusion from the other direction: subject-independent evaluation is underused, and reported metrics likely overstate real-world efficacy [16].

Simultaneously, quantum machine learning (QML) proposes kernel methods and parameterized circuits whose feature maps exist within exponentially large Hilbert spaces [17–19]. With data re-uploading, few-qubit circuits are universal approximators [20]. That matters, because deep circuits initialized randomly suffer from barren plateaus—gradients vanishing exponentially in qubit count [21]—on the noisy intermediate-scale hardware now available [22]. QML has been demonstrated on medical images and tabular records [23–25] and recently on stress signals [26–29]. However, existing quantum stress models are evaluated inconsistently, impose no structural inductive bias on the physiology, and report no calibration.

This paper measures both requirements and finds them to be the same problem twice. We fix one architecture and feature pipeline and drive them through three protocols differing in how subjects and adjacent windows are partitioned; a fixed instrument licenses the attribution, since any difference between protocols is then a property of the protocol. Applying the same discipline to calibration exposes a second, unreported face. Post hoc calibrators are themselves fitted on a held-out split, and when that split shares subjects with training, the calibrator is validated by the leakage it should guard against, so confidence is corrupted invisibly. That matters because a preventive monitor emits not a label but a probability, which a threshold turns into an alert or a referral. The instrumented architecture is the Autonomic-Branch Quantum-Graph Network (AB-QGN), with each qubit encoding an interpretable autonomic branch, branches entangled along a physiological graph through trainable two-qubit couplings.

Our contributions are fivefold. First, a controlled benchmark quantifying accuracy inflation on a fixed architecture across two datasets and three protocols, and a portability check of the same measurement on a third corpus, AffectiveROAD, with a feature front end of our own (Section 4.11). Second, the identification and measurement of calibration leakage, which moves expected calibration error up to six-fold (Section 4.3). Third, to our knowledge, the first calibration analysis in this domain, set against strong classical baselines and a structure-matched twin whose capacity is reported exactly. Fourth, evidence that the encoder's correlator readout is not linearly recoverable from single-qubit expectations, is load-bearing under ablation, and survives finite-shot sampling and simulated device noise. Fifth, an account of what the quantum core adds over classical encoders matched in structure and in capacity, tested rather than asserted, and reported as non-significant where it is (Section 4.6). Section 2 positions the work, Sections 3 and 4 give methodology and results, and Sections 5 and 6 the implications and conclusions.

2. Related Work

2.1. Wearable Sensing, the Cross-Subject Gap, and Evaluation Integrity

Ishaque et al. [5] catalog the HRV feature families—RMSSD, pNN50, LF/HF, SD1/SD2, and sample entropy—that AB-QGN organizes into autonomic super-nodes, while EDA decomposes into slow tonic and fast phasic components [7]. Two benchmarks dominate. WESAD is a multimodal dataset over fifteen subjects with baselines reaching at most 80% three-class accuracy [30]; SWELL-KW integrates physiological and behavioral sensing from knowledge workers under time-pressure and interruption stressors [31,32].

The recurring failure mode is cross-subject generalization. LOSO accuracy is much lower than random-split accuracy on the same wearable data [10], and Vos et al. in a systematic review conclude that generalizable performance is far lower than what is typically reported and identify inconsistent validation as a reason for poor reproducibility [11]. There are two remedies. The first is personalization [33] and the second is domain-invariant representation learning [34]. The latter is implemented in AB-QGN by a gradient-reversal subject adversary [35].

The mechanism is documented: Hughes and Zhang observe a model reported above 98% three-class accuracy collapse once data were partitioned by subject [12], and the same holds for LSTM forecasting [14]. The SWELL-KW authors state the window-overlap channel directly—consecutive one-minute rows are near-duplicates, so a random split seats very similar cases on either side of it—and they measure its cost on their own data. Accuracy of 90.0% under ten-fold cross-validation falls to 58.9% on an unseen participant, below their 61.8% majority-class baseline [32]. Anxiety-detection models prove fragile under perturbation [36]. Calibration is separately under-reported—modern networks are systematically overconfident [37], adaptive temperature variants improve robustness [38], and addressing imbalance and confidence jointly benefits both [39]—and no prior work in this domain examines whether the calibrator's own split leaks. We report ECE, NLL, and Brier throughout.

2.2. Quantum Machine Learning: Primitives and Biosignal Applications

The primitives that AB-QGN is based on are well established. Classically hard kernels are defined by quantum support-vector machines and quantum-enhanced feature spaces [17,18]. Variational versus quantum-kernel classifiers are characterized by comparative studies [40], with hybrid quantum convolutional variants extending the variational route to the multiclass setting [41]. Our design is guided by two results. Data re-uploading turns even a single-qubit classifier into a universal approximator, so expressivity does not need register width [20], and it extends the Fourier spectrum we can access, so depth is the lever [42]. Conversely, gradients in random parameterized circuits vanish exponentially with the number of qubits [21] under NISQ constraints [22]. These results jointly motivate a small, structured, shallow ansatz.

https://doi.org/10.3390/s1010000

Medical QML has focused on images and tabular records [23–25]. Applications to stress signals are recent, few, and inconsistently evaluated. Reported figures include 0.80 accuracy on SWELL-KW under an unspecified split [26], anomaly detection with a hybrid quantum SVM [27], and F1 0.9999 on WESAD-derived HRV under a split with no subject-level partitioning reported [28]. On a held-out test split, a quantum-kernel SVM and a tree-tensor-network QNN reached only 45% and 55%, although that split is not reported as subject-disjoint [29]. The closest graph-structured antecedents are classical [43], the graph-neural-network literature on the adjacent electroencephalographic problem being surveyed separately [44]. In earlier work, we applied quantum classifiers to wearable physiological data for anxiety prevention [45]. That study optimized classifier variants on a different corpus and asked how far noise degrades them; the present work holds one architecture fixed across protocols and asks what evaluation and calibration protocols are required for any reported number to be meaningful. The encoder, the corpora, and the question all differ. We are not aware of prior work combining an interpretable graph-structured quantum encoder over autonomic branches with a controlled leakage-free benchmark and calibration reporting; Supplementary Tables S1 and S2 compare architectures in detail.

3. Materials and Methodology

3.1. Overview, Datasets and Features

AB-QGN performs three-class affect classification from wearable physiological features under the three controlled protocols in Section 3.5. The pipeline is summarized in Figure 1.

The pre-computed HRV and EDA feature windows from WESAD (15 subjects) and SWELL-KW, published on Kaggle by Nkurikiyeyezu [46] and based on Schmidt et al. [30] and Koldijk et al. [31,32] with the method of [47] are used. The release contains 22 SWELL-KW subjects from the original 25; all of them have been kept, but electrodermal features exist for only 21 of them. Each window is a tuple (x_i, y_i, s_i) with label $y_i \in \{0, 1, 2\}$ and subject identifier s_i; $D_{\mathrm{HRV}} = 34$, $D_{\mathrm{EDA}} = 22$ on SWELL-KW, and 32 and 14 on WESAD, whose release lacks the two nonlinear HRV indices (sample entropy and Higuchi fractal dimension) and the eight SCR peak and onset descriptors, which is why WESAD EDA realizes four autonomic branches rather than five (Section 3.3.4) and why the mRMR filter with $K = 24$ performs no selection there, all fourteen features passing. The tables contain 135,650 HRV and 33,948 EDA windows for WESAD and 391,638 and 98,486 for SWELL-KW. The class proportions are 52.8/17.0/30.2% and, on SWELL-KW, 54.2/17.4/28.3% (HRV) and 51.1/17.5/31.4% (EDA).

The two source protocols differ in population, setting and stressor, which is what makes the pair informative (Table 1). WESAD recruited seventeen participants, graduate students at the recording research center, and retained fifteen after two sensor malfunctions; recruitment excluded pregnancy, heavy smoking, mental disorders, and chronic or cardio-vascular disease, and the three labeled conditions supply roughly thirty-six minutes of data per participant; twelve were male and three female, with a mean age of 27.5 ± 2.4 years [30]. Each took part in a single laboratory session of roughly two hours that moved through a neutral baseline (about twenty minutes of reading), an amusement condition (a set of funny video clips, about six and a half minutes), a stress condition (the Trier Social Stress Test: a public speech on personal traits followed by mental arithmetic in front of a panel, about ten minutes), and two guided meditation periods, in one of two counterbalanced orders [30]. The three affective classes we use are baseline, amusement and stress; the meditation periods carry no class label in the feature release [46]. SWELL-KW recruited twenty-five participants, students and young knowledge workers (interns at TNO and students of Delft University of Technology), seventeen male and eight female, with a mean age of 25 ± 3.25 years [31]. Each performed typical knowledge work at an office

workstation, like writing reports, preparing presentations, reading e-mail and searching for information, piling them up in three blocks of about thirty to forty-five minutes: a neutral block, a time-pressure block in which the available time was cut to two-thirds, and an interruption block in which eight e-mails arrived during the work, some relevant to the task and some not [31,32]. The three classes are therefore no-stress, time-pressure and interruption. Neither release carries age or sex per subject code, so the cohort figures above are those of the source publications, and no subject-level stratification is possible (Section 5.3).

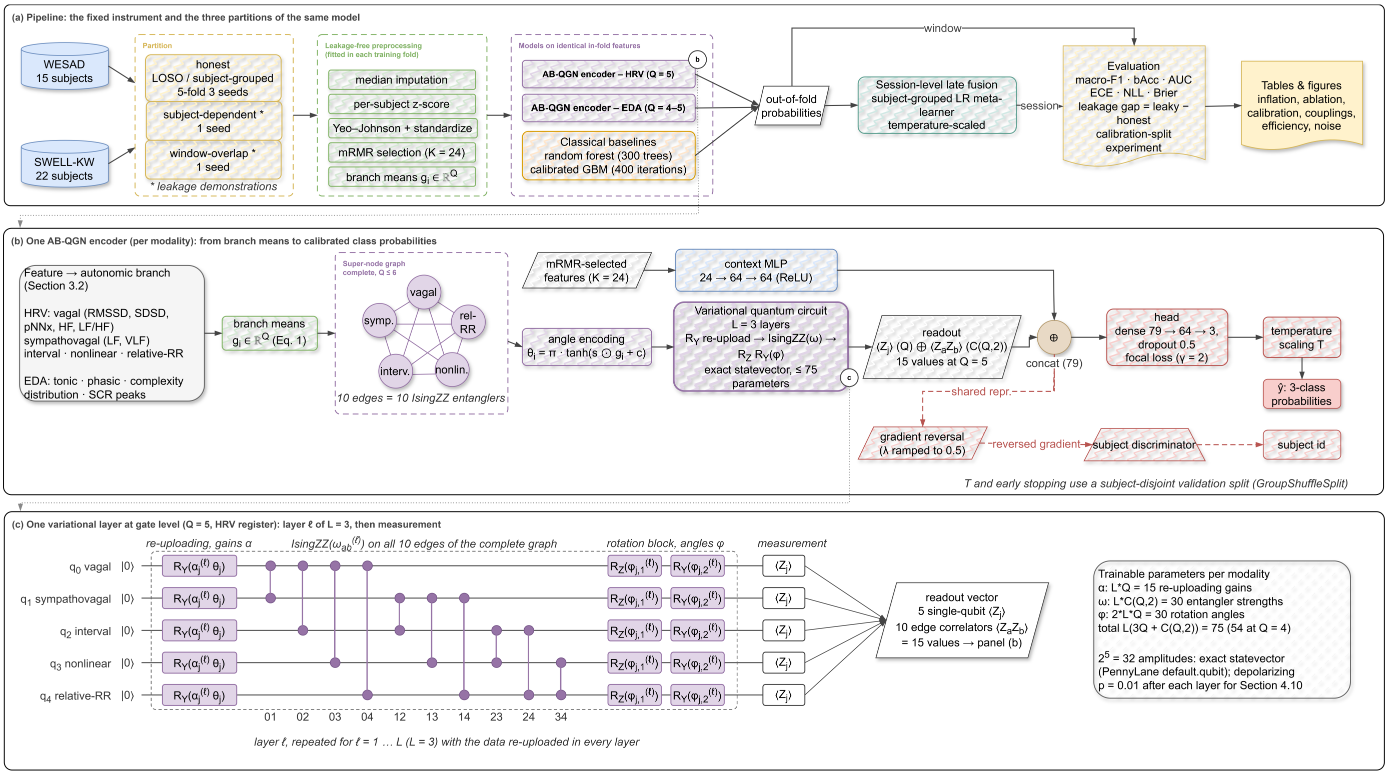

Figure 1. AB-QGN architecture at three levels. (**a**) Pipeline: the two feature corpora enter leakage-free preprocessing fitted inside each training fold (median imputation, Yeo–Johnson and standardization, mRMR selection with $K = 24$), together with per-subject z-scoring, which uses each subject's own windows and therefore also applies to the held-out subject (Section 3.5); branch means form the super-node signals from the full standardized matrix, while the mRMR subset feeds only the classical context branch. The corpora feed one AB-QGN encoder per modality alongside the classical baselines on identical in-fold features, are fused at session level by a subject-grouped logistic-regression meta-learner, and are evaluated under one honest and two leakage-inducing partitions of the same model. (**b**) One encoder: features are assigned to autonomic branches whose means form the super-node signals; a complete inter-branch graph fixes the entangler edges; angle encoding $\theta_i = \pi \tanh(s \odot g_i + c)$ drives an $L = 3$ variational circuit whose readout of single-qubit expectations and edge correlators ($Q + \binom{Q}{2}$ values) is concatenated with a classical context MLP and passed to a temperature-scaled softmax head; a gradient-reversal subject discriminator on the shared representation enforces subject invariance. (**c**) One variational layer at gate level for $Q = 5$: R_Y re-uploading rotations with trainable gains, IsingZZ entanglers on all ten graph edges, a trainable R_Z–R_Y rotation block, repeated L times before measurement of $\langle Z_j \rangle$ and $\langle Z_a Z_b \rangle$ in the computational basis. In panel (**a**) the asterisk marks the two deliberate leakage demonstrations, and the circled letters **b** and **c** mark the blocks expanded in the panels below. Abbreviations used in the figure: HRV, heart-rate variability; EDA, electrodermal activity; LOSO, leave-one-subject-out; mRMR, minimum redundancy–maximum relevance; RF, random forest; GBM, histogram gradient-boosting machine; bAcc, balanced accuracy; SCR, skin conductance response; LR, logistic regression; MLP, multilayer perceptron; AUC, area under the receiver operating characteristic curve; ECE, expected calibration error; NLL, negative log-likelihood.

Table 1. Source cohorts and acquisition chains of the two corpora, as documented by their authors [30–32] and by the feature release [46,47]. Demographics are cohort-level; the released tables index windows by an arbitrary subject code only.

	WESAD	SWELL-KW
Participants (recruited/retained in source)	17/15	25/25
Subjects in the feature release (HRV/EDA)	15/15	22/21
Sex (source cohort)	12 male, 3 female	17 male, 8 female
Age, years (mean ± SD)	27.5 ± 2.4	25 ± 3.25
Population	graduate students at the recording center	students and young knowledge workers
Setting	laboratory, seated and standing, single session (≈2 h)	laboratory office workstation, single session (≈3 h)
Stressors	Trier Social Stress Test (speech + mental arithmetic); amusement clips as a second affective state	time pressure (time cut to two thirds); e-mail interruptions
Classes used	baseline/amusement/stress	no-stress/time-pressure/interruption
Cardiac signal	chest ECG, RespiBAN Professional, 700 Hz	chest ECG, TMSi Mobi, 2048 Hz
Electrodermal signal	skin conductance; the release does not state which of WESAD's two channels (chest RespiBAN 700 Hz, wrist E4 4 Hz) it used	finger skin conductance, TMSi Mobi, 2048 Hz
Windows (HRV/EDA)	135,650/33,948	391,638/98,486
Fused sessions (subject × condition)	45	51
Per-subject demographics in the release	no	no

These two corpora were chosen because a fixed-instrument measurement of evaluation protocol needs, from each corpus, pre-computed windowed features of both modalities with subject identifiers and a three-class affective protocol, openly accessible without a signed end-user agreement, and released by a group independent of ours, so that the pipeline adds no signal-level processing of its own. To our knowledge the release used here is the only public one that satisfies all of these at once; Supplementary Section S12 lists the candidate corpora we examined: AffectiveROAD [48], the MIT driver-stress database [49], the non-EEG neurological-status set [50], the nurse-stress corpus [51], UBFC-Phys [52], StressID [53], CLAS [54], MAUS [55], Stress-Predict [56], the wearable exam-stress set [57], and the affect corpora AMIGOS [58], DEAP [59], DREAMER [60] and CASE [61] and the criterion each fails. Section 4.11 reports a portability check of the protocol finding on one of them, AffectiveROAD, with a front end of our own.

Both corpora precede this work, so the acquisition chain is theirs. Ethical governance is equally well documented. The original investigators collected both corpora with participant consent. WESAD was recorded under the approval of the workers' council and the data security officer of the recording research center [30]; SWELL-KW used a signed consent procedure with a post-experiment debriefing [31]. The SWELL-KW release is anonymized: its authors withheld the raw facial video and Kinect recordings to protect participant anonymity [31]. We used only these public distributions. No identifiable data passed through our pipeline, and we generated none. We restate it because the sensing front end bounds what any downstream feature can carry. WESAD instrumented 15 participants

https://doi.org/10.3390/s1010000

with two devices at once. A RespiBAN professional chest belt carried electrocardiography, respiration, electromyography, skin temperature, and three-axis accelerometry; an Empatica E4 wrist band carried blood volume pulse, electrodermal activity, skin temperature, and accelerometry [30]. SWELL-KW instrumented knowledge workers at a computer workstation under time pressure and email interruption, recording electrocardiography and skin conductance alongside behavioral and postural channels [31,32]. Our heart-rate-variability features therefore descend from an electrocardiographic inter-beat-interval series, and our electrodermal features from a skin-conductance channel. WESAD carries two electrodermal channels, the chest belt at 700 Hz and the wrist band at 4 Hz, and the feature release does not state which of them it used. The distinction matters. Chest electrocardiography resolves individual beats more reliably than wrist photoplethysmography, and every HRV index we use is a statistic of the interval series rather than of the raw waveform, so beat-detection error propagates into the feature table before we ever see it. The WESAD chest belt samples every channel at 700 Hz; the wristband, worn on the non-dominant hand, samples blood volume pulse at 64 Hz, electrodermal activity and temperature at 4 Hz, and acceleration at 32 Hz [30]. SWELL-KW recorded electrocardiography and skin conductance on a single TMSi Mobi body-sensor unit at 2048 Hz, the electrocardiographic leads self-adhesive and arranged in a triangular chest configuration, and the skin-conductance electrodes placed on the fingers [31,32]. We take the released tables as given and add no signal-level processing of our own. The leakage and calibration effects reported below are properties of how those windows are partitioned, not of the front end that produced them.

The HRV indices are computed from a moving window of five minutes on the inter-beat-interval series, advanced one interval at a time, and the electrodermal features from a moving window of ten minutes [47]; the release holds about four HRV rows per EDA row, and consecutive rows overlap by more than 99%. Every honest partition is subject-disjoint, which means that overlapping windows from one subject cannot straddle it. The overlap-leak protocol specifically inserts near-duplication. The two modalities are modeled separately and combined only at the decision level. This overlap is a property of the released tables, and not a choice made by us, and is exactly what the window-overlap protocol described below exploits. A feature table generated by sliding a five-minute window one interval at a time cannot be randomly partitioned without leaking.

3.2. Autonomic Super-Node Encoding

Each feature is assigned to an autonomic regulatory branch $b \in \mathcal{B}_m$ by a physiologically motivated rule. For HRV the branches are vagal (RMSSD, SDSD, pNNx, HF power and its normalized forms, and the LF/HF, HF/LF, and SDRR/RMSSD ratios, which the assignment rule places here), sympathovagal (LF and VLF power and their normalized forms), interval, nonlinear, and relative-RR; for EDA, tonic, phasic, complexity, distribution, and SCR peaks. The branch names organize the feature set by physiological origin and spectral scale; they are not mechanistic claims. Two caveats justify that restraint. Reading LF power and the LF/HF ratio as an index of sympathovagal balance is contested [62], and very-low-frequency power estimated from a five-minute window sits below the record length the measurement standard requires for a stable estimate [63]. We keep the grouping because it partitions the features consistently and makes each qubit nameable, and we attach no sympathetic readout to the branch so labeled. Branches are held in physiological priority order and truncated at six qubits, a feature past the cut joining the last retained branch so

that none are discarded. From the per-fold standardized matrix $\tilde{X}$, branch b's super-node signal is the mean of its constituent features,

$$g_{i,b} = \frac{1}{|F_b|} \sum_{f \in F_b} \tilde{x}_{i,f}, \qquad F_b = \{f : \beta_m(f) = b\}. \tag{1}$$

Equation (1) yields a compact, semantically labeled vector $g_i \in \mathbb{R}^Q$. Encoding all 34 HRV features directly would lead to an unfeasibly wide circuit, firmly in the barren-plateau regime [21]. Width is controlled by physiologically motivated coarse-graining, which preserves autonomic interpretation and the discriminative content of each regulatory system.

3.3. The Quantum Graph Core

3.3.1. Angle Encoding

The Q super-node signals are mapped to bounded, trainable rotation angles

$$\theta_i = \pi \cdot \tanh(s \odot g_i + c), \qquad s, c \in \mathbb{R}^Q, \tag{2}$$

where $\odot$ is the elementwise product and s, c are learned per-node scale and bias. The hyperbolic tangent confines angles to $(-\pi, \pi)$. That removes the wraparound ambiguity of an unbounded feature driving a rotation gate, while letting the network set each branch's dynamic range.

3.3.2. Variational Circuit

The angles of Equation (2) drive an L-layer ($L = 3$) circuit on the complete inter-branch graph with edge set $\mathcal{E}_q$; each layer applies data re-uploading, graph entanglers, and a trainable single-qubit rotation block,

$$U_\ell(\theta_i) = \underbrace{\prod_j R_Y(\phi_{j,2}^{(\ell)}) R_Z(\phi_{j,1}^{(\ell)})}_{\text{rotation}} \underbrace{\prod_{(a,b) \in \mathcal{E}_q} R_{ZZ}(\omega_{ab}^{(\ell)})}_{\text{entanglers}} \underbrace{\prod_j R_Y(\alpha_j^{(\ell)} \theta_{i,j})}_{\text{re-uploading}}. \tag{3}$$

In Equation (3), $R_{ZZ}(\omega) = \exp\big(-i\frac{\omega}{2} Z_a \otimes Z_b\big)$, and the layer is applied to $|0\rangle^{\otimes Q}$. Training updates the re-uploading gains α, the entangler strengths ω, and the rotation angles ϕ. Because re-uploading at every layer extends the accessible Fourier spectrum, depth rather than width supplies expressivity [20,42] (derivation in Supplementary Section S3). Each R_{ZZ} couples two super-nodes through a single scalar whose magnitude measures, sign-free, how strongly two branches must interact to determine the label. The circuit is, in that sense, a physiologically structured analog of graph message passing. For interpretation we summarize each edge by its mean trained magnitude across layers, $\kappa_{ab} = L^{-1} \sum_\ell |\omega_{ab}^{(\ell)}|$.

3.3.3. Readout

The branch representation is the vector of single-qubit Pauli-Z expectations augmented with the two-qubit edge correlators,

$$q_i = \Big[\langle Z_j \rangle\Big]_{j=1}^{Q} \,\Big\|\, \Big[\langle Z_a Z_b \rangle\Big]_{(a,b) \in \mathcal{E}_q}. \tag{4}$$

Equation (4) gives a readout dimension $Q + \binom{Q}{2}$. The single-qubit terms summarize each branch's marginal polarization; the correlators capture the joint behavior of branch pairs that no linear function of those marginals reproduces (Section 4.7 tests this).

3.3.4. Circuit Size and Trainability

The register size is determined by the number of branches occupied by the feature set of the modality; not more than six. The layout is fixed per dataset and modality, not per fold. The super-node signals are formed from the full in-fold standardized matrix, while the mRMR selection of Section 3.5 feeds only the classical context branch. A budget above the number of occupied branches adds no wires: the register is built from the occupied branches only, so larger budgets realize the identical circuit, the mechanism behind the saturation of Section 4.8. The layouts we use have $Q = 5$ for HRV on both datasets and for SWELL EDA (15-dimensional readout) and $Q = 4$ for WESAD EDA (10-dimensional); thus, the six-qubit ceiling is never reached. The operation at $Q \leq 6$ keeps the gradient variance large enough for stable optimization while matching the physiologically meaningful branch count; barren plateaus are further mitigated by the shallow depth ($L = 3$), a structured non-random ansatz, and near-identity initialization.

3.3.5. Simulation and Device Noise

The statevector at $Q \leq 6$ has at most $2^6 = 64$ amplitudes, so the circuit runs exactly, with PyTorch backpropagation, on PennyLane's default.qubit [64]. Expectation values are exact, and results correspond to the infinite-shot limit, while on hardware they would be estimated from finite shots with parameter-shift gradients [65,66]. The circuit at this scale is classically simulable [22] and we do not claim any quantum speed-up [67]. To demonstrate that this behavior is not an artifact of noiseless simulation, we retrain and evaluate the model on default mixed with a depolarizing channel of probability $p = 0.01$ on each qubit after each layer, so that noise acts during both training and inference; features, graphs, depth and schedule are unchanged, with the epoch budget reduced to 12 for tractability (Section 4.10).

3.4. Hybrid Head, Subject-Adversarial Invariance and Loss

The readout q_i is concatenated with a classical context vector $c_i = \mathrm{MLP}_\theta(\tilde{x}_i^{(K)}) \in \mathbb{R}^{64}$ from the full mRMR-selected vector, and passed through a dropout-regularized dense softmax head. We encourage cross-subject generalization using a gradient-reversal subject discriminator [35] ramped to $\lambda_{\max} = 0.5$, and address imbalance with focal loss ($\gamma = 2$, label smoothing 0.05) [68] with class-balanced sampling. We jointly optimize parameters with Adam [69] (5×10^{-4}, weight decay 2×10^{-3}, clipping 1.0), warm-up then cosine decay, for up to 50 epochs with early stopping on validation balanced accuracy (patience 10). Supplementary Section S4 presents the schedules.

The Structure-Matched Classical Twin and Its Capacity

To isolate the quantum core, we define a structure-matched control, replacing the variational circuit with a symmetric normalized graph convolution over the same super-node graph [70]. The graph, features, depth, and schedule are the same. Only the encoder is different. The two encoders are not matched in parameter count, and we state the counts exactly. The variational circuit has $L[3Q + \binom{Q}{2}]$ trainable parameters—75 at $Q = 5$ and 54 at $Q = 4$—and a readout of $Q + \binom{Q}{2}$ expectations. The graph convolution lifts each scalar super-node signal to a hidden width of 48, applies three propagation layers, and projects back to one value per node, which is $2h + L(h^2 + h) + h + 1 = 7201$ parameters at $h = 48$ with a readout of Q values. The rest of the network is shared; the only other difference is the first layer of the head, which reads $\binom{Q}{2}$ additional correlator inputs from the quantum encoder (640 weights at $Q = 5$, 384 at $Q = 4$). Excluding the subject discriminator, the full models total 11,160 (quantum) against 17,646 (twin) parameters for the HRV encoders at $Q = 5$ (Supplementary Section S13). The comparison is therefore conservative with

respect to the quantum encoder: any difference is attributable to the representation, not to a capacity advantage, because the classical encoder has the larger budget. To close the remaining gap in the other direction, we also report a strictly parameter-matched twin with $h = 4$, whose encoder has 73 parameters, as an additional ablation variant (Section 4.6).

3.5. Leakage-Free Preprocessing and Evaluation Protocols

Each data-dependent step is fit in each training fold T and applied to the held-out fold, so that no statistic computed on the test fold informs training. The steps are median imputation and per-subject z-scoring for subjects with at least two windows, which removes baseline offsets that would otherwise dominate the cross-subject gap. Then a Yeo–Johnson power transform [71] is applied and followed by standardization. After this, minimum redundancy–maximum relevance selection ($K = 24$) in F-statistic correlation-quotient form [72] is performed. The super-node signals come from the same in-fold standardized matrix; hence, the quantum branch has this guarantee.

One step deserves to be stated explicitly rather than left implicit. Per-subject z-scoring is applied to the held-out subject as well, using that subject's own windows. No label and no information from any other subject enters, so it introduces no train–test leakage. It does mean, though, that each new user's statistics come from that user's whole unlabeled recording, which spans all conditions; a deployed monitor would need an equivalent unlabeled calibration period before its first prediction. We report it because the same scrutiny we apply to other people's protocols applies to ours.

The architecture is then evaluated under three protocols. The honest protocol uses leave-one-subject-out or subject-grouped k-fold with no window overlap and confines all learned preprocessing, calibration included, to the training fold. The subject-dependent protocol uses random stratified k-fold ignoring subject identity, reproducing commonly reported optimistic figures. The window-overlap (overlap-leak) protocol additionally injects near-duplicate windows (each row kept and joined by two jittered copies, Gaussian noise 0.02 in standardized units) before an arbitrary split.

Three departures from strict identity belong on the record. Per-subject z-scoring runs only under the honest protocol, where subjects are grouped; the leakage protocols standardize without it and so retain between-subject offsets, which makes the reported gap a measurement of the conventional leaky pipeline rather than of the split alone. The subject-adversarial branch is active only under the honest protocol: subject-invariance is not the quantity the leakage protocols measure, and enforcing it there would suppress the memorization they exist to expose. The leakage arms also run one seed rather than three; they are demonstrations that inflation occurs, not estimates of its size, and the honest protocol carries the full seed set throughout. The training budget is matched at 50 epochs across all three, so no training-budget confound enters. Since an active adversary could only lower a leaky score, the reported gap is an upper bound in that respect.

The leakage gap—inflated minus honest performance on the identical model—renders the field's optimism measurable. Strong classical baselines run under every protocol on identical in-fold features: a class-balanced random forest and an isotonic-calibrated histogram gradient-boosting classifier, whose implementation follows LightGBM [73]. The gap is therefore a property of the protocol rather than of one model. The classical twin of Section 3.4, not an unrelated deep model, is the controlled comparison for the encoder itself.

Baseline Configuration

Neither baseline is tuned. The random forest uses 300 trees, class weight balanced by inverse class frequency, and the scikit-learn defaults for every other setting; the gradient-

boosting classifier uses a learning rate of 0.06, at most 400 boosting iterations, an L_2 penalty of 1.0 and early stopping, with all remaining settings at their defaults. These values were fixed before any protocol was run and are identical across datasets, modalities, folds, seeds and protocols, so no hyperparameter search, nested or otherwise, exists in the pipeline, and no tuning channel can carry subject information across the split. The same holds for AB-QGN, whose settings in Section 3.3 were likewise fixed in advance. Two internal splits inside the training fold are not subject-grouped and we name them. The gradient-boosting early-stopping criterion is scikit-learn's own, a random 10% validation subset of the training fold, and the isotonic calibrator of the reported baseline uses three-fold cross-fitting; the second of these is deliberate: it is the conventional practice whose calibration leakage Section 4.3 measures. The first affects only the number of boosting iterations, and on these data it barely does that: on the random subset the stopping criterion fires in only 5 of the 60 folds and never before iteration 359, so the fold-mean iteration count runs 394–400 of the 400, so the reported baseline is, to within 0.2 macro-F1 in every cell, the model with early stopping disabled. Selecting the iteration count instead on a subject-disjoint 10% split, by the minimum of the staged log-loss, and refitting on the full fold, gives a best iteration of 7–32 on average, with a per-fold range of 1 to 84, because the cross-subject log-loss begins to rise almost at once even though discrimination keeps improving, and it lowers honest macro-F1 by 0.4–7.0 points (significant after Holm correction in one of the four cells, Supplementary Section S14). The subject-sharing split therefore did not inflate the reported baseline; it left it the stronger of the two, and the same subject sharing that hides calibration leakage also hides the point at which cross-subject confidence stops improving. AB-QGN's own validation split, used for early stopping and for the temperature, is drawn subject-disjointly by GroupShuffleSplit.

3.6. Post Hoc Calibration and Late Fusion

After training we use temperature scaling [37], fitting a single scalar $T > 0$ on the held-out validation split by minimizing the negative log-likelihood and applying it to the test logits,

$$\hat{p}_i^{\text{cal}} = \text{softmax}(z_i/T), \qquad T^\star = \arg\min_{T>0} \Big[-\tfrac{1}{|V|} \textstyle\sum_{i \in V} \log \text{softmax}(z_i/T)_{y_i} \Big]. \tag{5}$$

Since Equation (5) is argmax-invariant, the accuracy and macro-F1 will remain unchanged, but the ECE and NLL will improve. Its single free parameter is robust to fit even on small validation folds.

The branches are combined at the session level, one session per subject × condition. Out-of-fold probabilities are averaged within each session, and the HRV and EDA session matrices are merged by inner join. That leaves 45 WESAD sessions (15 per class) and 51 SWELL-KW sessions (21 no-stress, 13 time-pressure, 17 interruption)—fewer than the 63 the 21 electrodermal subjects would allow, because the join is not complete on every subject–condition pair. A class-weighted logistic-regression meta-learner is then trained on the concatenation under subject-grouped k-fold, so it never sees a session from a subject in its own training fold. A single global temperature is finally fitted to the fused probabilities. We state plainly that this fit is in-sample: with 45 and 51 sessions, per-fold fitting produced degenerate temperatures on nine-session subfolds. Temperature scaling is argmax-invariant, so fused macro-F1, accuracy, and AUC are unaffected. The fused ECE and NLL of Tables 2 and 3 are nonetheless optimistically biased, and we rest no calibration claim on them; every calibration comparison in Section 4.3 is at the window level, with the calibrator fitted strictly out-of-sample and subject-disjoint. Session averaging gives a clinically meaningful decision granularity and reduces window-level noise.

Table 2. Honest five-fold results. Acc = accuracy; BalAcc = balanced accuracy; AUC = one-vs-rest macro AUC; ECE = expected calibration error. Single-modality rows are window-level fold means with ± the standard deviation over three seeds × five folds; fused rows are session-level with ± a bootstrap standard error, so the two are not comparable (Section 4). The window-level calibration analysis of Section 4.3 reports ECE pooled over held-out windows instead and should be preferred for calibration comparisons. Bold marks the session-level fused AB-QGN figures, which are the headline result; the fused ECE is not set in bold because its temperature is fitted in-sample and carries no calibration claim (Section 3.6).

Dataset	Modality	Acc	BalAcc	Macro-F1	AUC	ECE
WESAD	HRV	66.3 ± 9.6	63.8 ± 11.1	62.5 ± 10.3	80.6 ± 8.1	11.9 ± 3.9
WESAD	HRV (RF)	78.6 ± 4.8	69.7 ± 5.3	71.0 ± 5.4	86.3 ± 5.2	6.3 ± 1.8
WESAD	HRV (GBM)	77.9 ± 6.2	70.0 ± 6.5	71.3 ± 6.3	83.1 ± 4.6	16.0 ± 4.8
WESAD	EDA	75.0 ± 9.9	66.8 ± 9.5	66.3 ± 10.3	81.8 ± 11.0	15.6 ± 8.2
WESAD	EDA (RF)	76.3 ± 9.3	63.5 ± 6.8	62.0 ± 6.7	85.3 ± 8.7	12.9 ± 4.2
WESAD	EDA (GBM)	76.7 ± 8.0	63.4 ± 6.7	61.8 ± 6.7	82.0 ± 8.5	18.1 ± 7.4
WESAD	**Fused**	**86.7 ± 5.0**	**86.7 ± 4.5**	**86.2 ± 5.2**	**88.6 ± 4.5**	17.7 ± 4.6
SWELL	HRV	58.8 ± 6.0	51.2 ± 7.3	49.0 ± 6.2	74.2 ± 4.4	10.9 ± 5.0
SWELL	HRV (RF)	66.5 ± 6.0	59.1 ± 8.9	53.6 ± 7.7	80.7 ± 5.3	10.2 ± 2.0
SWELL	HRV (GBM)	64.5 ± 5.0	53.1 ± 6.3	51.8 ± 7.4	74.5 ± 4.6	25.9 ± 5.0
SWELL	EDA	43.8 ± 5.1	38.7 ± 7.7	36.8 ± 5.8	55.5 ± 6.0	9.2 ± 2.9
SWELL	EDA (RF)	51.2 ± 4.8	39.6 ± 6.1	37.8 ± 5.3	54.5 ± 8.9	12.9 ± 5.8
SWELL	EDA (GBM)	48.9 ± 5.0	40.2 ± 6.7	40.0 ± 5.8	60.5 ± 3.8	39.3 ± 5.0
SWELL	**Fused**	**66.7 ± 6.3**	**62.0 ± 6.4**	**61.4 ± 6.6**	**77.8 ± 4.6**	11.8 ± 4.7

Table 3. Honest LOSO results. As in Table 2, the ± is a seed-and-fold standard deviation for single-modality rows and a session-level bootstrap standard error for fused rows. The corresponding fused macro-F1 figures are 84.0 ± 10.6 on WESAD and 55.1 ± 13.0 on SWELL-KW, the ± being the half-width of the 95% bootstrap interval. Bold marks the session-level fused AB-QGN figures. The fused ECE is not set in bold, for the reason given in Table 2.

Dataset	Modality	Acc	BalAcc	Macro-F1	AUC	ECE
WESAD	HRV	67.3 ± 15.2	64.3 ± 15.5	63.1 ± 14.8	81.1 ± 12.8	12.9 ± 7.4
WESAD	EDA	75.7 ± 21.5	68.5 ± 21.7	67.6 ± 21.7	83.9 ± 18.1	19.0 ± 11.9
WESAD	**Fused**	**84.4 ± 5.4**	**84.4 ± 5.1**	**84.0 ± 5.5**	**90.4 ± 3.8**	12.5 ± 4.4
SWELL	HRV	59.5 ± 14.7	52.6 ± 16.1	44.8 ± 13.2	74.3 ± 16.1	17.1 ± 7.8
SWELL	EDA	45.2 ± 13.7	42.6 ± 13.5	35.1 ± 11.5	57.0 ± 14.2	18.1 ± 6.6
SWELL	**Fused**	**60.8 ± 6.9**	**56.3 ± 6.3**	**55.1 ± 6.6**	**78.8 ± 4.4**	11.3 ± 4.9

3.7. Metrics, Statistics and Reproducibility

We report macro-F1, accuracy, balanced accuracy, and one-vs-rest macro AUC, with ECE on 15 equal-mass bins [74], NLL, and the multiclass Brier score. Macro and balanced metrics are emphasized because of class imbalance, and the proper scoring rules accompany ECE because they remain reliable where binned ECE does not. Honest results average over three seeds $\{42, 7, 123\}$ and the folds of the protocol (five for subject-grouped k-fold; 15, 21, and 22 for LOSO, the electrodermal SWELL-KW branch having 21 subjects). Comparisons use paired Wilcoxon signed-rank tests over matched per-fold scores (15 pairs under five-fold; 45, 63, or 66 under LOSO) with Holm correction and Cliff's δ. The fused decision has no fold structure, so its dispersion comes from a session-level bootstrap (1000 resamples) and, for the headline figures, a 10,000-permutation label test. Two further tests support the comparison with the classical twin. At window level, macro-F1 is compared with the same paired Wilcoxon signed-rank test over the 15 matched fold × seed pairs used for the calibration comparison with the twin (Section 4.3), with Holm correction and Cliff's δ. At session level, where the two fused decisions are paired on identical sessions, we report an exact McNemar test on the discordant sessions, a paired permutation

test that swaps the two models' decisions independently per session (10,000 swaps), and a paired session bootstrap of the macro-F1 difference (10,000 resamples). Session-level figures are computed from the out-of-fold probabilities of the first seed (42); the window-level rows average all three. Where a session-level quantity is compared across configurations (Sections 4.6 and 4.8), we now also fuse each seed separately and report the across-seed spread. Seeds, fold assignments, and the configuration are serialized, and every artifact regenerates exactly under the same run configuration; a rerun of the $h = 48$ twin under a different configuration differed at fold level (Supplementary Section S10). Experiments used PennyLane 0.45.0, PyTorch 2.12.0+cpu, and scikit-learn 1.9.0 on an Intel64 Family 6 Model 140 CPU.

3.8. Portability Check on a Third Corpus

The design above deliberately takes released feature tables as given, so that any difference between protocols is a property of the partition and not of a front end of ours (Supplementary Section S12 lists the candidate corpora, and the criterion each fails). To test the protocol findings beyond the two release corpora, we relax exactly that constraint, once, on AffectiveROAD [48], a corpus that meets the signal and subject-identifier criteria but offers a continuous stress rating instead of a three-class protocol (Supplementary Table S11). It was recorded on real roads with an Empatica E4 wristband and a Zephyr BioHarness chest strap and is distributed with a continuous stress metric rated by an observer in the car and validated by the driver afterwards. The public release, as processed here, provides nine drivers over thirteen drives of about 85 min each. From the Empatica E4 photoplethysmographic inter-beat-interval stream, we computed the heart-rate-variability feature definitions of the release on 60-s windows stepped every five seconds (8152 windows; 46–56 min of window anchors per drive; 30 of the 34 release columns), so the register realizes all five autonomic branches. Three departures from the release's own front end belong on the record, because they bound what this corpus can show. The beat source is wrist photoplethysmography rather than chest electrocardiography, the distinction drawn in Section 3.1, and the device reports intervals only where they pass its own quality filter, so the retained intervals cover a median of 48.7 of every 60 s (range 22.3–59.9) and the successive-difference indices (RMSSD, SDSD, pNNx) are computed across those gaps. The window is one minute rather than five, far below the record length the measurement standard asks for [63]: very-low-frequency power is exactly zero in more than half of the windows and contributes 3.9% of total power on average, so two of the five features of the sympathovagal branch (VLF and its percentage) are uninformative here. These are properties of this front end, not of the partition, and they are the reason we draw no encoder conclusion from this corpus. The electrodermal table carries nine release names for which our extraction has an approximate counterpart (for example, MEAN from the tonic level, MEAN_1ST_GRAD from the slope, the SCR amplitude mean and maximum for MEAN_PEAKS and MAX_PEAKS; the full mapping is part of the archived conversion utility), so the EDA register realizes four branches. The complexity branch is absent. The corpus carries no protocol condition per window in the files we used, so the three classes are bands of the stress metric fixed before any model was fitted—low ($<1/3$), medium, high ($\geq 2/3$), giving 809, 2322, and 5021 windows—the subject is the driver. The pipeline's session unit (subject $\times$ class) becomes a driver $\times$ band here, 27 in all; because a band is defined by the label, such a session pools non-contiguous windows already known to share a class and is not a unit a monitor could observe. Everything inside the pipeline is unchanged: the same preprocessing fitted per fold, the same AB-QGN, random-forest and gradient-boosting configurations, the same three protocols, and the same calibration-split experiment. The strict leave-one-subject-out regime is reported in

Supplementary Section S15 as a sensitivity check, and the conversion utility also implements the empirical tertiles and, through its two settable cut points, the discretization of Bustos et al. [75], neither of which we ran, for readers who wish to vary the discretization.

4. Results

Throughout, "honest" refers to the subject-disjoint protocol with all preprocessing, feature selection, and calibration fitted in each training fold. Window-level metrics aggregate three seeds, session-level metrics use out-of-fold, temperature-scaled probabilities. We report two regimes, subject-grouped five-fold and strict LOSO, and a finding is considered robust only when direction and significance survive Holm correction in both.

4.1. Honest Subject-Independent Performance

The honest five-fold performance is shown in Table 2. The session-level macro-F1 of bimodal late fusion is 86.2 on WESAD and 61.4 on SWELL-KW. Both figures are of the reference seed (42), whose out-of-fold probabilities feed every session-level analysis in this paper, fusing each of the three seeds separately gives 81.6 ± 4.8 on WESAD and 64.8 ± 4.5 on SWELL-KW (Section 4.8), so the reference-seed value is the highest of the three on WESAD and the lowest on SWELL-KW. These are realistic subject-independent values, far from the near-ceiling figures common in this literature, and that distance is the point of this work. The same gap is reported in the same way in recent work in this journal. A five-channel wearable electroencephalography benchmark pairs 99.94% window-level accuracy under a subject-dependent split with 66.70% session-level accuracy across unseen subjects and presents the former as an optimistic upper bound rather than a deployment estimate [76]. Our contribution is to run both protocols on one fixed pipeline so that the gap is measured rather than argued.

What those rows support is bounded by the 45 (WESAD) and 51 (SWELL-KW) fused sessions. The fused ECE is the largest of the AB-QGN rows in the five-fold table, but the smallest under LOSO (Table 3). A quantity that changes direction between regimes does not measure a stable property. Also, binned ECE is unreliable at this sample size, and the fused temperature is fitted in-sample as mentioned in Section 3.6, so we make no calibration claim on these rows. The $\pm$ on fused rows is a bootstrap standard error, not a spread across subjects—the 95% percentile bootstrap intervals are $[74.9, 95.3]$ and $[48.1, 73.6]$. What the session count does support is determined by permutation: against 10,000 permutations of the session labels, the observed macro-F1 is higher than every permutation drawn, 7.5 standard deviations above a permutation mean of 32.9 (WESAD) and 4.3 standard deviations above a permutation mean of 33.2 (SWELL-KW) ($p < 10^{-4}$).

The evaluation of strict LOSO is elaborately shown in Table 3. The figures are steady: WESAD fused macro-F1 changes from 86.2 to 84.0 and SWELL from 61.4 to 55.1, while fused WESAD AUC climbs to 90.4—not paradoxical, because LOSO splits more stringently but trains on more people per fold, 14 of 15 subjects on WESAD versus 12 under five-fold grouping—and all the differences between regimes are within the bootstrap intervals above. The per-held-out-subject distribution is wide, as evidenced in Figure 2: individual subjects span tens of macro-F1 points within one modality and dataset, with several folds below 40.

Confusion matrices (Figure 3) and ROC curves (Figure 4) show strength concentrating where the physiology is unambiguous—per-class F1 of 96.8 for WESAD stress, 88.4 for SWELL no-stress—while minority classes stay hard: 76.9 for WESAD amusement, 34.8 for SWELL time-pressure, falling to 18–38 at the single-modality level. The error structure is informative: on SWELL-KW, the dominant confusion is time-pressure against interruption, with 54% of time-pressure sessions being assigned to interruption while no-stress is recovered at 90%; the model separates stressed from unstressed states, then fails to identify

which stressor produced the response. Expressed as a monitoring service would meet it, and reading the fused probability against a decision threshold rather than by argmax, the model raises 2.2 unnecessary alerts per 100 sessions on WESAD at 93.3% sensitivity to stress (threshold 0.76) and 3.9 per 100 on SWELL-KW at 90.0% sensitivity to either stressor (threshold 0.67); the confusion matrices of Figure 3 are argmax decisions and therefore sit at a different operating point.

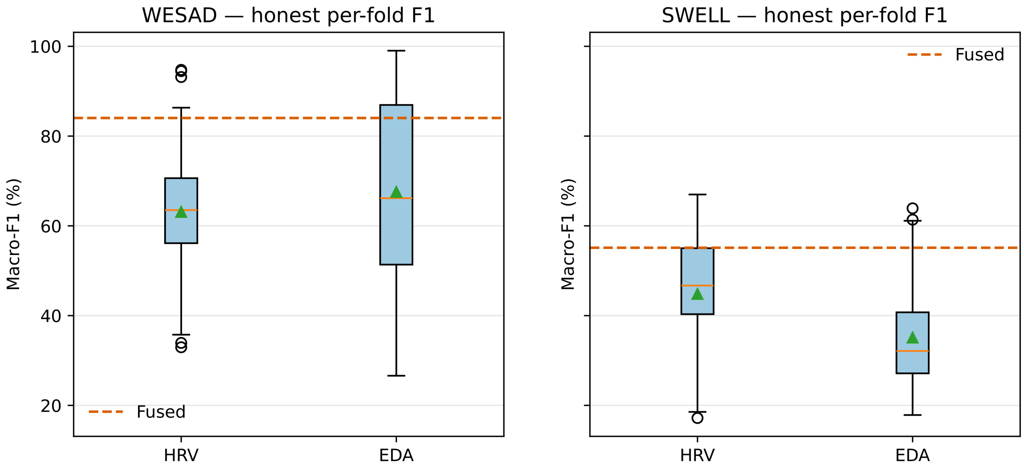

Figure 2. Per-fold macro-F1 distribution for each single modality under LOSO. Boxes represent the interquartile range, the orange line the median, the green triangle the mean, and circles the outlier folds; the dashed line marks the fused AB-QGN score. The per-subject spread is large for both single modalities, which is the honest picture of cross-individual variability in this problem. The operating point is lifted above the single-modality medians by late fusion.

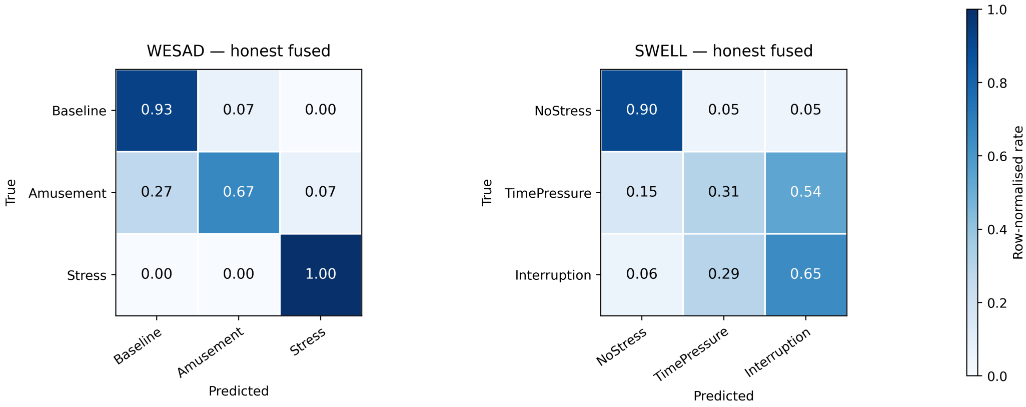

Figure 3. Confusion matrices for the fused AB-QGN under the honest protocol. Errors concentrate on the minority affective classes.

https://doi.org/10.3390/s1010000

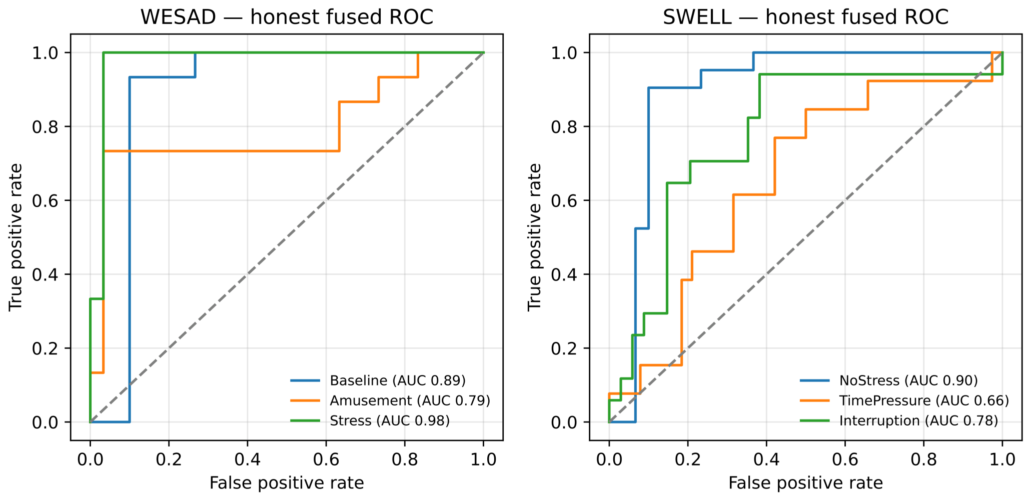

Figure 4. One-vs-rest ROC curves under the five-fold honest protocol. Per-class AUC values give a macro one-vs-rest AUC of 88.6 on WESAD and 77.8 on SWELL; under the stricter LOSO protocol, the fused WESAD AUC rises to 90.4. The dashed diagonal marks a chance-level classifier (AUC = 0.5).

4.2. The Magnitude of Evaluation Inflation

The central result is quantified in Table 4 and Figure 5: we re-ran the same encoder, features, and training budget under both leakage-inducing protocols, for each model and modality, and measured the macro-F1 inflation relative to the honest number. The table shows nine of fourteen rows for space. All fourteen rows, under both the five-fold and the LOSO regime, are tabulated in Supplementary Tables S9 and S10; they include three cells in which the single-modality encoder inflates more than the corresponding random forest: WESAD HRV under both regimes (five-fold +34.2 against +29.0; LOSO +33.6 against +31.0) and SWELL HRV under five-fold (+48.0 against +46.4), all on the subject-dependent split, which the window-overlap split orders the same way, so the reduction below is attributed to fusion rather than the encoder. The 27–61 point range quoted in the abstract is the span of the per-modality cells of those two tables.

Table 4. Evaluation-inflation gap in macro-F1 points—five-fold. Inflation is the leaky score minus the honest score; larger values indicate greater dependence on data leakage for evident performance. Bold marks the session-level fused AB-QGN rows. All other rows are window-level single-modality configurations.

Dataset	Modality	Honest	Subject-Dependent (Δ)	Window-Overlap (Δ)
WESAD	HRV	62.5	+34.2	+34.1
WESAD	HRV (RF)	71.0	+29.0	+29.0
WESAD	HRV (GBM)	71.3	+28.7	+28.7
WESAD	EDA	66.3	+28.6	+28.7
WESAD	**Fused**	**86.2**	**+13.8**	**+13.8**
SWELL	EDA (RF)	37.8	+58.3	+58.6
SWELL	EDA (GBM)	40.0	+56.6	+56.0
SWELL	HRV (RF)	53.6	+46.4	+46.4
SWELL	**Fused**	**61.4**	**+38.6**	**+38.6**

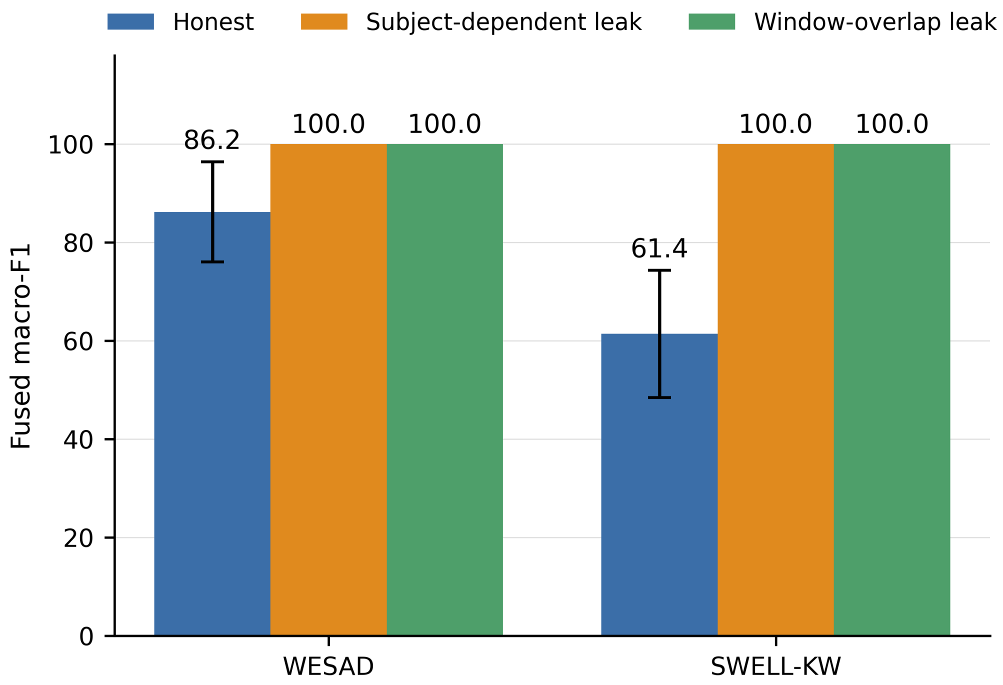

Figure 5. Honest versus inflated macro-F1 for the fused AB-QGN. The leakage-free five-fold score is shown as well as the subject-dependent and window-overlap scores obtained from the same model and features, both saturating at 100. Error bars are ±1.96 session-level bootstrap standard errors on the honest score. This panel is fused. The 27–61 point range quoted in the abstract refers to the per-modality cells across both regimes. The per-model and per-modality inflation, including the classical baselines, is reported in Table 4.

Two facts stand out. The classical baselines inflate most: random forest and gradient boosting reach 96–100% macro-F1 under leaky protocols on both datasets and fall by 28–59 points once subjects are held out, so a practitioner reading only the leaky numbers would judge them near-perfect. And the fused AB-QGN inflates least of any configuration, 13.8 points on WESAD—though only because its honest score is the highest, not because its leaky score is lower: the fused model reaches exactly 100.0 on both corpora under both leaky protocols, as do four of the remaining twelve configurations (six of fourteen in total), with a seventh saturating under one of the two. At the ceiling, the leaky protocol separates no two models at all, which is the sharpest sense in which the protocol, and not the architecture, fixes the reported number.

The gap remains under LOSO: WESAD fused +16.0, SWELL classical baselines +52 to +61. That confirms it as a property of the problem and the models rather than of the cross-validation choice. Independently, the authors of this feature set report the same effect on the same SWELL HRV features of their own construction—a person-specific model at 95.2 ± 0.5% accuracy under ten-fold cross-validation within each subject, against a generic model at 42.5 ± 19.9% under LOSO [47].

4.3. Calibration Leakage: The Second Face of the Same Problem

Calibration invites three comparisons, and the first turns out to measure the evaluation protocol rather than the model. Post hoc calibrators are themselves fitted on a held-out split of the training fold. We fit AB-QGN's temperature on a subject-disjoint split (Section 3.5); the gradient-boosting baseline, following common practice, uses ordinary k-fold in the training fold, so its calibration folds share subjects with its fitting folds. Refitting it subject-disjoint changes it out of recognition (Table 5): over three seeds, ECE falls from 15.84 ± 0.21 to 4.06 ± 0.83 on WESAD HRV, 16.55 ± 0.38 to 9.23 ± 0.71 on WESAD EDA, 25.56 ± 0.14 to

5.39 ± 0.48 on SWELL-KW HRV, and 39.06 ± 0.24 to 6.56 ± 0.74 on SWELL-KW EDA, gaps of 13 to 59 pooled standard deviations. Isotonic regression is affected as strongly as temperature scaling (15.84 → 5.54, 39.06 → 7.62), so this is the split and not the method, and Figure 6 shows the departure it produces. All four method-by-split combinations, and the full distribution of fitted temperatures across folds, are tabulated in Supplementary Section S9.

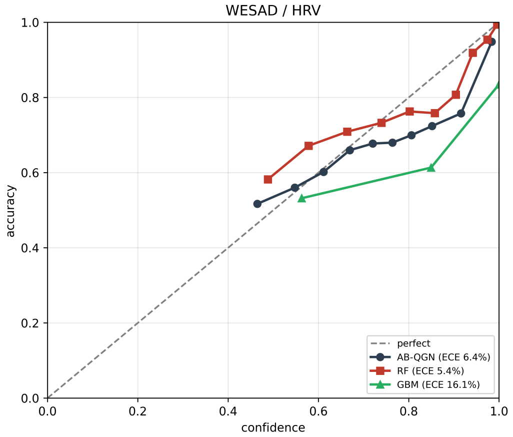

Figure 6. Reliability diagrams for the heart-rate-variability encoders under the honest five-fold protocol, pooled over held-out windows: temperature-scaled AB-QGN against random forest and the conventionally calibrated gradient-boosting baseline, whose calibrator uses the same subjects in its fitting and calibration folds. The diagonal is perfect calibration. Gradient boosting departs from it markedly; AB-QGN and random forest are close to it and trade places between datasets. Refitting the gradient-boosting calibrator subject-disjointly removes most of this departure (Table 5). Expected calibration errors in the legend are the pipeline's own window-level values; the gradient-boosting rows of Table 5 come from the three-seed reimplementation and differ by at most 0.4 ECE.

Table 5. Calibration at window level under the honest five-fold protocol, pooled over held-out windows. Gradient boosting appears twice: with its calibrator fitted by ordinary k-fold in the training fold, which is conventional practice, and with a subject-disjoint calibration split. The two rows pair the deployed default (isotonic, the pipeline's baseline calibrator) against the correction we recommend (temperature on a subject-disjoint split); Supplementary Section S9 crosses both calibrator families with both splits, and the conclusion is unchanged in all four combinations. Lower is better throughout; bold marks the best model in each block. Both gradient-boosting rows are means over three seeds from the same implementation, which reproduces the pipeline's conventionally calibrated figure to within 0.4 ECE. The subject-disjoint rows fit one base model on 80% of the training fold rather than ensembling three, which changes macro-F1 by −2.8 to +4.4 points. The calibration gain is not attributable to a stronger base model: discrimination falls in three of the four cells, and in the fourth a 4.4-point macro-F1 gain accompanies a 7.3-point ECE improvement that a change of that size cannot produce.

Dataset	Modality	Model	ECE	NLL	Brier
WESAD	HRV	AB-QGN	6.39	0.75	0.44
		RF	5.40	0.65	**0.33**
		GBM, isotonic, non-grouped	15.84	3.52	0.38
		GBM, temperature, subj.-disjoint	**4.06**	**0.62**	0.35
WESAD	EDA	AB-QGN	10.65	1.01	0.40
		RF	10.72	1.60	0.36
		GBM, isotonic, non-grouped	16.55	3.78	0.40
		GBM, temperature, subj.-disjoint	**9.23**	**0.66**	**0.35**
SWELL	HRV	AB-QGN	**4.22**	0.95	0.53
		RF	6.59	1.07	**0.46**
		GBM, isotonic, non-grouped	25.56	6.26	0.60
		GBM, temperature, subj.-disjoint	5.39	**0.82**	0.47
SWELL	EDA	AB-QGN	**6.05**	1.10	0.66
		RF	11.64	1.22	0.65
		GBM, isotonic, non-grouped	39.06	4.50	0.88
		GBM, temperature, subj.-disjoint	6.56	**1.05**	**0.63**

The mechanism is visible in the fitted temperature. Across 60 folds on a subject-sharing split, it never exceeds 1.30 and sits at the grid floor of 0.05 in 36, so calibration sharpens an already overconfident model; subject-disjoint, it never falls below 2.81 and reaches the ceiling of 6.00 in 29, so even the available correction is insufficient. Two orders of magnitude from the split alone, and the failure is invisible because the calibrator's validation set agrees with it: both come from subjects the model has seen. Leakage therefore has a second face—it inflates accuracy, as Section 4.2 quantifies, and it silently corrupts confidence, which for a monitor acting on a probability is the more consequential.

4.3.1. Against Gradient Boosting

Once the baseline's calibrator is fitted subject-disjoint, the comparison reverses: it attains lower NLL and Brier than AB-QGN in all four cells and lower ECE in two, and where the temperature grid saturates, it is under-corrected, so the true margin is wider. We therefore withdraw any claim of a calibration advantage over gradient boosting. The advantage measured against a conventionally calibrated baseline is a real statement about deployed pipelines and an empty one about encoders.

4.3.2. Against Random Forest

Random forest, left uncalibrated, is the better-calibrated baseline. Its window-level ECE is lower than AB-QGN's on WESAD HRV, 5.40 against 6.39, and similar on WESAD EDA. After Holm correction it is significantly better on ECE in two cells and Brier in four

across the two regimes. Across three metrics and both regimes, AB-QGN is significantly better in one out of twenty-four cells and significantly worse in seven.

4.3.3. Against the Structure-Matched Classical Twin

The twin has the full pipeline, including the calibration split, so any difference is attributable to the encoder rather than to features, optimization, or protocol, and, since the twin's encoder carries the larger parameter budget (Section 3.4), not to a capacity advantage of the quantum side (Table 6). ECE is better in three of four cells for the quantum encoder, significantly so on SWELL-KW HRV after Holm correction, and the twin is never significantly better. NLL and Brier differences are non-significant throughout (all $p_{\mathrm{Holm}} \geq 0.78$). Against the parameter-matched twin ($h = 4$), the direction holds in three of four cells (Δ ECE -1.74, -0.99, -0.39; $+0.70$ on WESAD HRV), but no cell is significant (smallest $p_{\mathrm{Wilcoxon}} = 0.073$, $p_{\mathrm{Holm}} = 0.22$). This is the only calibration claim we make for the encoder itself, and it is a modest one: a directional ECE advantage over a structure-matched control, significant in one cell of four and in none against the parameter-matched one.

Table 6. AB-QGN vs. the structure-matched classical twin on expected calibration error, honest protocol, paired over identical folds and seeds. Both employ the same subject-disjoint calibration split. Δ is AB-QGN minus twin, so negative values favor AB-QGN. Values are per-fold means over the 15 fold $\times$ seed pairs, as the paired test requires, and are therefore not comparable in level with the window-pooled figures of Table 5. Bold marks the lower ECE of the two encoders in each row and the only Holm-adjusted p-value below 0.05.

Dataset	Modality	AB-QGN	Twin	Δ	p_{Wilcoxon}	p_{Holm}	Cliff's δ
SWELL	HRV	**10.90**	12.87	-1.97	0.0043	**0.0128**	-0.30
WESAD	EDA	**15.62**	17.66	-2.05	0.0215	0.0646	-0.17
SWELL	EDA	**9.25**	10.09	-0.84	0.3591	1.0000	-0.15
WESAD	HRV	11.93	**10.72**	$+1.21$	0.1205	0.3616	$+0.19$

4.3.4. What Calibration Buys Operationally

The consequence is seen in where a threshold can sit. To achieve 90% sensitivity, AB-QGN needs a threshold between 0.27 and 0.57—a plausible range for a clinician—while the conventionally calibrated baseline requires 0.05 on WESAD HRV and effectively zero on both SWELL-KW modalities, reaching that sensitivity only by raising false alerts on 82% and 100% of non-stress windows. Random forest, which has better discrimination, generates fewer false alerts at matched sensitivity in three of four cells: the difference in calibration appears not in the number of false alerts at a matched operating point, which is rank-based and invariant to monotone recalibration, but in whether a threshold can be chosen at all.

4.4. Discrimination

On macro-F1, AB-QGN differs significantly from neither tree ensemble in most cells and is behind on WESAD HRV (five-fold -8.9 against GBM, $p_{\mathrm{Holm}} = 0.0024$, and -8.6 against RF, $p_{\mathrm{Holm}} = 0.014$; LOSO -6.1 against GBM, $p_{\mathrm{Holm}} = 0.0008$, and -5.9 against RF, $p_{\mathrm{Holm}} = 0.034$). On WESAD EDA it leads by 4.3–4.5 macro-F1 under five-fold, and by 1.9–4.0 under LOSO, in neither case significantly; on both SWELL modalities differences are small and non-significant in both regimes. Against training-cohort size (Supplementary Section S6) it sits 1.5 macro-F1 below the better classical baseline on average, narrowing to 0.35 at $k = 3$ against 1.95 at $k \geq 5$, and at $k = 3$ it leads in two of four cells—parity in the low-subject regime, not a data-efficiency advantage. The cohort-size experiment runs the encoder without the subject-adversarial branch, so these rows describe the ablated variant rather than the full model of Tables 2 and 3.

4.5. Comparison with Prior Work Under Matched Protocols

Read against protocol, the comparison is favorable. Prior quantum and hybrid models evaluated without leakage fall into the AB-QGN regime rather than the near-ceiling one—52.1% three-class LOSO accuracy on WESAD HRV [15]; quantum-kernel and tree-tensor-network models reach 45–55% on a held-out split whose relation to subject identity is not stated [29]—whereas the near-perfect figures (99.97–99.99% [15]; F1 0.9999 [28]; 94.5–97.8% [43]) come from subject-dependent or window-overlapping splits, or from splits for which no subject-level partitioning is reported. Our matched comparison, WESAD HRV LOSO accuracy of 67.3% (Table 3), exceeds the 52.1% reference by 15 points on the same data under the same protocol. Supplementary Section S5 tabulates every entry with its protocol; none reports calibration.

4.6. Ablation and Margin over the Classical Twins

Table 7 and Figure 7 present the fusion-level ablation: each component is removed, and honest macro-F1 is remeasured. Table 8 tests the margin to the classical twins at window and session levels. The classical twin appears twice: the structure-matched twin of Section 3.4 (hidden width 48, 7201 encoder parameters) and the strictly parameter-matched twin (hidden width 4, 73 encoder parameters against the quantum encoder's 75).

Table 7. Ablation at the fusion level (honest macro-F1; Δ against the full model). Δ is computed on unrounded values and may therefore differ from the difference of the two rounded entries by 0.1. Bold marks the full model, against which every Δ is computed.

Variant	WESAD Fused	Δ	SWELL Fused	Δ
Full AB-QGN	**86.2**	—	**61.4**	—
− Entanglement (IsingZZ)	81.2	−5.0	58.3	−3.1
− ZZ readout correlators	81.2	−5.0	58.4	−3.0
− Data re-uploading	83.7	−2.5	63.2	+1.8
− Adversarial branch	83.7	−2.5	56.3	−5.1
− mRMR feature selection	81.2	−5.0	63.2	+1.8
− Focal loss (→ cross-entropy)	80.9	−5.3	49.7	−11.8
Classical twin, structure-matched ($h = 48$)	83.7	−2.5	52.0	−9.4
Classical twin, parameter-matched ($h = 4$)	81.1	−5.0	58.1	−3.3

At the fusion level, the quantum components carry material weight: removing entanglement costs 5.0 macro-F1 on WESAD and 3.1 on SWELL; the ZZ correlators 5.0 and 3.0; and the adversarial branch 5.1 on SWELL, while replacing focal loss with cross-entropy is severely damaging on SWELL (−11.8); the structure-matched twin loses 9.4 on SWELL and 2.5 on WESAD. The two quantum ablations are nested—the first removes the IsingZZ gates and with them the $\langle Z_a Z_b \rangle$ readout, the second keeps the gates and drops only the readout terms—and their near-identical cost is consistent with the correlators being the channel through which entanglement reaches the classifier.

Three caveats belong with these numbers. Two variants improve SWELL fused macro-F1 when removed (data re-uploading and mRMR, both +1.8), and we report that as measured rather than only where the direction favors us. Per-modality effects are small, not individually significant after Holm correction ($p_{\text{Holm}} = 1.0$ throughout), and not in the direction of the fused deltas: removing the entanglers or the correlator readout raises window-level macro-F1 slightly in all four cells (+0.04 to +1.48). The fusion-level deltas are single-point estimates. Over 45 and 51 sessions, a delta near 2.5 macro-F1 is roughly one session changing class, which is why several variants land on the same value. For the twin rows, this is no longer an untested statement. Table 8 pairs the fused decisions session by session, but it still holds for component ablations, for which we did not run one.

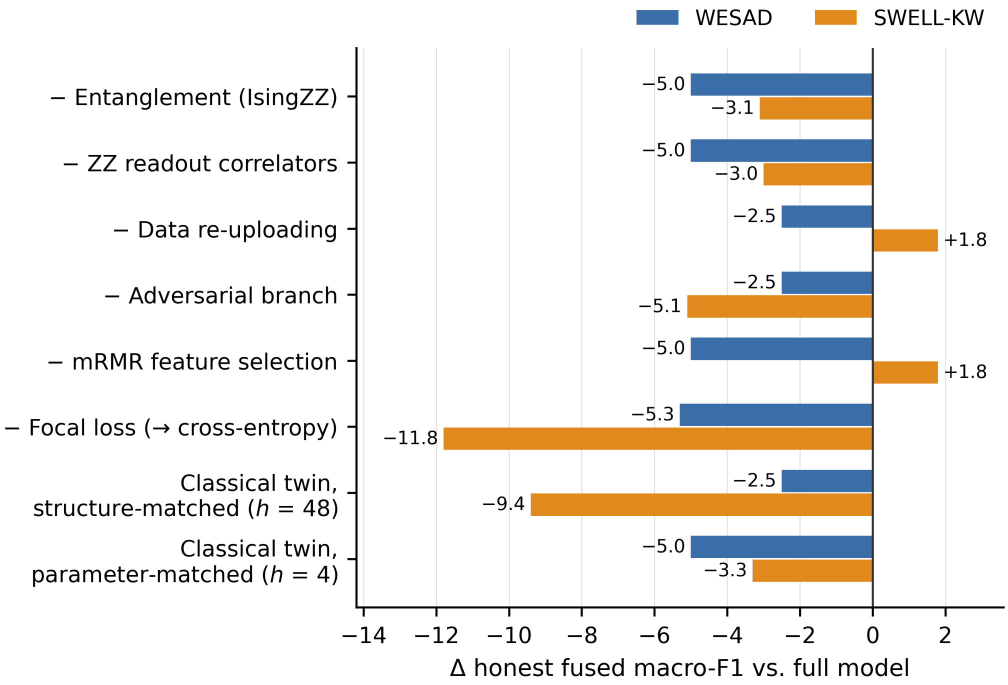

Figure 7. Honest fused macro-F1 change when each component is dropped, the level at which the model actually operates. The session-level score is not continuous when it is calculated over 45 sessions, and therefore several WESAD deltas are exactly the same (−2.5, −5.0): equal deltas correspond to the same number of flipped session decisions. The granularity is a property of the session count, and not of the ablation. Both classical twins of Table 7 are shown. The parameter-matched twin ($h = 4$) comes from the later run that introduced that variant.

The margin over the classical twin, which the abstract quotes, is now tested to the same standard as the calibration comparison of Table 6 (Table 8). At window level, over the 15 matched fold × seed pairs per cell, the two encoders are at parity: the structure-matched twin is marginally ahead in three of four cells (differences between −2.2 and +0.1 macro-F1 points, Table 8) and the parameter-matched twin in all four (between −2.2 and −0.4), and no difference approaches significance (smallest $p_{\text{Holm}} = 0.48$; Cliff's δ between −0.24 and +0.02). At session level, where the fused decisions of two models are paired on identical sessions, the SWELL-KW margin of 9.4 points over the structure-matched twin corresponds to eight sessions decided differently, six in favor of AB-QGN and two in favor of the twin (exact McNemar $p = 0.29$, paired-swap permutation $p = 0.22$, paired bootstrap 95% interval [−4.1, +22.5]); the 3.3-point margin over the parameter-matched twin to five sessions, three against two (exact McNemar $p = 1.0$, paired-swap $p = 0.56$). On WESAD the 2.5-point margin over the structure-matched twin is a single session, and the parameter-matched twin ties AB-QGN across seeds. Fusing each seed separately, AB-QGN is ahead of both twins on SWELL-KW at every seed—by +9.4, +3.6, and +4.8 points over the structure-matched twin (mean +5.9) and by +3.3, +12.1 and +8.6 over the parameter-matched twin (mean +8.0; the seed-7 margin is the only one whose bootstrap interval excludes zero, [+2.5, +22.9], McNemar $p = 0.062$ and paired-swap $p = 0.031$, a nominal value that does not survive correction over the twelve seed × twin × corpus comparisons)—whereas on WESAD the margins are +2.5, 0.0 and +7.4 (mean +3.3) and +5.0, −0.2 and −4.8 (mean 0.0). We therefore describe the quantum core's contribution as a session-level advantage on SWELL-KW that is consistent in direction across seeds and twins but not significant at 51 sessions, no advantage on WESAD against a parameter-matched encoder, and parity per window, and we have removed every wording that implied more.

Table 8. AB-QGN vs. the classical twins on macro-F1, honest five-fold protocol. Window level: paired over the identical 15-fold × seed pairs per cell used in Table 6; Δ is AB-QGN minus twin, so positive values favor AB-QGN, with a bootstrap 95% interval on the mean paired difference. Session level: fused decisions paired on identical sessions (first seed); b/c are the sessions decided correctly only by AB-QGN/only by the twin, p_{McN} the exact McNemar test, p_{swap} the paired-swap permutation test, and the interval a paired session bootstrap of the macro-F1 difference. Per-seed values for seeds 7 and 123 are given in the text. The table has two panels, each introduced by an italic label row followed by its own column headers. In the lower panel, Variant gives the hidden width h of the twin.

Window level (structure-matched twin, $h = 48$)							
**Dataset**	**Modality**	**AB-QGN**	**Twin**	**Δ**	**95% CI**	p_{Holm}	**Cliff's δ**
WESAD	HRV	62.5	63.3	−0.9	[−3.0, +1.2]	1.00	−0.08
WESAD	EDA	66.3	68.5	−2.2	[−5.9, +1.5]	0.48	−0.11
SWELL	HRV	49.0	49.3	−0.3	[−2.6, +1.9]	1.00	−0.03
SWELL	EDA	36.8	36.7	+0.1	[−2.2, +2.5]	1.00	−0.00
Session level (fused decisions, 45 and 51 sessions)							
**Dataset**	**Variant**	**AB-QGN**	**Twin**	**Δ**	b/c	$p_{\mathrm{McN}}/p_{\mathrm{swap}}$	**95% CI**
WESAD	$h = 48$	86.2	83.7	+2.5	1/0	1.00/1.00	[0.0, +8.4]
WESAD	$h = 4$	86.2	81.1	+5.0	2/0	0.50/0.50	[0.0, +13.1]
SWELL	$h = 48$	61.4	52.0	+9.4	6/2	0.29/0.22	[−4.1, +22.5]
SWELL	$h = 4$	61.4	58.1	+3.3	3/2	1.00/0.56	[−5.0, +13.1]

4.7. The Correlator Readout Is Not Linearly Redundant

A natural objection to any entangling architecture is that its correlator readout adds nothing the downstream head could not synthesize from the single-qubit marginals. We test this by regressing each $\langle Z_a Z_b \rangle$ linearly on the full set $\{\langle Z_j \rangle\}_{j=1}^{Q}$ over all held-out windows (Table 9). The scope of the test bears stating, because it establishes linear non-recoverability and not separability: a product state satisfies $\langle Z_a Z_b \rangle = \langle Z_a \rangle \langle Z_b \rangle$, a bilinear function that a linear probe generically fails to reproduce as well, so a low R^2 bounds what a linear head can reconstruct rather than certifying that the register is entangled. The ablation of Table 7 carries the complementary evidence that the correlators are used.

Table 9. The mean coefficient of determination when each edge correlator $\langle Z_a Z_b \rangle$ is linearly regressed on the full set of single-qubit expectations, over all held-out windows—low values indicate that the two-qubit readout is not linearly recoverable from the single-qubit marginals.

**Dataset**	**Modality**	**Edges**	**Mean R^2**
WESAD	HRV	10	0.057
WESAD	EDA	6	0.020
SWELL	HRV	10	0.022
SWELL	EDA	10	0.014

Between 1.4% and 5.7% of correlator variance is linearly recoverable on average across all four dataset–modality combinations, and no edge exceeds 0.08 (Supplementary Section S7), so the two-qubit readout supplies information that no linear function of the single-qubit marginals reproduces. Taken with the ablation, where removing that readout costs 5.0 macro-F1 on WESAD and 3.0 on SWELL, the entangled component is both non-redundant and load-bearing—the clearest sense in which the encoder contributes, and one the matched twin cannot reproduce by construction.

4.8. Capacity Is Set by Physiology, Not by Qubit Count

Figure 8 sweeps the qubit budget over $Q \in \{4, 5, 6, 8\}$ with all else fixed. Honest fused macro-F1 is 83.71, 86.18, 86.18, and 86.18 on WESAD and 61.77, 61.42, 61.42, and 61.42 on SWELL-KW for the first seed. The equality from $Q = 5$ onward is not an empirical

https://doi.org/10.3390/s1010000

coincidence that a second seed could overturn: the register is built from the branches present in the feature set and truncated to the budget, so a budget of 6 or 8 realizes exactly the same five-branch (four-branch for WESAD EDA) circuit, with the same parameters, initialization, and data path, as a budget of 5 (Supplementary Table S13 lists the realized register for every budget). The only empirical comparison in the figure is $Q = 4$ against $Q = 5$, and we have repeated it across the three seeds with each seed fused separately: fused macro-F1 at $Q = 4$ is 79.9 ± 3.4 on WESAD and 58.0 ± 3.4 on SWELL-KW, against 81.6 ± 4.8 and 64.8 ± 4.5 at $Q = 5$ (per-seed values 83.7/77.1/78.7 and 61.8/55.1/57.3 against 86.2/82.0/76.6 and 61.4/70.0/63.1); at the window level the paired difference over 15 fold $\times$ seed pairs is within noise in every cell ($p_{\text{Holm}} = 1.0$ on WESAD HRV, SWELL-KW HRV, and SWELL-KW EDA), and exactly zero on WESAD EDA, whose four branches leave both budgets identical—the sanity check that the identity argument predicts. At $Q = 5$ the seed-42 fused values are the highest of the three on WESAD and the lowest on SWELL-KW; at $Q = 4$ they are the highest on both.

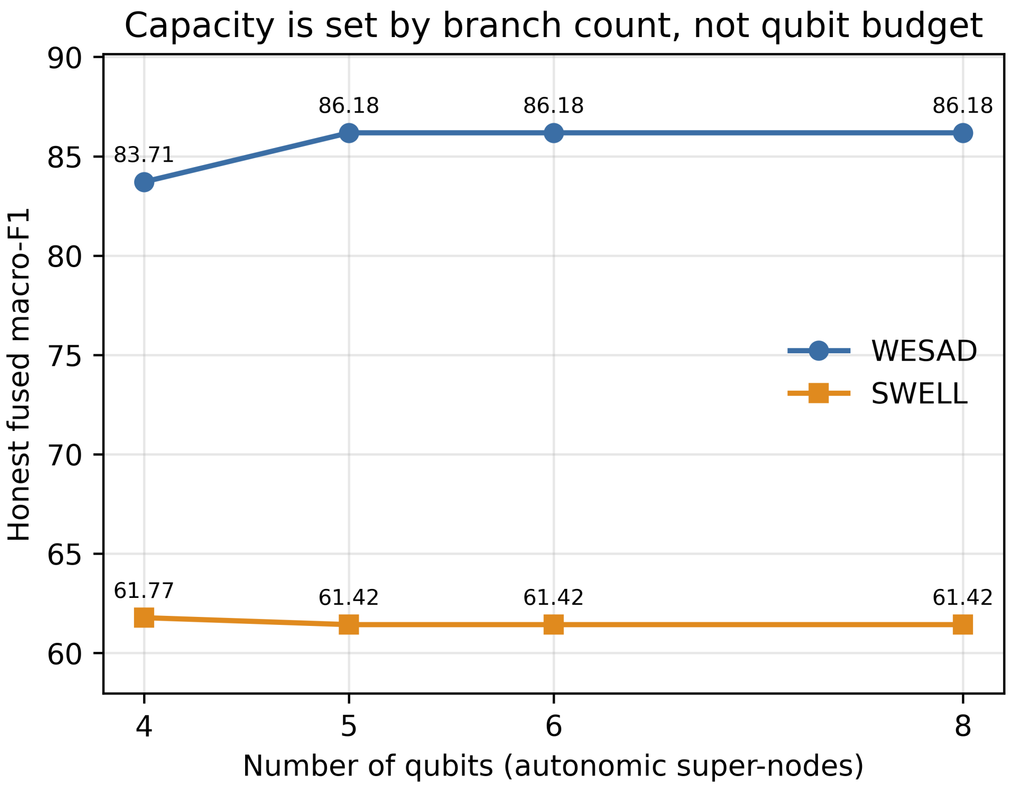

Figure 8. Honest fused macro-F1 versus the number of qubits (autonomic super-nodes). Performance is identical at $Q = 6$ and $Q = 8$ by construction: budgets above the occupied branch count realize the same register as $Q = 5$ (Supplementary Table S13). The only empirical comparison is $Q = 4$ against $Q = 5$; the figure plots a single seed (seed 42), and the three-seed comparison is given in the text and Table S16 of Supplementary Section S14.

Reducing the register to $Q = 4$ forces the last branch to merge into the one before it. For the first seed this costs 2.47 macro-F1 on WESAD and moves SWELL-KW by $+0.35$ in favor of $Q = 4$; over three seeds the fused difference favors $Q = 5$ by 1.75 points on WESAD and 6.8 on SWELL-KW, with no window-level cell significant. From $Q = 5$ on the results are identical at $Q = 6$ and $Q = 8$ by construction, because the register is built from the occupied branches alone; capacity is therefore set by the autonomic branch count rather than the qubit budget, though the sweep on its own does not establish the six-qubit ceiling, which rests on the branch count and the barren-plateau argument of Section 3.3.4.

4.9. Learned Autonomic Couplings

Each super-node maps to a qubit, so the trained entangler magnitudes κ_{ab} are directly inspectable (Figure 9). They lie between 0.08 and 0.18. The tonic branch carries the strongest mean coupling on SWELL-KW EDA (0.155 against 0.121–0.146) and ties with the phasic branch on WESAD EDA (0.150 against 0.147): a node-level summary of per-edge values whose ordering, as discussed next, does not reproduce across seeds. One cross-panel regularity is firmer than any single ranking: electrodermal couplings run stronger than heart-rate-variability couplings by factors of 1.30 and 1.34 (pooled Mann–Whitney $p = 3.2 \times 10^{-5}$, one-sided, over the 16 electrodermal and 20 heart-rate-variability edge means). This is a distributional statement, not dependent on the per-edge ordering. Per-edge ranks and dispersions are in Supplementary Section S8.

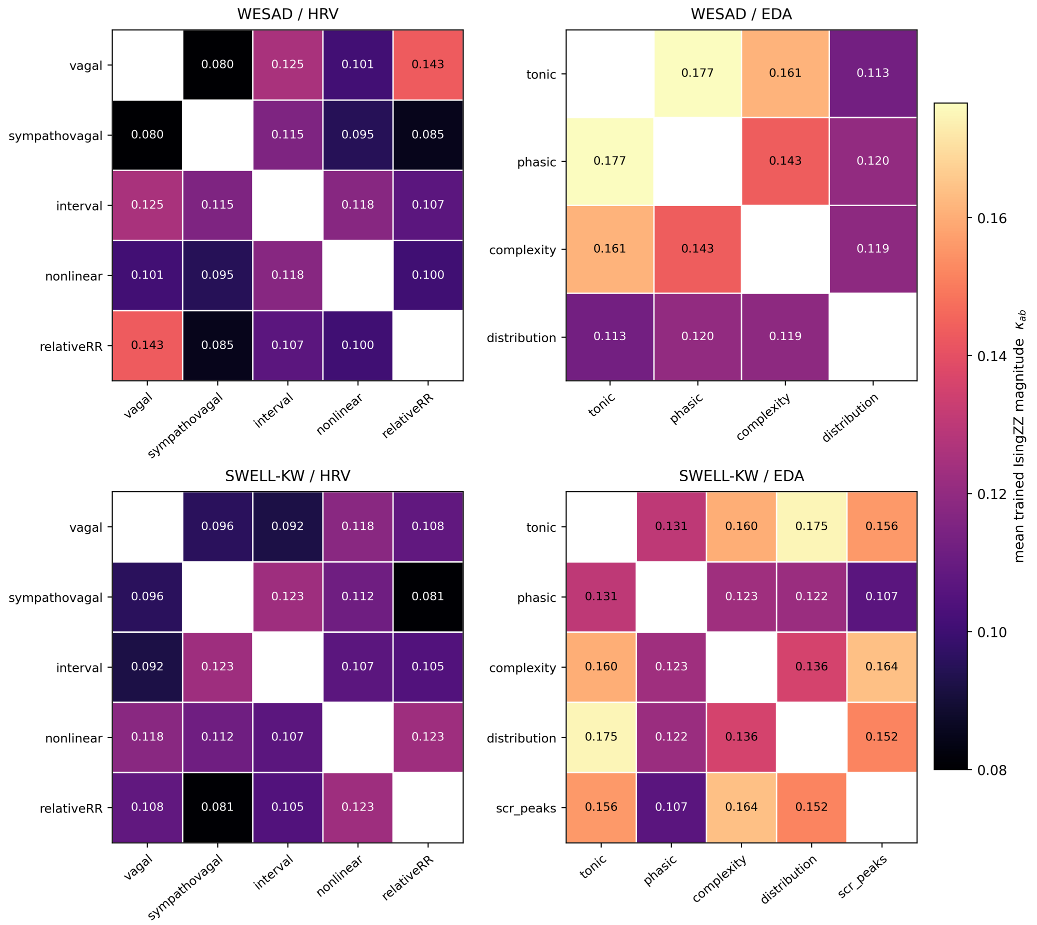

Figure 9. Learned inter-branch quantum couplings: the mean trained IsingZZ magnitude κ_{ab} across seeds and folds, diagonal masked. The four panels have been plotted with the same color scale (0.080–0.177)—so that intensities can be directly compared between panels—and each cell is labeled with its value. WESAD EDA realizes four autonomic branches, and the other three cells five.

A negative finding belongs alongside it: cross-seed rank agreement is weak, with mean pairwise Spearman correlation between per-seed coupling vectors being −0.04, 0.35, 0.05, and −0.02 with per-edge coefficients of variation of 0.30–0.91. The per-edge ordering does not reproduce across initializations, so we present it as exploratory rather than as a validated physiological readout.

4.10. Robustness to Simulated Device Noise

Table 10 retrains and evaluates the model on a density-matrix simulator with a depolarizing channel of probability $p = 0.01$ after every layer, so that noise acts during training and

inference. The experiment uses a reduced epoch budget to keep density-matrix simulation tractable, so its noiseless baseline differs from Table 2 (fold-mean ECE 11.2 against 11.9 on HRV, 19.2 against 15.6 on EDA); the comparison of interest is the within-row noiseless-versus-noisy delta. The table now reports all three seeds (per-seed values in Supplementary Table S15 of Section S14): the largest $|\Delta|$ in macro-F1 over the three seeds is 0.84 (HRV) and 0.67 (EDA), the largest $|\Delta|$ in ECE is 1.95, and the ECE delta is negative in all three seeds on HRV while it has no consistent sign on EDA, so the conclusion below does not rest on one initialization.

Table 10. Robustness under a simulated depolarizing channel ($p = 0.01$), WESAD, honest protocol, three seeds. Features, graphs, depth, and schedule are identical, and each model is trained under the device it is evaluated on. Noiseless and noisy columns are mean ± SD over seeds; Δ is noisy minus noiseless, given as mean ± SD with the range over the three seeds. The reduced epoch budget of this experiment makes its noiseless baseline differ from Table 2 (fold-mean ECE 11.2 against 11.9 on HRV, 19.2 against 15.6 on EDA); the within-row delta is the quantity of interest.

Modality	Metric	Noiseless	Noisy	Δ (Mean ± SD; Range)
HRV	ECE	11.24 ± 1.05	10.95 ± 0.83	-0.28 ± 0.27 $[-0.56, -0.01]$
HRV	NLL	0.761 ± 0.020	0.759 ± 0.021	-0.002 ± 0.004 $[-0.006, +0.002]$
HRV	Brier	0.449 ± 0.005	0.449 ± 0.004	-0.001 ± 0.003 $[-0.004, +0.001]$
HRV	Macro-F1	62.21 ± 1.76	62.74 ± 1.76	$+0.52 \pm 0.49$ $[-0.05, +0.84]$
HRV	Accuracy	65.81 ± 2.41	65.99 ± 1.85	$+0.18 \pm 0.57$ $[-0.28, +0.82]$
EDA	ECE	19.25 ± 2.37	19.72 ± 1.03	$+0.47 \pm 1.35$ $[-0.69, +1.95]$
EDA	NLL	1.170 ± 0.340	1.171 ± 0.342	0.000 ± 0.019 $[-0.018, +0.021]$
EDA	Brier	0.421 ± 0.023	0.424 ± 0.021	$+0.003 \pm 0.011$ $[-0.007, +0.015]$
EDA	Macro-F1	67.79 ± 1.00	67.49 ± 0.81	-0.31 ± 0.35 $[-0.67, +0.03]$
EDA	Accuracy	75.72 ± 0.82	75.59 ± 0.87	-0.14 ± 0.33 $[-0.52, +0.09]$

Calibration changes by at most 1.95 ECE points in any seed and modality: a consistent slight improvement on HRV (-0.28 ± 0.27) and no consistent direction on EDA ($+0.47 \pm 1.35$). Discrimination by less than one macro-F1 point, so the reported behavior is not an artifact of noiseless statevector simulation, the principal caveat attaching to simulator-only quantum machine learning. Depolarizing noise damps expectation values towards zero and so reduces confidence, which makes a small ECE reduction unsurprising where it occurs; the result indicates robustness rather than benefit. The same holds for measurement statistics: resampling the trained readout under the exact $N = 1024$ shot law over ten repeats moves the mean correlator R^2 by less than 0.001 in every cell, from analytic baselines of 0.110, 0.041, 0.018, and 0.034. Those baselines are formed by the shot-noise analysis on its own readout sample and are not the fold-averaged values of Table 9; what this experiment bounds is the size of the shot-induced change, not the level. Because all readout operators are diagonal in the computational basis, their shot errors would be correlated on hardware; treating them independently is a first-order approximation.

4.11. Portability of the Protocol Finding to a Third Corpus

A protocol claim carries weight only if it survives outside the corpora that motivated it. Table 11 drives the AffectiveROAD front end of Section 3.8 through the same three partitions, and Figure 10 places the corpus beside the two release corpora.

Two things stand out, and they are the two claims of this paper. Honest performance is at chance per window: every model scores 25.8–35.5 macro-F1 on a three-class problem whose chance level is about 33, with one-vs-rest AUC between 55.0 and 58.0, and session-level fusion reaches 60.4 on 27 sessions with a 95% percentile bootstrap interval of ±17.8, recovering the nine low-stress sessions (per-class F1 85.7) and confusing the two stress

bands with each other (52.6 and 42.9). The fusion unit is the same construction as on the release corpora; windows grouped within a subject and class, but on those corpora that unit is an experimental block, whereas here the band is assigned per window from a continuous metric, so the level of the fused score is not comparable across corpora; the inflation of that score under the two leaky protocols is. The moment subjects or near-duplicate windows are allowed to straddle the split, the identical models inflate: by +7.2 to +54.2 macro-F1 points for the encoder and the random forest under the subject-dependent split and by +18.7 to +62.5 under window overlap. The tree ensembles reach 90–98 from chance under the overlap split, and the encoder inflates the least of the three models there. The gradient-boosting baseline is the one exception worth naming: under the honest and the subject-dependent protocols, it collapses to the majority class (balanced accuracy 33%) and therefore registers its inflation only under the overlap leak, +64.2 and +66.6; we report it as measured. Calibration behaves as Section 4.3 describes: with the same calibrator, temperature scaling fitted on a subject-sharing split leaves the gradient-boosting expected calibration error at 21.0 (HRV) and 29.9 (EDA), and fitted subject-disjointly at 6.7 and 5.0.

Table 11. Evaluation-inflation gap on the AffectiveROAD portability check, macro-F1 points, five-fold regime. Honest = subject-grouped five-fold, three seeds (mean over seeds and folds); the leakage-inducing protocols run one seed, as in Table 4. Δ = leaky minus honest. Chance macro-F1 for three classes is about 33, so the honest column is at chance for every model and the leaky columns are manufactured. Fused row in bold; RF = random forest, GBM = histogram gradient-boosting machine. Supplementary Section S15 adds the LOSO regime and the four-variant calibration-split experiment, and documents two alternative labeling rules that we did not run. The fused row aggregates windows within a driver × band; unlike the subject × condition sessions of the release corpora, that unit is defined by the label, so its level is not a decision-level estimate.

Modality	Honest	Subject-Dep.	Δ	Window-Overlap	Δ
HRV	32.8	40.0	+7.2	51.5	+18.7
HRV (RF)	35.5	66.6	+31.1	97.9	+62.5
HRV (GBM)	25.8	27.2	+1.4	92.5	+66.6
EDA	31.8	45.7	+13.9	51.4	+19.7
EDA (RF)	34.0	88.3	+54.2	93.7	+59.7
EDA (GBM)	25.8	26.0	+0.2	90.0	+64.2
**Fused**	**60.4**	**62.2**	**+1.8**	**81.0**	**+20.6**

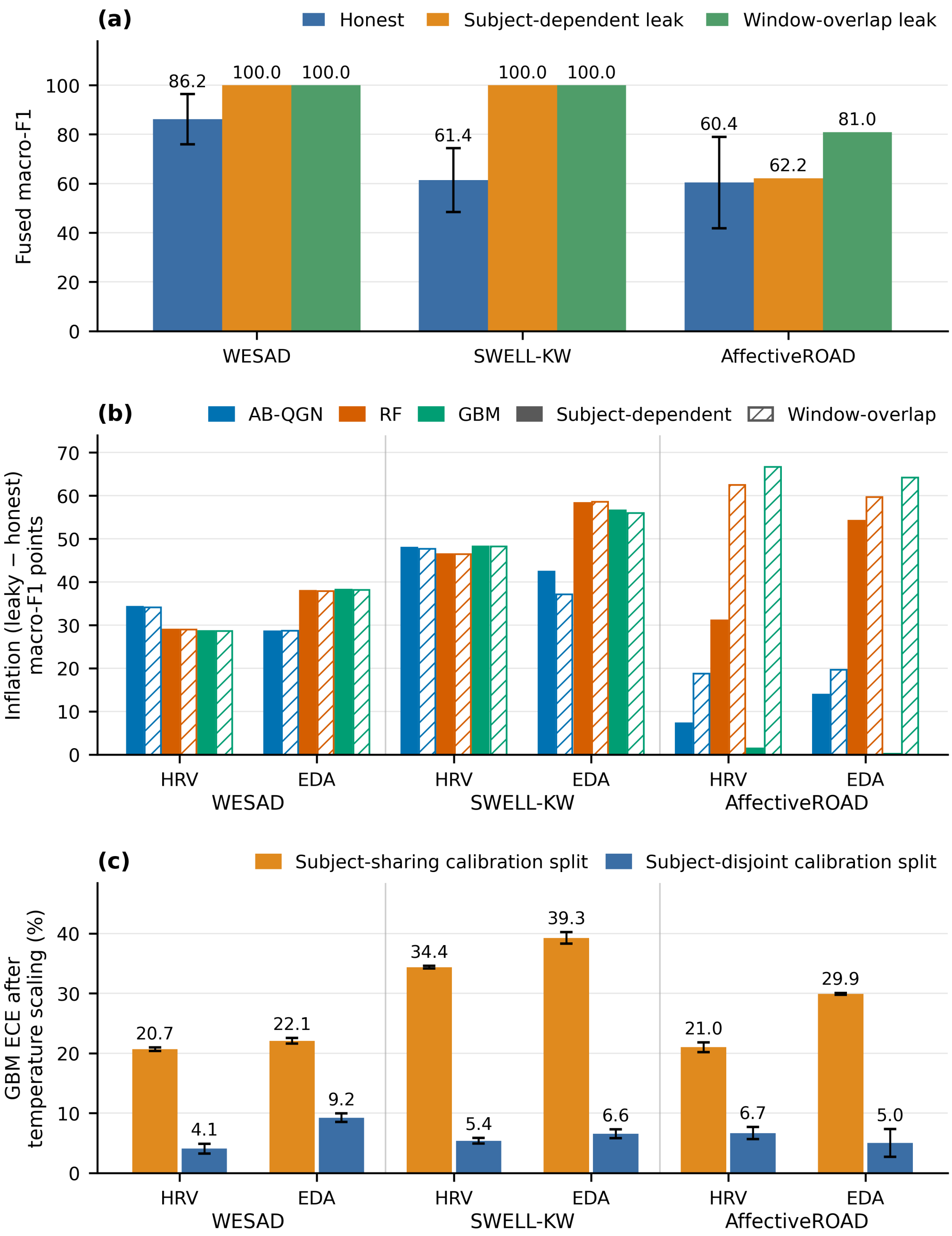

Figure 10. The protocol decides the number on all three corpora. (**a**) Fused macro-F1 of the identical AB-QGN under the honest, subject-dependent, and window-overlap protocols; error bars are ±1.96 session-level bootstrap standard errors on the honest score. (**b**) Per-modality inflation (leaky minus honest) for AB-QGN, random forest (RF), and gradient boosting (GBM). Color identifies the model (AB-QGN, blue; RF, vermilion; GBM, green); within each model, the solid bar is the subject-dependent split and the hatched bar the window-overlap split; exact values are given in Tables 4 and 11 and Supplementary Table S9. (**c**) Calibration leak for the gradient-boosting baseline with the same calibrator: expected calibration error after temperature scaling fitted on a subject-sharing split (orange) against a subject-disjoint split (blue); error bars are ±1 standard deviation over three seeds. Both bars use temperature scaling, so the subject-sharing values differ from the isotonic, conventionally calibrated values of Table 5. The honest protocol is five-fold over three seeds; leakage-inducing protocols use one seed. For AffectiveROAD, panel (**a**) uses the driver × band unit of Section 3.8, which is defined by the label rather than by an experimental block.

What this does and does not establish should be stated plainly. It establishes that the protocol effect is a property of the partition rather than of the two release corpora or of their

shared feature definition: it appears on a corpus with different sensors, a different setting, a different label construction, and a front end of our own, and it appears for every model under at least one of the two leaky protocols. It establishes nothing about the encoder, whose window-level discrimination is at chance here for every model, and whose 27 fused sessions cannot separate two models; no encoder claim in this paper rests on this corpus. Raising the honest scores may require a continuous beat series, a five-minute window, the drive annotations, and the raw electrodermal signal rather than a different classifier, which is the external validation named in Section 5.3.

5. Discussion

5.1. Principal Findings

Evaluation leakage acts twice. It inflates accuracy by 27–61 macro-F1 points—tree ensembles inflating most and the fused AB-QGN least—and it corrupts calibration through the calibrator's own held-out split, moving expected calibration error up to six-fold and the fitted temperature by two orders of magnitude. The second is more insidious because the calibrator's validation set endorses it. Both survive strict LOSO, and both reappear on a third corpus with a front end of our own, where the honest protocol shows there is no accuracy to be had and the window-overlap protocol manufactures 90–98 macro-F1 for the tree ensembles (Section 4.11).

5.2. Why This Architecture

The design targets deployment, not leaderboard position, and four results bear on what a monitor for unseen individuals needs. It depends least of any configuration on subject identity, inflating 13.8 macro-F1 points against 28.7–58.6 for the baselines. It degrades gracefully as the cohort shrinks, 0.35 macro-F1 from the better baseline at three training subjects and leading in two of four cells. This is the regime this field operates in, though that experiment runs the encoder without the subject-adversarial branch (Section 4.4). It does so from 54–75 variational-circuit parameters ($L[3Q + \binom{Q}{2}]$ at the realized $Q = 4$–5 and $L = 3$) and a 10–15 dimensional readout, against 300 trees and 400 boosting iterations. And it is auditable by construction rather than by attribution: each qubit is an autonomic branch, so couplings are read off the trained parameters, which is how the tonic branch's leading coupling on SWELL-KW EDA and the 1.30–1.34 electrodermal-over-HRV ratio were obtained. A tree ensemble cannot supply this; a deep network only through attribution that need not reflect the mechanism. What the quantum component adds is measurable against the only comparison that isolates it. The structure-matched classical twin (identical graph, features, depth, schedule, and calibration split, differing only in the encoder, and carrying 7201 encoder parameters against the circuit's 75) reaches 51.99 against 61.42 fused macro-F1 on SWELL-KW and 83.71 against 86.18 on WESAD, so the fused margins there are 9.4 and 2.5 points, and the parameter-matched twin with 73 encoder parameters reaches 58.1 and 81.1. Tested to the standard of the calibration comparison (Table 8), the WESAD margins are one and two sessions and not significant, and the SWELL-KW margin over the structure-matched twin, eight discordant sessions, is not significant by McNemar ($p = 0.29$) or paired permutation ($p = 0.22$), with a paired bootstrap interval of $[-4.1, +22.5]$; the window-level differences favor neither encoder. Across the three seeds, AB-QGN stays ahead of both twins on SWELL-KW (+3.3 to +12.1 points) but not on WESAD, where the parameter-matched twin ties it; the direction on SWELL-KW is the same at every seed, but the session counts do not license a stronger statement, and we make none. Consistently, the representation is not linearly redundant and is load-bearing: its correlators are not linearly recoverable from single-qubit expectations (mean $R^2 \leq 0.06$), and capacity follows the branch count rather than the qubit budget. The ablation isolates which part carries that

load. Every variant removing entangling structure is negative at the fusion level on both corpora: the entanglers (-5.0 and -3.1), the correlator readout (-5.0 and -3.0), and the structure-matched encoder entire (-2.5 and -9.4). By contrast, data re-uploading and the mRMR filter each improve SWELL-KW when removed. The consistency is therefore specific to the entangling machinery rather than general to the quantum components, and that narrower claim is the one the ablation supports.

5.3. Limitations

AB-QGN improves neither discrimination nor calibration over fixed-configuration tree ensembles and is behind random forest on WESAD HRV; its advantage is over the structure-matched and parameter-matched classical encoders, not over a model of a different family, and that advantage is directional rather than statistically established at the available session counts (Section 4.6). SWELL-KW EDA approaches chance (honest macro-F1 $\approx$ 35–37, AUC $\approx$ 55–57). Minority classes remain the hardest: per-class F1 falls to 18–38 at single-modality level, and although fusion recovers WESAD amusement to 76.9, SWELL time-pressure stays at 34.8, partly structural, since amusement lasts little longer than the five-minute analysis window, which is why the feature release's authors excluded it [47]. The calibration-leakage result covers one model family and one pair of calibration methods; the effect is large, mechanistically clear, and consistent across three seeds, but its generality across estimators is untested. Learned per-edge couplings are not initialization-stable, and we therefore treated seed sensitivity as a live risk for the two analyses that were first run on one seed: the qubit-budget sweep and the noise re-evaluation. Both have now been repeated across the three seeds (Sections 4.8 and 4.10); the sweep's saturation from $Q = 5$ is structural and seed-independent by construction, and the noise deltas stay below two ECE points and one macro-F1 point in every seed, so the seed instability of Section 4.9 is confined to the ordering of individual couplings and does not propagate to those conclusions. The main fused scores remain single-seed session-level figures (seed 42) whose across-seed spread is reported in Section 4.8. The noise analysis covers one depolarizing probability on one dataset, and the shot analysis treats readout operators independently, where hardware would correlate them; neither substitutes for hardware execution. Sex and gender are known only at cohort level (12 of 15 and 17 of 25 participants male, mean ages 27.5 and 25 years; Table 1) and were not recorded in the released tables, so the cohort sizes preclude a stratified analysis, which we declare as a limitation on generalizability. Both datasets are laboratory-collected with 15 and 22 subjects, and whether laboratory stressors represent clinically relevant anxiety is unresolved [77]: the preventive application motivates this work, but nothing here shows the model detects incipient disorder. Two corpora are also the floor, not the ceiling, for a protocol claim. We note in mitigation that the two findings this paper rests on (inflation under subject-sharing and window-overlapping splits, and calibration leakage through the calibrator's own split) are measured here on every one of fourteen model–modality configurations in two regimes, that the first of them has been reproduced independently by the authors of both corpora on their own data [32,47] and by other groups on other corpora and signals [12,76], and on WESAD itself [15] and that both reappear on the third corpus of Section 4.11. That corpus enters on different terms from the other two, and the difference bounds what it shows. Its features come from a front end of our own rather than from an independent release, its classes are bands of a continuous observer rating rather than an experimental condition, and its honest window-level performance is at chance for every model, so it can carry the protocol claim but not a claim about any model. External validation on a further, ideally ambulatory, cohort therefore remains the priority next step, and the released loader accepts any feature table with the documented columns, so the protocol transfers unchanged.

5.4. Implications

At the realized register sizes ($Q = 4$–5) and $L = 3$, the encoder adds only 10–15 readout features and 54–75 variational-circuit parameters, and session-level fusion issues one decision per session rather than per window. We make no on-device timing claim, and a deployed monitor would sit inside a wearable sensing platform whose privacy and integrity constraints are a separate design problem [78]. The methodological implication generalizes beyond this architecture—both differences we report are cheap to compute and belong in standard practice, quantum model or not.

6. Conclusions

We introduced AB-QGN, a hybrid quantum–classical model encoding autonomic regulatory branches onto the qubits of a shallow entangling circuit, and used it as a fixed instrument to measure how far reported performance in wearable stress detection depends on the evaluation protocol. Accuracy inflates by 27–61 macro-F1 points under leakage; honest fused macro-F1 is 86.2 (WESAD) and 61.4 (SWELL-KW), stable under leave-one-subject-out at 84.0 and 55.1 and above all 10,000 label permutations. On a third corpus carried through the identical protocols with a front end of our own, honest performance is at chance per window, while window-overlap leakage manufactures 90–98 macro-F1 for tree ensembles, so the effect belongs to the partition rather than to a corpus or a feature release. Leakage also acts on confidence: a calibrator fitted on a split sharing subjects with training selects a temperature two orders of magnitude from the subject-disjoint value and leaves expected calibration error up to six times higher, a failure its own validation set endorses. The architecture generalizes to unseen individuals with the least dependence on subject identity of any configuration tested on the two release corpora, reaches parity with far larger classical models in the three-subject regime of the cohort-size experiment, and is auditable by construction because each qubit is an autonomic branch—its correlator readout is not linearly redundant, load-bearing at the fusion level, and robust to finite shots and simulated noise. We claim no accuracy or calibration advantage. Nothing in the measurement is specific to a quantum encoder, and we offer it as a reporting standard: fix one architecture, drive it through one leakage-free and two leakage-inducing splits, group the calibration split as carefully as the training split, and report the differences.

Supplementary Materials: The following supporting information can be downloaded at https://www.mdpi.com/article/10.3390/s1010000/s1: Section S1: Architecture comparison with prior stress/affect detection models, with Table S1: Architecture comparison of representative classical, quantum, and hybrid stress/affect-detection models; Section S2: Method and component comparison, with Table S2: Method and component comparison; Section S3: Expressivity of data re-uploading; Section S4: Trainability, gradients and optimization schedules; Section S5: Comparison with prior work under explicit evaluation protocols, with Table S3: Comparison with prior work on WESAD and SWELL-KW; Section S6: Data efficiency, with Table S4: Data-efficiency curve (macro-F1, mean over three seeds), and Figure S1: Macro-F1 versus number of training subjects k; Section S7: Per-edge correlator independence, with Table S5: Per-edge correlator independence; Section S8: Full learned-coupling statistics, with Table S6: Full learned-coupling statistics; Section S9: Calibration leakage: full four-variant comparison, with Table S7: Gradient boosting under four calibration schemes, honest five-fold protocol, window level, and pooled over held-out windows, and Table S8: Fitted temperature by calibration split, across all 15 folds per cell (three seeds $\times$ five folds); Section S10: Reproducibility; Section S11: Complete evaluation-inflation tables, with Table S9: Evaluation-inflation gap in macro-F1 points, five-fold regime, all fourteen configurations, and Table S10: Evaluation-inflation gap in macro-F1 points, LOSO regime, all fourteen configurations; Section S12: Candidate corpora and selection criteria, with Table S11: Candidate public corpora for wearable stress detection against the five selection criteria; Section S13: Capacity of the classical twins and the realized

register, with Table S12: Parameter accounting of the encoders and full models (subject discriminator excluded), and Table S13: Register realized by each qubit budget; Section S14: Baseline configuration, early-stopping sensitivity and multi-seed robustness, with Table S14: Gradient-boosting macro-F1 under three early-stopping rules, honest five-fold protocol, uncalibrated base model, mean $\pm$ SD over three seeds $\times$ five folds, with the mean number of boosting iterations in parentheses, Table S15: Per-seed noiseless versus depolarizing ($p = 0.01$) results, WESAD, honest protocol, and Table S16: Honest fused macro-F1 at $Q = 4$ and $Q = 5$ per seed, with the window-level paired comparison over 15 fold $\times$ seed pairs; Section S15: Portability check on AffectiveROAD: full detail. References [5–7,9–13,15,17,18,20,21,23,24,26–35,37,41–44,46–61,65,66,68,72,75,78] are cited in the Supplementary Materials.

Author Contributions: Conceptualization, S.P. and S.N.; methodology, S.P. and S.N.; software, S.P.; validation, S.P.; formal analysis, S.P.; investigation, S.P.; resources, S.N.; data curation, S.P.; writing—original draft preparation, S.P.; writing—review and editing, S.P. and S.N.; visualization, S.P.; supervision, S.N.; project administration, S.N. All authors have read and agreed to the published version of the manuscript.

Funding: This research received no external funding.

Institutional Review Board Statement: Ethical review and approval were waived for this study. The work is a secondary computational analysis of publicly available benchmark datasets: WESAD [30] and SWELL-KW [31,32], both fully de-identified and both obtained here as a derived feature release distributed under a CC0 1.0 Public Domain Dedication [46,47], and, for the portability check of Section 4.11, the AffectiveROAD release [48], distributed by its investigators for research use and likewise carrying no identifying information, from which we computed feature windows without altering the recordings. No participant was recruited or contacted. The authors carried out no intervention and took no measurement of their own, handled no human biological material, and at no stage held identifiable personal data; the released tables carry only numeric physiological features, indexed by an arbitrary subject code, and the feature windows we computed from the AffectiveROAD recordings inherit that anonymity. Anonymous data of this kind fall outside the material scope of Regulation (EU) 2016/679 (Recital 26), and no national or Union legislation makes ethical review a condition for the secondary analysis of anonymous data already in the public domain. As for the source collections, the WESAD study was approved by the workers' council and the data security officer of the research center at which it was conducted [30], whereas the original SWELL-KW publication documents the consent procedure but reports no ethics committee and no approval number [31]. The AffectiveROAD investigators describe a real-road driving protocol carried out with volunteer drivers and release the database for research use; their publication likewise reports no ethics committee and no approval number [48].

Informed Consent Statement: Informed consent was obtained from all participants by the original investigators. WESAD participants read and signed a consent form before taking part [30]. In SWELL-KW, the experiment and the recordings were explained to participants in advance, every participant signed a consent form agreeing that the recorded data could be used for research purposes, and a debriefing followed the experiment [31]. In AffectiveROAD, each driver consented to participate voluntarily and to the recording of their physiological, video, and audio data throughout the experiment [48]. For the present secondary analysis, no participant was recruited, and no individual can be identified from any result reported here.

Data Availability Statement: The data presented in this study are derived from resources available in the public domain. The heart-rate-variability and electrodermal feature windows analyzed here are openly available in the "Biometrics for stress monitoring" feature dataset on Kaggle at https://www.kaggle.com/datasets/qiriro/stress (accessed on 28 July 2026) [46], released under a CC0 1.0 Public Domain Dedication, and were derived [47] from WESAD [30] and SWELL-KW [31,32]. The AffectiveROAD recordings analyzed in Section 4.11 are distributed by their investigators through the MIT Media Lab Affective Computing group at https://www.media.mit.edu/groups/affective-computing/data/ (accessed on 2 September 2026), which grants permission to use them for research

purposes [48]; we redistribute no part of that release. No new measurements were taken in this study, and the only data created here are derived: heart-rate variability and electrodermal feature windows computed from those recordings on a five-second grid. Both window tables were produced in an interactive session rather than by a single reusable script, so we archive the resulting tables rather than that code, together with the conversion utility that maps the electrodermal columns onto the release column names and assigns the class labels, the provenance record of the run, the feature list and the label thresholds, so that every result reported here regenerates from the archived tables. The archived tables carry the window anchor, the beat count and the driver and drive identifiers, so the window construction described in Section 3.8 can be checked against them directly. The AB-QGN implementation, the serialized run configurations, the per-fold result tables, and the scripts that rebuild every figure and table are openly archived in Zenodo at https://doi.org/10.5281/zenodo.22789151.

Acknowledgments: The authors thank the creators of the WESAD, SWELL-KW and AffectiveROAD datasets and the maintainers of the publicly released feature set for making these data available.

Conflicts of Interest: The authors declare no conflicts of interest.

Abbreviations

The following abbreviations are used in this manuscript:

AB-QGN	Autonomic-Branch Quantum-Graph Network
AUC	Area under the receiver operating characteristic curve
BalAcc	Balanced accuracy
ECE	Expected calibration error
EDA	Electrodermal activity
GBM	Histogram gradient-boosting machine
HRV	Heart-rate variability
LF/HF	Low frequency/High frequency
LOSO	Leave-one-subject-out
MLP	Multilayer perceptron
mRMR	Minimum redundancy–maximum relevance
NISQ	Noisy intermediate-scale quantum
NLL	Negative log-likelihood
QML	Quantum machine learning
RF	Random forest
SCR	Skin conductance response
SWELL-KW	Smart Reasoning for Well-being at Home and at Work knowledge-work dataset
VLF	Very low frequency
WESAD	Wearable Stress and Affect Detection dataset

References

1. Chojnowska, S.; Ptaszyńska-Sarosiek, I.; Kępka, A.; Knaś, M.; Waszkiewicz, N. Salivary Biomarkers of Stress, Anxiety and Depression. *J. Clin. Med.* **2021**, *10*, 517. https://doi.org/10.3390/jcm10030517.
2. Dhama, K.; Latheef, S.K.; Dadar, M.; Samad, H.A.; Munjal, A.; Khandia, R.; Karthik, K.; Tiwari, R.; Yatoo, M.I.; Bhatt, P.; et al. Biomarkers in Stress Related Diseases/Disorders: Diagnostic, Prognostic, and Therapeutic Values. *Front. Mol. Biosci.* **2019**, *6*, 91. https://doi.org/10.3389/fmolb.2019.00091.
3. Gomes, N.; Pato, M.; Lourenço, A.R.; Datia, N. A Survey on Wearable Sensors for Mental Health Monitoring. *Sensors* **2023**, *23*, 1330. https://doi.org/10.3390/s23031330.
4. Hickey, B.A.; Chalmers, T.; Newton, P.; Lin, C.T.; Sibbritt, D.; McLachlan, C.S.; Clifton-Bligh, R.; Morley, J.; Lal, S. Smart Devices and Wearable Technologies to Detect and Monitor Mental Health Conditions and Stress: A Systematic Review. *Sensors* **2021**, *21*, 3461. https://doi.org/10.3390/s21103461.
5. Ishaque, S.; Khan, N.; Krishnan, S. Trends in Heart-Rate Variability Signal Analysis. *Front. Digit. Health* **2021**, *3*, 639444. https://doi.org/10.3389/fdgth.2021.639444.

6. Nechyporenko, A.; Frohme, M.; Strelchuk, Y.; Omelchenko, V.; Gargin, V.; Ishchenko, L.; Alekseeva, V. Galvanic Skin Response and Photoplethysmography for Stress Recognition Using Machine Learning and Wearable Sensors. *Appl. Sci.* **2024**, *14*, 11997. https://doi.org/10.3390/app142411997.
7. Sriram Kumar, P.; Govarthan, P.K.; Aleem Shaik Gadda, A.; Ganapathy, N.; Fredo Agastinose Ronickom, J. Deep Learning-Based Automated Emotion Recognition Using Multimodal Physiological Signals and Time-Frequency Methods. *IEEE Trans. Instrum. Meas.* **2024**, *73*, 2526912. https://doi.org/10.1109/TIM.2024.3420349.
8. Zhang, Y.; Chen, J.; Tan, J.H.; Chen, Y.; Chen, Y.; Li, D.; Yang, L.; Su, J.; Huang, X.; Che, W. An Investigation of Deep Learning Models for EEG-Based Emotion Recognition. *Front. Neurosci.* **2020**, *14*, 622759. https://doi.org/10.3389/fnins.2020.622759.
9. Xiang, J.Z.; Wang, Q.Y.; Fang, Z.B.; Esquivel, J.A.; Su, Z.X. A multi-modal deep learning approach for stress detection using physiological signals: Integrating time and frequency domain features. *Front. Physiol.* **2025**, *16*, 1584299. https://doi.org/10.3389/fphys.2025.1584299.

10. Gholamiangonabadi, D.; Kiselov, N.; Grolinger, K. Deep Neural Networks for Human Activity Recognition with Wearable Sensors: Leave-One-Subject-Out Cross-Validation for Model Selection. *IEEE Access* **2020**, *8*, 133982–133994. https://doi.org/10.1109/ACCESS.2020.3010715.
11. Vos, G.; Trinh, K.; Sarnyai, Z.; Rahimi Azghadi, M. Generalizable machine learning for stress monitoring from wearable devices: A systematic literature review. *Int. J. Med. Inform.* **2023**, *173*, 105026. https://doi.org/10.1016/j.ijmedinf.2023.105026.
12. Hughes, C.M.L.M.; Zhang, Y. Bursting the bubble: Data leakage and inflated deep learning accuracy in multivariate time-series frailty classification. *Front. Comput. Sci.* **2026**, *8*, 1700489. https://doi.org/10.3389/fcomp.2026.1700489.
13. Ndungi, R.; Blekanov, I.S. Improving time series forecasting by applying the sliding window approach. *Comput. Nanotechnol.* **2025**, *12*, 11–18. https://doi.org/10.33693/2313-223x-2025-12-2-11-18.
14. Albelali, S.; Ahmed, M. Hidden Leaks in Time Series Forecasting: How Data Leakage Affects LSTM Evaluation Across Configurations and Validation Strategies. *arXiv* **2025**, arXiv:2512.06932.
15. Zhu, Z.; Zhou, R. TL-QCHResnet: Quantum transfer learning based on heart rate variability data. *Biomed. Signal Process. Control* **2026**, *123*, 110563. https://doi.org/10.1016/j.bspc.2026.110563.
16. Ahmadi, N.; Abbasian, P.; Hou, Q.; Powell, L.; Hammond, T. Physiological Sensing and Machine Learning Approaches Toward Anxiety Detection: A Systematic Review. *IEEE Sens. Rev.* **2026**, *3*, 89–116. https://doi.org/10.1109/SR.2025.3643386.
17. Rebentrost, P.; Mohseni, M.; Lloyd, S. Quantum Support Vector Machine for Big Data Classification. *Phys. Rev. Lett.* **2014**, *113*, 130503. https://doi.org/10.1103/PhysRevLett.113.130503.
18. Havlíček, V.; Córcoles, A.D.; Temme, K.; Harrow, A.W.; Kandala, A.; Chow, J.M.; Gambetta, J.M. Supervised learning with quantum-enhanced feature spaces. *Nature* **2019**, *567*, 209–212. https://doi.org/10.1038/s41586-019-0980-2.
19. Mittal, M. Quantum Machine Learning: Harnessing Quantum Algorithms for Supervised and Unsupervised Learning. *Int. J. Innov. Res. Sci. Eng. Technol.* **2022**, *11*, 11631–11637. https://doi.org/10.15680/ijirset.2022.1109004.
20. Pérez-Salinas, A.; Cervera-Lierta, A.; Gil-Fuster, E.; Latorre, J.I. Data re-uploading for a universal quantum classifier. *Quantum* **2020**, *4*, 226. https://doi.org/10.22331/q-2020-02-06-226.
21. McClean, J.R.; Boixo, S.; Smelyanskiy, V.N.; Babbush, R.; Neven, H. Barren plateaus in quantum neural network training landscapes. *Nat. Commun.* **2018**, *9*, 4812. https://doi.org/10.1038/s41467-018-07090-4.
22. Preskill, J. Quantum Computing in the NISQ era and beyond. *Quantum* **2018**, *2*, 79. https://doi.org/10.22331/q-2018-08-06-79.
23. Gupta, H.; Varshney, H.; Sharma, T.K.; Pachauri, N.; Verma, O.P. Comparative performance analysis of quantum machine learning with deep learning for diabetes prediction. *Complex Intell. Syst.* **2022**, *8*, 3073–3087. https://doi.org/10.1007/s40747-021-00398-7.
24. Rajamohana, S.P.; Yelamali, V.; Soni, P.; Vanahalli, M.K.; Prasad, P. Hybrid Quantum Graph Neural Network for Brain Tumor MR Image Classification. *J. Sci. Ind. Res. (JSIR)* **2025**, *84*, 60–71. https://doi.org/10.56042/jsir.v84i1.14112.
25. Bali, M.; Mishra, V.P.; Yenkikar, A.; Chikmurge, D. QuantumNet: An enhanced diabetic retinopathy detection model using classical deep learning-quantum transfer learning. *MethodsX* **2025**, *14*, 103185. https://doi.org/10.1016/j.mex.2025.103185.
26. Padha, A.; Sahoo, A. Quantum Enhanced Machine Learning for Unobtrusive Stress Monitoring. In *Proceedings of the 2022 Fourteenth International Conference on Contemporary Computing (IC3-2022)*; Association for Computing Machinery: New York, NY, USA, 2022; pp. 476–483. https://doi.org/10.1145/3549206.3549288.
27. Onim, M.S.H.; Humble, T.S.; Thapliyal, H. Quantum Hybrid Support Vector Machines for Stress Detection in Older Adults. In *Proceedings of the 2025 IEEE International Conference on Consumer Electronics (ICCE)*; IEEE: Piscataway, NJ, USA, 2025; pp. 1–4. https://doi.org/10.1109/ICCE63647.2025.10929873.
28. Padha, A.; Sahoo, A. QCLR: Quantum-LSTM contrastive learning framework for continuous mental health monitoring. *Expert Syst. Appl.* **2024**, *238*, 121921. https://doi.org/10.1016/j.eswa.2023.121921.
29. Rababah, B.; Farooq, B. Quantum Machine Learning in Transportation: A Case Study of Pedestrian Stress Modelling. In *Proceedings of the 2025 IEEE 28th International Conference on Intelligent Transportation Systems (ITSC)*; IEEE: Piscataway, NJ, USA, 2025; pp. 3054–3059. https://doi.org/10.1109/ITSC60802.2025.11423545.
30. Schmidt, P.; Reiss, A.; Duerichen, R.; Marberger, C.; Van Laerhoven, K. Introducing WESAD, a Multimodal Dataset for Wearable Stress and Affect Detection. In *Proceedings of the 20th ACM International Conference on Multimodal Interaction (ICMI '18)*; Association for Computing Machinery: New York, NY, USA, 2018; pp. 400–408. https://doi.org/10.1145/3242969.3242985.
31. Koldijk, S.; Sappelli, M.; Verberne, S.; Neerincx, M.A.; Kraaij, W. The SWELL Knowledge Work Dataset for Stress and User Modeling Research. In *Proceedings of the 16th International Conference on Multimodal Interaction (ICMI '14)*; Association for Computing Machinery: New York, NY, USA, 2014; pp. 291–298. https://doi.org/10.1145/2663204.2663257.
32. Koldijk, S.; Neerincx, M.A.; Kraaij, W. Detecting Work Stress in Offices by Combining Unobtrusive Sensors. *IEEE Trans. Affect. Comput.* **2018**, *9*, 227–239. https://doi.org/10.1109/TAFFC.2016.2610975.
33. Tazarv, A.; Labbaf, S.; Reich, S.M.; Dutt, N.; Rahmani, A.M.; Levorato, M. Personalized Stress Monitoring using Wearable Sensors in Everyday Settings. In *Proceedings of the 2021 43rd Annual International Conference of the IEEE Engineering in Medicine & Biology Society (EMBC)*; IEEE: Piscataway, NJ, USA, 2021; pp. 7332–7335. https://doi.org/10.1109/EMBC46164.2021.9630224.

34. R, R.V.S.S.B.; Abdohwr, R.; M, K.K.; T, M.; Devika, P. Domain-Invariant Representation Learning for Cross-Subject Biomedical Signal Analysis. In *Proceedings of the 2025 International Conference on Intelligent Communication Networks and Computational Techniques (ICICNCT)*; IEEE: Piscataway, NJ, USA, 2025; pp. 1–6. https://doi.org/10.1109/ICICNCT66124.2025.11232887.
35. Ganin, Y.; Ustinova, E.; Ajakan, H.; Germain, P.; Larochelle, H.; Laviolette, F.; Marchand, M.; Lempitsky, V. Domain-Adversarial Training of Neural Networks. In *Domain Adaptation in Computer Vision Applications*; Csurka, G., Ed.; Springer International Publishing: Cham, Switzerland, 2017; pp. 189–209. https://doi.org/10.1007/978-3-319-58347-1_10.
36. Alkurdi, A.; Clore, J.; Sowers, R.; Hsiao-Wecksler, E.T.; Hernandez, M.E. Resilience of Machine Learning Models in Anxiety Detection: Assessing the Impact of Gaussian Noise on Wearable Sensors. *Appl. Sci.* **2025**, *15*, 88. https://doi.org/10.3390/app15010088.
37. Guo, C.; Pleiss, G.; Sun, Y.; Weinberger, K.Q. On Calibration of Modern Neural Networks. In *Proceedings of the 34th International Conference on Machine Learning, Sydney, NSW, Australia, 6–11 August 2017*; Precup, D., Teh, Y.W., Eds.; Proceedings of Machine Learning Research; JMLR: Cambridge, MA, USA, 2017; Volume 70, pp. 1321–1330.
38. Balanya, S.A.; Maroñas, J.; Ramos, D. Adaptive temperature scaling for Robust calibration of deep neural networks. *Neural Comput. Appl.* **2024**, *36*, 8073–8095. https://doi.org/10.1007/s00521-024-09505-4.
39. Fernando, K.R.M.; Tsokos, C.P. Dynamically Weighted Balanced Loss: Class Imbalanced Learning and Confidence Calibration of Deep Neural Networks. *IEEE Trans. Neural Netw. Learn. Syst.* **2022**, *33*, 2940–2951. https://doi.org/10.1109/TNNLS.2020.3047335.
40. Khot, B.A.P.; Mahadevaswamy; Ittannavar, S.S. Comprehensive Comparative Analysis of Variational Quantum Classifier and Quantum Kernel SVM Under NISQ Noise with Classical Baseline and Statistical Significance Evaluation. In *Proceedings of the 2025 2nd International Conference on Software, Systems and Information Technology (SSITCON)*; IEEE: Piscataway, NJ, USA, 2025; pp. 1–5. https://doi.org/10.1109/SSITCON66133.2025.11341965.
41. Mordacci, M.; Ferrari, D.; Amoretti, M. Multi-Class Quantum Convolutional Neural Networks. In *Proceedings of the 2024 Workshop on Quantum Search and Information Retrieval (QUASAR '24)*; Association for Computing Machinery: New York, NY, USA, 2024; pp. 9–16. https://doi.org/10.1145/3660318.3660326.
42. Schuld, M.; Sweke, R.; Meyer, J.J. Effect of data encoding on the expressive power of variational quantum-machine-learning models. *Phys. Rev. A* **2021**, *103*, 032430. https://doi.org/10.1103/PhysRevA.103.032430.
43. Adarsh, V.; Gangadharan, G.R. Mental stress detection from ultra-short heart rate variability using explainable graph convolutional network with network pruning and quantisation. *Mach. Learn.* **2024**, *113*, 5467–5494. https://doi.org/10.1007/s10994-023-06504-9.
44. Liu, C.; Deng, Y.; Wu, Y.; Yang, R.; Wang, Z.; Zhang, L.; Chen, S.; Zhang, T.; Liu, Y.; Ding, Y.; et al. Graph Neural Networks in EEG-Based Emotion Recognition: A Survey. In *Data Science: Foundations and Applications*; Wong, R.C.W., Tong, H., Lu, H., Kwok, J., Salim, F., Song, Y., Yiu, M.L., Eds.; Springer Nature: Singapore, 2027; pp. 581–598. https://doi.org/10.1007/978-981-92-1947-6_46.
45. Papamentzelopoulos, S.; Nikoletseas, S. Optimized Quantum Classifiers for the Prevention of Anxiety Disorders Using Wearable Data. *Appl. Sci.* **2026**, *16*, 6132. https://doi.org/10.3390/app16126132.
46. Nkurikiyeyezu, K. Biometrics for Stress Monitoring. Kaggle Dataset. 2019. Available online: https://www.kaggle.com/datasets/qiriro/stress (accessed on 28 July 2026).
47. Nkurikiyeyezu, K.; Yokokubo, A.; Lopez, G. Effect of Person-specific Biometrics in Improving Generic Stress Predictive Models. *Sens. Mater.* **2020**, *32*, 703–722. https://doi.org/10.18494/SAM.2020.2650.
48. Haouij, N.E.; Poggi, J.M.; Sevestre-Ghalila, S.; Ghozi, R.; Jaïdane, M. AffectiveROAD system and database to assess driver's attention. In *Proceedings of the 33rd Annual ACM Symposium on Applied Computing (SAC '18)*; Association for Computing Machinery: New York, NY, USA, 2018; pp. 800–803. https://doi.org/10.1145/3167132.3167395.
49. Healey, J.A.; Picard, R.W. Detecting Stress During Real-World Driving Tasks Using Physiological Sensors. *IEEE Trans. Intell. Transp. Syst.* **2005**, *6*, 156–166. https://doi.org/10.1109/TITS.2005.848368.
50. Birjandtalab, J.; Cogan, D.; Pouyan, M.B.; Nourani, M. A Non-EEG Biosignals Dataset for Assessment and Visualization of Neurological Status. In *Proceedings of the 2016 IEEE International Workshop on Signal Processing Systems (SiPS)*; IEEE: Piscataway, NJ, USA, 2016; pp. 110–114. https://doi.org/10.1109/SiPS.2016.27.
51. Hosseini, S.; Gottumukkala, R.; Katragadda, S.; Bhupatiraju, R.T.; Ashkar, Z.; Borst, C.W.; Cochran, K. A Multimodal Sensor Dataset for Continuous Stress Detection of Nurses in a Hospital. *Sci. Data* **2022**, *9*, 255. https://doi.org/10.1038/s41597-022-01361-y.
52. Sabour, R.M.; Benezeth, Y.; De Oliveira, P.; Chappé, J.; Yang, F. UBFC-Phys: A Multimodal Database For Psychophysiological Studies of Social Stress. *IEEE Trans. Affect. Comput.* **2023**, *14*, 622–636. https://doi.org/10.1109/TAFFC.2021.3056960.
53. Chaptoukaev, H.; Strizhkova, V.; Panariello, M.; Dalpaos, B.; Reka, A.; Manera, V.; Thümmler, S.; Ismailova, E.; W., N.; Bremond, F.; et al. StressID: A Multimodal Dataset for Stress Identification. In *Proceedings of the Advances in Neural Information Processing Systems 36 (NeurIPS 2023)*; Neural Information Processing Systems Foundation, Inc. (NeurIPS): San Diego, CA, USA, 2023; pp. 29798–29811. https://doi.org/10.52202/075280-1297.

54. Markova, V.; Ganchev, T.; Kalinkov, K. CLAS: A Database for Cognitive Load, Affect and Stress Recognition. In *Proceedings of the 2019 International Conference on Biomedical Innovations and Applications (BIA)*; IEEE: Piscataway, NJ, USA, 2019; pp. 1–4. https://doi.org/10.1109/BIA48344.2019.8967457.
55. Beh, W.K.; Wu, Y.H.; Wu, A.Y. MAUS: A Dataset for Mental Workload Assessment on N-Back Task Using Wearable Sensor. *arXiv* **2021**, arXiv:2111.02561.
56. Iqbal, T.; Simpkin, A.J.; Roshan, D.; Glynn, N.; Killilea, J.; Walsh, J.; Molloy, G.; Ganly, S.; Ryman, H.; Coen, E.; et al. Stress Monitoring Using Wearable Sensors: A Pilot Study and Stress-Predict Dataset. *Sensors* **2022**, *22*, 8135. https://doi.org/10.3390/s22218135.
57. Amin, M.R.; Wickramasuriya, D.; Faghih, R.T. A Wearable Exam Stress Dataset for Predicting Cognitive Performance in Real-World Settings (Version 1.0.0), 2022. PhysioNet. Available online: https://physionet.org/content/wearable-exam-stress/1.0.0/ (accessed on 3 August 2026).
58. Miranda-Correa, J.A.; Abadi, M.K.; Sebe, N.; Patras, I. AMIGOS: A Dataset for Affect, Personality and Mood Research on Individuals and Groups. *IEEE Trans. Affect. Comput.* **2021**, *12*, 479–493. https://doi.org/10.1109/TAFFC.2018.2884461.
59. Koelstra, S.; Mühl, C.; Soleymani, M.; Lee, J.S.; Yazdani, A.; Ebrahimi, T.; Pun, T.; Nijholt, A.; Patras, I. DEAP: A Database for Emotion Analysis Using Physiological Signals. *IEEE Trans. Affect. Comput.* **2012**, *3*, 18–31. https://doi.org/10.1109/T-AFFC.2011.15.
60. Katsigiannis, S.; Ramzan, N. DREAMER: A Database for Emotion Recognition Through EEG and ECG Signals From Wireless Low-cost Off-the-Shelf Devices. *IEEE J. Biomed. Health Inform.* **2018**, *22*, 98–107. https://doi.org/10.1109/JBHI.2017.2688239.
61. Sharma, K.; Castellini, C.; van den Broek, E.L.; Albu-Schaeffer, A.; Schwenker, F. A Dataset of Continuous Affect Annotations and Physiological Signals for Emotion Analysis. *Sci. Data* **2019**, *6*, 196. https://doi.org/10.1038/s41597-019-0209-0.
62. Billman, G.E. The LF/HF Ratio Does Not Accurately Measure Cardiac Sympatho-Vagal Balance. *Front. Physiol.* **2013**, *4*, 26. https://doi.org/10.3389/fphys.2013.00026.
63. Task Force of the European Society of Cardiology and the North American Society of Pacing and Electrophysiology. Heart Rate Variability: Standards of Measurement, Physiological Interpretation, and Clinical Use. *Circulation* **1996**, *93*, 1043–1065. https://doi.org/10.1161/01.CIR.93.5.1043.
64. Bergholm, V.; Izaac, J.; Schuld, M.; Gogolin, C.; Ahmed, S.; Ajith, V.; Alam, M.S.; Alonso-Linaje, G.; AkashNarayanan, B.; Asadi, A.; et al. PennyLane: Automatic differentiation of hybrid quantum-classical computations. *arXiv* **2022**, arXiv:1811.04968.
65. Mitarai, K.; Negoro, M.; Kitagawa, M.; Fujii, K. Quantum circuit learning. *Phys. Rev. A* **2018**, *98*, 032309. https://doi.org/10.1103/PhysRevA.98.032309.
66. Schuld, M.; Bergholm, V.; Gogolin, C.; Izaac, J.; Killoran, N. Evaluating analytic gradients on quantum hardware. *Phys. Rev. A* **2019**, *99*, 032331. https://doi.org/10.1103/PhysRevA.99.032331.
67. Cerezo, M.; Arrasmith, A.; Babbush, R.; Benjamin, S.C.; Endo, S.; Fujii, K.; McClean, J.R.; Mitarai, K.; Yuan, X.; Cincio, L.; et al. Variational quantum algorithms. *Nat. Rev. Phys.* **2021**, *3*, 625–644. https://doi.org/10.1038/s42254-021-00348-9.
68. Lin, T.Y.; Goyal, P.; Girshick, R.; He, K.; Dollár, P. Focal Loss for Dense Object Detection. In *Proceedings of the 2017 IEEE International Conference on Computer Vision (ICCV)*; IEEE: Piscataway, NJ, USA, 2017; pp. 2999–3007. https://doi.org/10.1109/ICCV.2017.324.
69. Kingma, D.P.; Ba, J. Adam: A Method for Stochastic Optimization. *arXiv* **2015**, arXiv:1412.6980.
70. Kipf, T.N.; Welling, M. Semi-Supervised Classification with Graph Convolutional Networks. *arXiv* **2017**, arXiv:1609.02907.
71. Yeo, I.; Johnson, R.A. A new family of power transformations to improve normality or symmetry. *Biometrika* **2000**, *87*, 954–959. https://doi.org/10.1093/biomet/87.4.954.
72. Peng, H.; Long, F.; Ding, C. Feature selection based on mutual information criteria of max-dependency, max-relevance, and min-redundancy. *IEEE Trans. Pattern Anal. Mach. Intell.* **2005**, *27*, 1226–1238. https://doi.org/10.1109/TPAMI.2005.159.
73. Ke, G.; Meng, Q.; Finley, T.; Wang, T.; Chen, W.; Ma, W.; Ye, Q.; Liu, T.Y. LightGBM: A Highly Efficient Gradient Boosting Decision Tree. In *Proceedings of the Advances in Neural Information Processing Systems*; Guyon, I., Luxburg, U.V., Bengio, S., Wallach, H., Fergus, R., Vishwanathan, S., Garnett, R., Eds.; Curran Associates, Inc.: Red Hook, NY, USA, 2017; Volume 30, pp. 3146–3154.
74. Nixon, J.; Dusenberry, M.W.; Zhang, L.; Jerfel, G.; Tran, D. Measuring Calibration in Deep Learning. In *Proceedings of the IEEE/CVF Conference on Computer Vision and Pattern Recognition (CVPR) Workshops, Long Beach, CA, USA, 16–20 June 2019*; IEEE: Piscataway, NJ, USA, 2019; pp. 38–41.
75. Bustos, C.; Elhaouij, N.; Solé-Ribalta, A.; Borge-Holthoefer, J.; Lapedriza, A.; Picard, R. Predicting Driver Self-Reported Stress by Analyzing the Road Scene. In *Proceedings of the 2021 9th International Conference on Affective Computing and Intelligent Interaction (ACII)*; IEEE: Piscataway, NJ, USA, 2021; pp. 1–8. https://doi.org/10.1109/ACII52823.2021.9597438.
76. Nicolescu, Ş.T.; Adochiei, F.C.; Argatu, F.C.; Enache, B.A.; Seriţan, G.C. Deception Detection from Five-Channel Wearable EEG on LieWaves: A Reproducible Baseline for Subject-Dependent and Subject-Independent Evaluation. *Sensors* **2026**, *26*, 1027. https://doi.org/10.3390/s26031027.

77. He, M.; Alkurdi, A.; Clore, J.L.; Sowers, R.B.; Hsiao-Wecksler, E.T.; Hernandez, M.E. Scoping Review of ML Approaches in Anxiety Detection from In-Lab to In-the-Wild. *Appl. Sci.* **2025**, *15*, 10099. https://doi.org/10.3390/app151810099.
78. Baucas, M.J.; Spachos, P.; Plataniotis, K.N. Federated Learning and Blockchain-Enabled Fog-IoT Platform for Wearables in Predictive Healthcare. *IEEE Trans. Comput. Soc. Syst.* **2023**, *10*, 1732–1741. https://doi.org/10.1109/TCSS.2023.3235950.

Disclaimer/Publisher's Note: The statements, opinions and data contained in all publications are solely those of the individual author(s) and contributor(s) and not of MDPI and/or the editor(s). MDPI and/or the editor(s) disclaim responsibility for any injury to people or property resulting from any ideas, methods, instructions or products referred to in the content.
