# Supplementary material for: Trustworthy Stress Detection from Wearable Sensor Signals for Preventive Anxiety Monitoring: Subject-Independent Accuracy and Calibrated Confidence with the Autonomic-Branch Quantum-Graph Network
Source: Sensors (Basel). 2026 Oct 11;26(20):6425. doi: 10.3390/s26206425 (PMC13709010; doi:10.3390/s26206425)
Supplement: Supplementary file 1 [file sensors-26-06425-s001.zip › sensors-4523090-supplementary/supplementary_sensors_proof.pdf]

Supplementary Materials: Trustworthy Stress Detection from Wearable Sensor Signals for Preventive Anxiety Monitoring: Subject-Independent Accuracy and Calibrated Confidence with the Autonomic-Branch Quantum-Graph Network

Spyridon Papamentzelopoulos [1] and Sotirios Nikoletseas [1,*]

Contents

S1. Architecture comparison with prior stress/affect detection models 2
S2. Method and component comparison 4
S3. Expressivity of data re-uploading 7
S4. Trainability, gradients and optimization schedules 7
S5. Comparison with prior work under explicit evaluation protocols 9
S6. Data efficiency 11
S7. Per-edge correlator independence 12
S8. Full learned-coupling statistics 13
S9. Calibration leakage: full four-variant comparison 14
S10. Reproducibility 16
S11. Complete evaluation-inflation tables 17
S12. Candidate corpora and selection criteria 18
S13. Capacity of the classical twins and the realized register 20
S14. Baseline configuration, early-stopping sensitivity and multi-seed robustness 22
S15. Portability check on AffectiveROAD: full detail 24
S16. References 26

S1. Architecture comparison with prior stress/affect detection models

Representative classical, quantum, and hybrid models, grouped by computational paradigm, with the evaluation protocol stated explicitly for each entry.

Table S1. Architecture comparison of representative classical, quantum, and hybrid stress/affect-detection models.

Ref	Year	Paradigm	Model /architecture	Signals	Dataset	Classes	Evaluation protocol	Reported metric	Key limitation
[1]	2018	Classical	Handcrafted features + standard classifiers (RF/AB/LDA)	ECG, EDA, EMG, RESP, TEMP, ACC	WESAD	3 (+2)	LOSO (leakage-free)	$\leq$80% acc (3-cls); $\leq$93% acc (2-cls)	lab only; 15 subjects; single session
[2]	2018	Classical	Feature-based classifiers on fused sensors	HRV, EDA, posture, logging, facial	SWELL-KW	2–3	10-fold CV (not subject-wise) *and* LOSO, both reported	90.0% acc (10-fold) vs. 58.9% (LOSO), below the 61.8% majority baseline	quantifies its own leakage gap
[3]	2025	Deep learning	Multimodal feature fusion (time + frequency domain)	multi-physio	Nurse Stress Prediction (15 nurses)	3	no subject-level partitioning reported	91.0% acc	leakage-prone split
[4]	2021	Classical (personalized)	Per-user models	PPG, ACC	own (14 subjects, free-living)	2	personalized	macro-F1 up to 76%	needs per-user labels
[5]	2024	Deep learning	Multimodal automated emotion recognition	ECG, EDA	CASE, WESAD	4 & 3	nested stratified *k*-fold; no subject-level partitioning reported	86.7% (4-cls, CASE); 84.0% (3-cls, WESAD)	within-subject eval
[6]	2023	Classical (review)	Systematic review of generalizability	wearable physio	33 studies (2012–2022)	2–3	generalization emphasis	generalizable acc $\ll$ reported	meta-finding, not a model
[7]	2020	Deep learning	CNN/DNN + subject-wise model selection	IMU	HAR (10 subjects)	multi	LOSO vs. random	99.85% (non-LOSO) vs. 85.1% (LOSO)	activity, not stress
[8]	2022	**Quantum**	QSVM /VQC /QKNN + quantum-PCA	HRV, skin conductance	SWELL-KW	2	train/test split not reported	best QSVM acc 0.80 (HRV + PCA)	0.80 from a 3-feature HRV set; no split reported
[9]	2025	**Quantum**	Quantum-kernel SVM + tree-tensor-network QNN	GSR (VR)	own VR-GSR	2	80/20 held-out test	45% (QSVM-test); 55% (QNN-test)	low generalization (reported honestly)
[10]	2025	**Hybrid Q-C**	Quantum hybrid SVM (anomaly detection)	smartwatch; cortisol GT	own (40 older adults)	2 (anomaly)	random sampling; no subject-level partitioning reported	79% acc, F1 0.78; max recall 0.79 vs. 0.75 (classical SVM)	binary; small N
[11]	2024	**Hybrid Q-C**	Quantum-LSTM contrastive learning (QCLR)	HRV	WESAD-derived + 6 MH sets	2 (MH)	pretext + fine-tune split	**F1 0.9999**	no subject-level partitioning reported; inflation risk
[12]	2026	**Hybrid Q-C**	ResNet1D (attention) + adaptive quantum encoding (transfer)	HRV	WESAD + HRV stress sets	2 & 3	LOSO **and** stratified subject-independent (both reported)	**52.1% (3-class LOSO)** vs **99.97–99.99% (stratified)**	demonstrates the honest-versus-inflated gap within one study
**AB-QGN**	**(this work)**	**Hybrid quantum graph**	**Autonomic-branch QGNN: IsingZZ graph entanglers + trainable data re-uploading ($\leq$6 qubits) + classical context MLP + DANN subject-adversary + late fusion**	**HRV, EDA**	**WESAD, SWELL-KW**	**3**	**strict LOSO/GroupKFold (honest) *and* two deliberately inflated splits, same architecture**	**honest macro-F1 + ECE; entanglement & classical-twin ablations; quantifies the inflation others leave implicit**	**classically simulable at $\leq$6 qubits; no raw quantum-advantage claim**

S2. Method and component comparison

Each methodological building block of AB-QGN mapped to its original source work.

Table S2. Method and component comparison.

Ref	Year	Approach / Method	Signals	Dataset	Key result / contribution	Limitation
[1]	2018	Multimodal benchmark + baseline classifiers	ECG, EDA, EMG, RESP, TEMP, ACC	WESAD	Standard 3-class wearable affect benchmark	Lab stressors; 15 subjects
[13]	2014	Multimodal stress dataset	HRV, EDA, posture, logging	SWELL-KW	Knowledge-work stress benchmark w/ validated GT	Office-only; small N
[14]	2021	HRV signal-analysis review	HRV	25 articles	Catalogs HRV feature families & pipeline	No predictive model
[15]	2024	ML on fused GSR+PPG	EDA, PPG	Own (37 subjects)	GSR+PPG fusion; kNN 0.833 acc	Single session; split not reported
[4]	2021	Personalized models in the wild	PPG, ACC	Own (14 subjects)	Personalization closes free-living gap; macro-F1 up to 76%	Per-user data needed
[5]	2024	End-to-end deep multimodal	ECG, EDA	CASE, WESAD	Strong automated emotion recognition	Nested stratified k-fold; subject-wise split not reported
[6]	2023	Systematic review of generalizability	Wearable physio	33 studies (2012–2022)	Generalizable accuracy far below reported	Survey, not a model
[7]	2020	LOSO vs random split study	Wearable IMU	HAR (10 subjects)	99.85% (non-LOSO) vs. 85.1% (LOSO)	Activity, not stress
[16]	2025	Domain-invariant representation	EEG	SSVEP Benchmark (35 subjects)	Cross-subject invariance learning	Early-stage
[17]	2026	Leakage audit	MTS	GSTRIDE (frailty)	>98% originally reported; 96.6% reproduced, 58.8% after leakage correction	Diagnostic

continued

Table S2 – continued

Ref	Year	Approach / Method	Signals	Dataset	Key result / contribution	Limitation
[18]	2025	Sliding-window analysis	Time series	IT job-market series	Overlapping windows improve forecasting accuracy	Forecasting framing; leakage not assessed
[19]	2017	Focal loss	Images	COCO	Imbalance-robust loss (component)	Vision-origin
[20]	2017	Domain-adversarial training (DANN)	—	—	Gradient-reversal invariance (component)	Needs target data
[21]	2017	Temperature scaling	—	—	Fixes NN over-confidence (component)	Post-hoc only
[22]	2005	mRMR feature selection	—	—	Relevance/ redundancy selection (component)	Filter method
[23]	2020	Data re-uploading classifier	—	Synthetic	Few-qubit universality	Simulation
[24]	2014	Quantum SVM	—	—	Exponential-speedup kernel SVM	Idealized HW
[25]	2019	Quantum-enhanced feature spaces	—	—	Classically-hard quantum kernels	NISQ noise
[26]	2018	Barren-plateau theory	—	—	Gradients vanish w/qubits → shallow ansatz	Negative result
[27]	2024	Quantum CNN multiclass	Images	MNIST (4, 6, 8, 10 classes)	Hybrid Pennylane QCN; exceeds size-matched CNN at 6–10 classes	Not biosignal
[28]	2025	Hybrid quantum GNN	MRI images	Brain-tumour (253 images)	QGNN on MRI; 52% val acc vs. 31% classical GCNN	Static images; near-chance accuracy; no LOSO
[29]	2022	QML vs DL comparison	Tabular	PIMA Diabetes (768 records)	Classical DL 95% vs. QML 86% acc	Tabular, small data

continued

Table S2 – continued

Ref	Year	Approach / Method	Signals	Dataset	Key result / contribution	Limitation
[30]	2027	GNN-for-EEG survey	EEG	Many	Maps classical graph methods for emotion	No quantum
[31]	2023	Federated fog-IoT for wearables	IMU (HAR)	UCI HAR	Privacy-preserving deployment; 91.75% federated acc	No quantum core
**AB-QGN**	**—**	**Autonomic-branch hybrid quantum graph net + DANN + honest/inflated protocol**	**HRV, EDA**	**WESAD, SWELL-KW**	**Interpretable few-qubit graph core; calibrated; quantifies inflation under strict LOSO**	**Classically simulable at ≤6 qubits; no raw quantum-advantage claim**

S3. Expressivity of data re-uploading

Schuld, Sweke, and Meyer [32] show that a variational circuit which re-uploads a one-dimensional input x over L encoding layers results in a truncated Fourier series in that input,

$$f_\Theta(x) = \langle \psi(x)|M|\psi(x)\rangle = \sum_{n\in\Omega} c_n(\Theta)e^{inx}, \tag{S1}$$

where M is the measured observable, the accessible frequency spectrum Ω is dictated by the eigenvalues of the data-encoding generators (the generator $Y/2$, whose eigenvalue gaps ± 1 correspond to integer frequencies), and the coefficients $c_n(\Theta)$ are governed by the trainable rotation/entangling parameters. The size of Ω grows with the number of re-uploading layers L, as more repetitions of the encoding allow for higher frequencies: depth, not width, is the lever that expands expressivity. With L re-uploads of Q super-node inputs, the model spans a multivariate trigonometric polynomial whose reachable degree increases with L. Pérez-Salinas et al. [23] provide the complementary universality result: a single-qubit classifier with sufficient data re-uploading is a universal approximator, the quantum analog of a neural network in which each re-upload acts as a hidden-layer activation. Our design implements this principle and extends it to a Q-qubit entangled register, where the learned per-layer gains $\alpha_j^{(\ell)}$ function as re-uploading weights.

S4. Trainability, gradients and optimization schedules

Barren plateaus

For sufficiently expressive, randomly initialized circuits whose blocks approximate a 2-design, the cost-function gradient concentrates around zero:

$$\mathbb{E}[\partial_\theta\langle M\rangle] = 0, \qquad \mathrm{Var}[\partial_\theta\langle M\rangle] \sim \mathcal{O}(2^{-Q}), \tag{S2}$$

so the gradient signal decreases exponentially with the qubit number Q. At $Q \leq 6$—with shallow depth ($L = 3$), a structured non-random ansatz, and near-identity initialization—the variance is still large enough for stable optimization.

Parameter-shift rule

For a gate parameter θ generating eigenvalues $\pm\frac{1}{2}$,

$$\frac{\partial\langle P\rangle}{\partial\theta} = \frac{1}{2}\Big[\langle P\rangle_{\theta+\pi/2} - \langle P\rangle_{\theta-\pi/2}\Big], \tag{S3}$$

the hardware-compatible estimator [33,34]. We use exact reverse-mode backpropagation on the statevector simulator, which is free of shot noise—the shift rule allows us to port the same model to quantum hardware without changing the training procedure.

mRMR selection criterion

With S the already-selected set, the next feature maximizes

$$f^\star = \arg\max_{f\notin S} \frac{F(f)}{\frac{1}{|S|}\sum_{f'\in S}\rho_{f,f'} + \varepsilon}, \qquad \varepsilon = 10^{-9}, \tag{S4}$$

with $F(f)$ the ANOVA F-statistic and $\rho_{f,f'}$ the absolute Pearson correlation.

Adversarial ramp and learning-rate schedule

The gradient-reversal strength follows $\lambda(\mathrm{ep}) = \lambda_{\max}\big(2/(1+e^{-6\,\mathrm{ep}/(E-1)}) - 1\big)$ with $\lambda_{\max} = 0.5$. The learning rate follows linear warm-up (fraction 0.15) then cosine decay:

$$\eta(\mathrm{ep}) = \begin{cases} \eta_0 \dfrac{\mathrm{ep}+1}{E_{\mathrm{warm}}}, & \mathrm{ep} < E_{\mathrm{warm}}, \\ \frac{1}{2}\eta_0\Big(1+\cos\pi\frac{\mathrm{ep}-E_{\mathrm{warm}}}{E-E_{\mathrm{warm}}}\Big), & \mathrm{ep} \geq E_{\mathrm{warm}}. \end{cases} \tag{S5}$$

Classical message-passing twin

The structure-matched control replaces the variational circuit with

$$h^{(\ell+1)} = \mathrm{ReLU}\big(\hat{A}h^{(\ell)}W^{(\ell)}\big), \qquad \hat{A} = D^{-1/2}(A+I)D^{-1/2}, \tag{S6}$$

over the same super-node graph, with graph, features, depth, training schedule, and calibration split held identical, so any difference is attributable to the encoder alone. The hidden width is $h = 48$, so $W^{(\ell)} \in \mathbb{R}^{48\times48}$ and the encoder carries 7201 parameters against the circuit's 75 (Section S13); a strictly parameter-matched variant with $h = 4$ (73 parameters) is reported alongside it in the main text. At fusion level the twin reaches 51.99% against 61.42% honest macro-F1 on SWELL-KW and 83.71% against 86.18% on WESAD; the parameter-matched twin reaches 58.13% and 81.15%.

S5. Comparison with prior work under explicit evaluation protocols

Table S3. Comparison with prior work on WESAD and SWELL-KW. The key column is the "Protocol": subject-independent (LOSO or subject-grouped CV) results allow for direct comparison with ours; subject-dependent, window-overlapping, or unspecified splits are leakage-prone and systematically inflated (Section 4.2 of the main text). Acc = accuracy; F1 = macro-F1; cls = number of classes. AB-QGN refers to the honestly reported five-fold / LOSO results listed in Tables 2 and 3 of the main text.

#	Work	Year	Family	Dataset /signal	cls	Evaluation protocol	Reported headline	Calibration reported?
[1]	Schmidt *et al.* (WESAD baseline)	2018	Classical	WESAD (multi-modal)	3 /2	**LOSO /subject-independent (leakage-free)**	≤80% acc (3-cls); ≤93% (2-cls)	No
[2]	Koldijk *et al.* (SWELL baseline)	2018	Classical	SWELL-KW / multimodal	2	**10-fold CV (not subject-wise, inflated)**	90.0% acc	No
[2]	Koldijk *et al.* (SWELL baseline)	2018	Classical	SWELL-KW / multimodal	2	**LOSO (leakage-free)**	**58.9% acc, below the 61.8% majority baseline**	No
[35]	Adarsh & Gangadharan	2024	Classical (explainable GCN)	WESAD & SWELL / HRV	2	validation scheme not stated; no subject-level partitioning reported[b]	∼97.8% /∼94.5% acc	No
[11]	Padha & Sahoo (QCLR)	2024	Hybrid Q-C	WESAD-derived / HRV	2	pretext + fine-tune; no subject-level partitioning reported[b]	F1 0.9999	No
[12]	Zhu & Zhou (TL-QCHResnet)	2026	Hybrid Q-C	WESAD & SWELL / HRV	3 /2	**stratified / window-overlapping (inflated)[a]**	99.97–99.99% acc	No
[12]	Zhu & Zhou (TL-QCHResnet)	2026	Hybrid Q-C	WESAD /HRV	3 /2	**LOSO (leakage-free)**	**52.1% acc (3-cls); 86.1% (2-cls)**	No
[8]	Padha & Sahoo (QSVM/VQC/QKNN)	2022	Quantum	SWELL-KW / HRV+SC	2	train/test split, ratio unspecified; no subject-level partitioning reported[b]	best QSVM acc 0.80	No
[9]	Rababah & Farooq (QSVM /TTN-QNN)	2025	Quantum	own VR-GSR	2	held-out test split; no subject-level partitioning reported[b]	45% (QSVM); 55% (QNN)	No
[10]	Onim *et al.* (quantum hybrid SVM)	2025	Hybrid Q-C	own (40 adults) / smartwatch	2	no subject-level partitioning reported[b]; cortisol GT	higher recall vs. classical SVM	No
—	**AB-QGN (this work), WESAD fused**	**2026**	**Hybrid Q-C (graph)**	**WESAD / HRV+EDA**	**3**	**subject-grouped 5-fold (leakage-free)**	**86.7% acc /86.2 F1**	**Yes (ECE/ NLL/ Brier)**
—	**AB-QGN (this work), WESAD fused**	**2026**	**Hybrid Q-C (graph)**	**WESAD / HRV+EDA**	**3**	**LOSO (leakage-free)**	**84.4% acc /84.0 F1 /90.4 AUC**	**Yes**
—	**AB-QGN (this work), SWELL fused**	**2026**	**Hybrid Q-C (graph)**	**SWELL-KW / HRV+EDA**	**3**	**LOSO (leakage-free)**	**60.8% acc /55.1 F1**	**Yes**

[a] Zhu & Zhou describe this split as subject-independent. We classify it as inflated because the split is made by stratified sampling rather than by subject—and because the headline that results (99.97–99.99%) sits 14–48 points above the same authors' leave-one-subject-out result on the same data (52.1%/86.1%), a difference consistent with window-level leakage. Their subject-ID grouping applies to the leave-one-subject-out experiment, which we record separately as leakage-free. [b] These works do not specify how training and test partitions relate to subject identity; we record that absence rather than asserting a subject-dependent split.

S6. Data efficiency

Table S4. Data-efficiency curve (macro-F1, mean over three seeds). k = number of training subjects. Bold marks where AB-QGN matches or exceeds the best classical baseline at that k.

Dataset	Modality	k	AB-QGN	RF	GBM
WESAD	HRV	3	**61.2**	60.2	61.0
WESAD	HRV	5	61.3	64.2	66.2
WESAD	HRV	8	66.1	70.6	72.0
WESAD	EDA	3	70.6	72.9	72.0
WESAD	EDA	5	**66.6**	62.8	64.6
WESAD	EDA	8	70.0	66.0	72.2
SWELL	HRV	3	44.5	44.7	40.5
SWELL	HRV	5	43.2	44.1	43.0
SWELL	HRV	8	**46.9**	44.6	41.2
SWELL	HRV	12	47.8	48.5	44.2
SWELL	EDA	3	**31.0**	30.1	30.1
SWELL	EDA	5	29.8	32.3	33.0
SWELL	EDA	8	31.8	36.7	36.1
SWELL	EDA	12	35.3	34.2	36.4

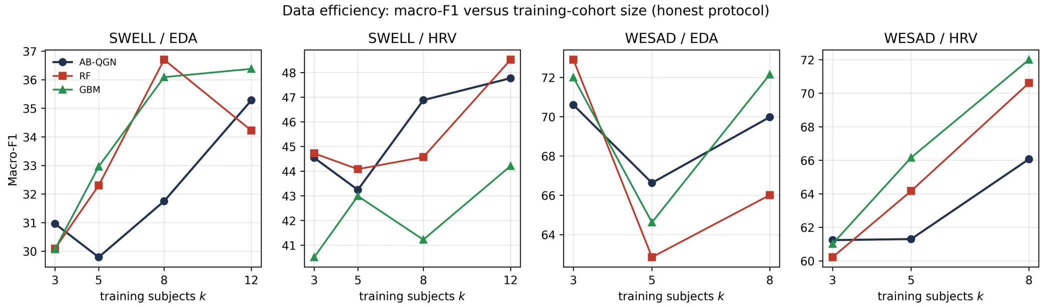

Figure S1. Macro-F1 versus number of training subjects k. AB-QGN sits 1.5 macro-F1 below the better classical baseline on average, narrowing to 0.35 at $k = 3$, where it leads in two of four cells.

S7. Per-edge correlator independence

Qubit indices follow the physiological branch order given in Section 3.2 of the main text: for HRV, 0 = vagal, 1 = sympathovagal, 2 = interval, 3 = nonlinear, 4 = relative-RR; for EDA, 0 = tonic, 1 = phasic, 2 = complexity, 3 = distribution, 4 = SCR peaks.

Table S5. Per-edge correlator independence. Coefficient of determination when each edge correlator $\langle Z_a Z_b \rangle$ is linearly regressed on the full set of single-qubit expectations, over all held-out windows—low values indicate that the two-qubit readout is not linearly recoverable from the single-qubit marginals.

Dataset	Modality	Edge (a, b)	R^2
WESAD	HRV	0-1	0.0507
WESAD	HRV	0-2	0.0781
WESAD	HRV	0-3	0.0696
WESAD	HRV	0-4	0.0732
WESAD	HRV	1-2	0.0580
WESAD	HRV	1-3	0.0478
WESAD	HRV	1-4	0.0271
WESAD	HRV	2-3	0.0619
WESAD	HRV	2-4	0.0610
WESAD	HRV	3-4	0.0419
WESAD	EDA	0-1	0.0192
WESAD	EDA	0-2	0.0199
WESAD	EDA	0-3	0.0292
WESAD	EDA	1-2	0.0236
WESAD	EDA	1-3	0.0141
WESAD	EDA	2-3	0.0130
SWELL	HRV	0-1	0.0231
SWELL	HRV	0-2	0.0183
SWELL	HRV	0-3	0.0563
SWELL	HRV	0-4	0.0095
SWELL	HRV	1-2	0.0042
SWELL	HRV	1-3	0.0335
SWELL	HRV	1-4	0.0038
SWELL	HRV	2-3	0.0358
SWELL	HRV	2-4	0.0040
SWELL	HRV	3-4	0.0330
SWELL	EDA	0-1	0.0053
SWELL	EDA	0-2	0.0085
SWELL	EDA	0-3	0.0111
SWELL	EDA	0-4	0.0155
SWELL	EDA	1-2	0.0157
SWELL	EDA	1-3	0.0064
SWELL	EDA	1-4	0.0124
SWELL	EDA	2-3	0.0184
SWELL	EDA	2-4	0.0238
SWELL	EDA	3-4	0.0203

S8. Full learned-coupling statistics

Cross-seed rank agreement is weak—the mean pairwise Spearman correlation between per-seed coupling vectors is −0.042 (WESAD/HRV), 0.352 (WESAD/EDA), 0.055 (SWELL/HRV), and −0.018 (SWELL/EDA).

Table S6. Full learned-coupling statistics. Per-edge mean, standard deviation, and coefficient of variation of the trained IsingZZ magnitude κ_{ab} across three seeds and all folds.

Dataset	Modality	Edge	Mean κ_{ab}	SD	CV
WESAD	HRV	vagal-sympathovagal	0.0800	0.0452	0.565
WESAD	HRV	vagal-interval	0.1254	0.0990	0.789
WESAD	HRV	vagal-nonlinear	0.1010	0.0666	0.660
WESAD	HRV	vagal-relativeRR	0.1433	0.1109	0.774
WESAD	HRV	sympathovagal-interval	0.1152	0.0548	0.476
WESAD	HRV	sympathovagal-nonlinear	0.0948	0.0568	0.600
WESAD	HRV	sympathovagal-relativeRR	0.0850	0.0410	0.483
WESAD	HRV	interval-nonlinear	0.1177	0.0825	0.700
WESAD	HRV	interval-relativeRR	0.1071	0.0556	0.519
WESAD	HRV	nonlinear-relativeRR	0.1004	0.0580	0.578
WESAD	EDA	tonic-phasic	0.1766	0.1273	0.721
WESAD	EDA	tonic-complexity	0.1613	0.1312	0.813
WESAD	EDA	tonic-distribution	0.1127	0.0996	0.884
WESAD	EDA	phasic-complexity	0.1435	0.0934	0.651
WESAD	EDA	phasic-distribution	0.1203	0.0663	0.551
WESAD	EDA	complexity-distribution	0.1192	0.0545	0.458
SWELL	HRV	vagal-sympathovagal	0.0955	0.0425	0.446
SWELL	HRV	vagal-interval	0.0922	0.0567	0.615
SWELL	HRV	vagal-nonlinear	0.1179	0.1075	0.912
SWELL	HRV	vagal-relativeRR	0.1084	0.0454	0.419
SWELL	HRV	sympathovagal-interval	0.1227	0.0603	0.491
SWELL	HRV	sympathovagal-nonlinear	0.1117	0.0504	0.451
SWELL	HRV	sympathovagal-relativeRR	0.0806	0.0242	0.300
SWELL	HRV	interval-nonlinear	0.1069	0.0755	0.706
SWELL	HRV	interval-relativeRR	0.1049	0.0630	0.600
SWELL	HRV	nonlinear-relativeRR	0.1228	0.0533	0.434
SWELL	EDA	tonic-phasic	0.1305	0.0721	0.553
SWELL	EDA	tonic-complexity	0.1601	0.0843	0.526
SWELL	EDA	tonic-distribution	0.1750	0.0743	0.424
SWELL	EDA	tonic-scr_peaks	0.1560	0.0760	0.487
SWELL	EDA	phasic-complexity	0.1233	0.0635	0.515
SWELL	EDA	phasic-distribution	0.1219	0.0608	0.499
SWELL	EDA	phasic-scr_peaks	0.1067	0.0649	0.608
SWELL	EDA	complexity-distribution	0.1357	0.0492	0.363
SWELL	EDA	complexity-scr_peaks	0.1640	0.0801	0.488
SWELL	EDA	distribution-scr_peaks	0.1520	0.0682	0.449

S9. Calibration leakage: full four-variant comparison

Section 4.3 of the main text reports two of the four calibration variants; all four are given here; the same gradient-boosting classifier is fit on the same leakage-free folds and calibrated in four ways—isotonic regression or temperature scaling, each with the calibration split drawn either by ordinary k-fold inside the training fold (non-grouped, which is conventional practice) or by GroupShuffleSplit so that no subject appears in both the fitting and calibration parts (subject-disjoint); and values are means over the three seeds $\{42, 7, 123\}$ with the standard deviation across seeds, each seed pooling five held-out folds.

Two readings matter. Both post-hoc methods improve by a comparable margin once the split is grouped—so the effect belongs to the split and not to the method. On SWELL-KW EDA, isotonic moves from 39.06 to 7.62 expected calibration error, and temperature scaling from 39.26 to 6.56. And the seed-to-seed standard deviations are at most 1.25 points, against effects of 7 to 32 points—so the gaps are 13 to 59 pooled standard deviations wide.

Table S7. Gradient boosting under four calibration schemes, honest five-fold protocol, window level, and pooled over held-out windows. Mean ± SD over three seeds. Lower is better for ECE, NLL, and Brier. Macro-F1 is included because the subject-disjoint variants fit one base model on 80% of the training fold rather than ensembling three, so their discrimination differs; it falls in three of the four dataset–modality cells, which is why the calibration gain cannot be attributed to a stronger base model.

Dataset	Modality	Calibration	ECE	NLL	Brier	Macro-F1
WESAD	HRV	isotonic, non-grouped	15.84 ± 0.21	3.5179 ± 0.0781	0.3827 ± 0.0032	71.53 ± 0.19
WESAD	HRV	temperature, non-grouped	20.67 ± 0.3	4.4312 ± 0.4617	0.4199 ± 0.003	72.18 ± 0.11
WESAD	HRV	isotonic, subject-disjoint	5.54 ± 0.78	1.074 ± 0.314	0.3549 ± 0.0129	66.7 ± 1.57
WESAD	HRV	temperature, subject-disjoint	4.06 ± 0.83	0.6227 ± 0.0306	0.3542 ± 0.0165	69.03 ± 1.13
WESAD	EDA	isotonic, non-grouped	16.55 ± 0.38	3.7755 ± 0.2152	0.3985 ± 0.0052	61.62 ± 0.13
WESAD	EDA	temperature, non-grouped	22.07 ± 0.48	5.1956 ± 0.4992	0.4448 ± 0.0096	63.55 ± 0.87
WESAD	EDA	isotonic, subject-disjoint	10.37 ± 0.86	2.7086 ± 0.2131	0.3989 ± 0.0193	60.15 ± 3.64
WESAD	EDA	temperature, subject-disjoint	9.23 ± 0.71	0.658 ± 0.0284	0.3547 ± 0.0051	65.97 ± 1.87
SWELL	HRV	isotonic, non-grouped	25.56 ± 0.14	6.2565 ± 0.0171	0.601 ± 0.0028	52.42 ± 0.3
SWELL	HRV	temperature, non-grouped	34.37 ± 0.24	8.3016 ± 0.6121	0.6919 ± 0.0035	53.36 ± 0.51
SWELL	HRV	isotonic, subject-disjoint	9.47 ± 1.0	1.2053 ± 0.264	0.5081 ± 0.0079	46.77 ± 1.93
SWELL	HRV	temperature, subject-disjoint	5.39 ± 0.48	0.8175 ± 0.0014	0.4701 ± 0.0004	51.45 ± 0.42
SWELL	EDA	isotonic, non-grouped	39.06 ± 0.24	4.4952 ± 0.0901	0.8822 ± 0.0039	40.91 ± 0.16
SWELL	EDA	temperature, non-grouped	39.26 ± 0.95	2.8144 ± 0.0544	0.8607 ± 0.007	40.24 ± 0.84
SWELL	EDA	isotonic, subject-disjoint	7.62 ± 1.25	1.39 ± 0.1977	0.6314 ± 0.024	32.65 ± 3.56
SWELL	EDA	temperature, subject-disjoint	6.56 ± 0.74	1.0511 ± 0.0115	0.6308 ± 0.0048	38.12 ± 0.19

Table S8. Fitted temperature by calibration split, across all 15 folds per cell (three seeds × five folds). The search grid spans [0.05, 6.00]. On a split that shares subjects with training, the temperature never exceeds 1.30 and sits at the grid floor in 36 of 60 folds—so calibration sharpens an already overconfident model. Fitted subject-disjoint, it moves to the other end and hits the ceiling in 29 of 60 folds, indicating that even the maximum available correction is insufficient. The two distributions have no overlap.

Dataset	Modality	Split	min	median	max	at floor	at ceiling
WESAD	HRV	non-grouped	0.05	0.05	0.76	11	0
WESAD	HRV	subject-disjoint	3.23	3.65	5.83	0	0
WESAD	EDA	non-grouped	0.05	0.05	1.30	12	0
WESAD	EDA	subject-disjoint	2.81	4.32	6.00	0	3
SWELL	HRV	non-grouped	0.05	0.05	0.76	13	0
SWELL	HRV	subject-disjoint	5.66	6.00	6.00	0	12
SWELL	EDA	non-grouped	0.66	0.85	0.98	0	0
SWELL	EDA	subject-disjoint	5.33	6.00	6.00	0	14

S10. Reproducibility

All results regenerate deterministically from the serialized configuration. Seeds $\{42, 7, 123\}$; $Q \leq 6$ — the realized layouts use $Q = 5$ for HRV on both datasets and for SWELL-KW EDA and $Q = 4$ for WESAD EDA, so the ceiling is never saturated; $L = 3$; PennyLane default.qubit statevector simulator with PyTorch backpropagation; noise analysis on default.mixed with a depolarizing channel at $p = 0.01$.

Two properties of the calibration procedure are recorded here because Section S9 turns on them. Both AB-QGN and the classical twin fit window-level temperature scaling on a subject-disjoint split of the training fold, drawn by GroupShuffleSplit. The fused session-level temperature is by contrast fitted in-sample over all sessions—with 45 and 51 sessions, per-fold fitting resulted in degenerate temperatures on nine-session sub-folds. Since temperature scaling is argmax-invariant, the fused macro-F1, accuracy, and AUC are unaffected, but the fused expected calibration error and negative log-likelihood are optimistically biased, and no calibration claim rests on them.

Distributed with the code archive: per-fold result tables for all three protocols; the matched per-fold AB-QGN vs. twin calibration pairs; the qubit sweep; the ablation grid; the depolarizing-noise and finite-shot re-evaluations; the 10,000-permutation label test on the fused session set; the sensitivity-matched alert-rate analysis; and the four-variant calibration-split experiment of Section S9. The revision additions are archived alongside them: the script that produced every check added in revision, the three-seed noise and qubit-sweep runs, the gradient-boosting early-stopping sensitivity, the parameter accounting, the realized-register layouts, the paired twin comparisons at window and session level, and, for the portability check, the derived AffectiveROAD window tables with their provenance record and the conversion utility.

Two files-executions in the archive give window-level macro-F1 for the $h = 48$ twin: calibration_qgn_vs_twin_perfold.csv, from the original parallel-fold run, and compare_window_full_vs_twin.csv together with twin_perfold_metrics.csv, from the revision rerun in which the folds were executed serially so that the hidden-width override reached the model. The two agree on the fused session-level scores to the last digit (83.71 and 51.99) and on every significance decision, though their per-cell window-level means differ by 0.2–0.7 macro-F1 because the serial execution consumes the random stream in a different order, and Cliff's δ on SWELL-KW EDA changes sign with the mean ($+0.08$ against -0.00); the AB-QGN model, which was not rerun serially, reproduces exactly. The main text quotes the revision rerun throughout, so that the calibration pairs of Table 6 and the macro-F1 pairs of Table 8 come from one execution and can be compared with the parameter-matched twin on equal terms.

S11. Complete evaluation-inflation tables

Table 4 of the main text shows nine of the fourteen dataset–modality–model configurations for space. Tables S9 and S10 give all fourteen under both regimes. Inflation is the leaky score minus the honest score on the identical model, features and training budget; the honest column of Table S9 is the five-fold value of Tables 2 and 4 of the main text, and that of Table S10 the LOSO value of Table 3. The 27–61 point range quoted in the abstract is the span of the per-modality (non-fused) cells of these two tables. Values are read from `table_leakage_gap_f1.csv` of the two main runs. Δ is computed on unrounded values and may therefore differ from the difference of the two rounded entries by 0.1.

Table S9. Evaluation-inflation gap in macro-F1 points, five-fold regime, all fourteen configurations. Honest = subject-grouped five-fold with all preprocessing, feature selection and calibration fitted in the training fold. Δ = leaky minus honest. Fused rows in bold. Leaky scores are rounded to one decimal: six configurations reach exactly 100.00 under both protocols, WESAD EDA (RF) reaches 100.00 and 99.89, and WESAD EDA (GBM) 99.99 and 99.92.

Dataset	Modality	Honest	Subject-dep.	Δ	Window-overlap	Δ
WESAD	HRV	62.5	96.7	+34.2	96.5	+34.1
WESAD	HRV (RF)	71.0	100.0	+29.0	100.0	+29.0
WESAD	HRV (GBM)	71.3	100.0	+28.7	100.0	+28.7
WESAD	EDA	66.3	94.9	+28.6	95.0	+28.7
WESAD	EDA (RF)	62.0	100.0	+38.0	99.9	+37.9
WESAD	EDA (GBM)	61.8	100.0	+38.2	99.9	+38.1
**WESAD**	**Fused**	**86.2**	**100.0**	**+13.8**	**100.0**	**+13.8**
SWELL	HRV	49.0	97.0	+48.0	96.7	+47.7
SWELL	HRV (RF)	53.6	100.0	+46.4	100.0	+46.4
SWELL	HRV (GBM)	51.8	100.0	+48.2	100.0	+48.2
SWELL	EDA	36.8	79.2	+42.4	74.0	+37.1
SWELL	EDA (RF)	37.8	96.1	+58.3	96.4	+58.6
SWELL	EDA (GBM)	40.0	96.6	+56.6	96.0	+56.0
**SWELL**	**Fused**	**61.4**	**100.0**	**+38.6**	**100.0**	**+38.6**

Table S10. Evaluation-inflation gap in macro-F1 points, LOSO regime, all fourteen configurations. Honest = strict leave-one-subject-out; the leaky protocols are the same stratified five-fold and jittered-duplicate constructions as in Table S9, so the honest column is the only one that changes. WESAD fused inflates by +16.0 and the SWELL-KW classical baselines by +52 to +61, as quoted in Section 4.2 of the main text. Δ is computed on unrounded values and may therefore differ from the difference of the two rounded entries by 0.1.

Dataset	Modality	Honest	Subject-dep.	Δ	Window-overlap	Δ
WESAD	HRV	63.1	96.7	+33.6	96.5	+33.4
WESAD	HRV (RF)	69.0	100.0	+31.0	100.0	+31.0
WESAD	HRV (GBM)	69.2	100.0	+30.8	100.0	+30.8
WESAD	EDA	67.6	94.9	+27.3	95.0	+27.4
WESAD	EDA (RF)	63.5	100.0	+36.5	99.9	+36.4
WESAD	EDA (GBM)	65.7	100.0	+34.3	99.9	+34.2
**WESAD**	**Fused**	**84.0**	**100.0**	**+16.0**	**100.0**	**+16.0**
SWELL	HRV	44.8	97.0	+52.1	96.7	+51.9
SWELL	HRV (RF)	47.5	100.0	+52.5	100.0	+52.5
SWELL	HRV (GBM)	46.0	100.0	+54.0	100.0	+54.0
SWELL	EDA	35.1	79.2	+44.1	74.0	+38.9
SWELL	EDA (RF)	35.9	96.1	+60.2	96.4	+60.4
SWELL	EDA (GBM)	35.7	96.6	+60.9	96.0	+60.3
**SWELL**	**Fused**	**55.1**	**100.0**	**+44.9**	**100.0**	**+44.9**

S12. Candidate corpora and selection criteria

A fixed-instrument measurement of the evaluation protocol imposes five requirements on a corpus: (C1) both an electrocardiographic inter-beat series for heart-rate variability and a skin-conductance channel for electrodermal activity, so that the two modalities of the instrument are both present; (C2) a three-class affective protocol with a non-stress class and at least two stress-related states, matching the classifier the instrument is built for; (C3) subject identifiers on every window, without which no subject-disjoint partition and no calibration-split experiment can be constructed; (C4) open access without a signed end-user agreement, so that the benchmark can be re-run by any reader; and (C5) a pre-computed windowed feature release by a group independent of ours, so that the pipeline adds no signal-level processing of its own and every difference between protocols is attributable to the partition alone. Table S11 lists the corpora we examined against these criteria, as documented by their authors. WESAD and SWELL-KW, through the feature release of Nkurikiyeyezu et al. [36,37], are to our knowledge the only pair that satisfies all five. Several corpora in the table satisfy the subject-identifier and access criteria (C3, C4) and carry at least one of the two required channels, and would be natural targets for the external validation named in Section 5.3 of the main text; each would require a signal-level front end of our own, which is the step the present design excludes by construction. Section S15 does exactly that for one of them, AffectiveROAD, as a portability check kept separate from the main results.

Table S11. Candidate public corpora for wearable stress detection against the five selection criteria. • = satisfied, – = not satisfied, ○ = partially (see note). Properties are those stated in each corpus's documentation; where labels are continuous or task-based rather than three affective classes this is noted under C2.

Corpus	Signals (cardiac / electrodermal)	C1	C2	C3	C4	C5	Note
WESAD [1] (via [36, 37])	chest ECG / EDA	•	•	•	•	•	used
SWELL-KW [13] (via [36,37])	chest ECG / finger SC	•	•	•	•	•	used
Stress Recognition in Automobile Drivers (PhysioNet drivedb) [38]	chest ECG / hand and foot EDA	•	○	•	•	–	three road-condition levels, but segment boundaries must be reconstructed from a marker channel that is incomplete in several records; raw signals only
Non-EEG dataset for assessment of neurological status (PhysioNet) [39]	wrist pulse-oximeter HR / wrist EDA	–	○	•	•	–	twenty subjects; four states (physical, cognitive and emotional stress, relaxation); heart rate at 1 Hz from an oximeter, no ECG; raw only
Nurse stress in the wild [40]	wrist PPG/IBI / wrist EDA	–	○	•	•	–	fifteen nurses on hospital shifts; stress episodes flagged by a detector and validated in end-of-shift surveys with a self-reported level, so labels cover episodes rather than whole conditions; no ECG; raw only—the natural target for the external validation of Section 5.3
AffectiveROAD [41]	chest strap (BioHarness) / wrist EDA (E4)	•	–	•	○	–	nine to ten drivers, 13–14 drives; a continuous 0–1 stress metric rated by an observer and validated by the driver, no class protocol; partial database on request. **Used for the portability check of Section S15**, with a front end of our own that computes HRV from the E4 photoplethysmographic stream rather than the chest ECG
UBFC-Phys [42]	wrist PPG / wrist EDA	–	•	•	○	–	rest / speech / arithmetic tasks; no ECG; registration required
StressID [43]	ECG / EDA (+ respiration)	•	○	•	–	–	65 participants, eleven tasks with per-task self-ratings of stress, relaxation, arousal and valence; proprietary licence signed by a permanently employed academic, non-commercial use only
CLAS [44]	ECG and PPG / EDA	•	–	•	○	–	cognitive-load tasks and emotional stimuli rather than stress classes; on request
MAUS [45]	ECG and PPG / GSR	•	–	•	•	–	N-back mental-workload levels, not affective stress
Stress-Predict [46]	wrist PPG / wrist EDA	–	–	•	•	–	binary baseline vs. stress tasks; no ECG
Wearable Exam Stress (PhysioNet) [47]	wrist PPG / wrist EDA	–	–	•	•	–	exam sessions, no three-class protocol; no ECG
AMIGOS [48], DEAP [49], DREAMER [50], CASE [51]	ECG or PPG / GSR (EEG-centric)	○	–	•	○	–	valence–arousal annotation, not stress classes

S13. Capacity of the classical twins and the realized register

Table S12 gives the exact parameter accounting of the quantum encoder, the structure-matched twin ($h = 48$) and the parameter-matched twin ($h = 4$), read off the class definitions of the released code with $L = 3$ layers, a 64-unit context MLP over the $K = 24$ mRMR-selected features where the modality has at least 24 (HRV on both corpora), and over all available features otherwise (22 on SWELL-KW EDA, 14 on WESAD EDA), and a 64-unit head. The variational circuit has $L(3Q + \binom{Q}{2})$ parameters (per-layer re-uploading gains α, entangler strengths ω on every edge, and two rotation angles per qubit); the graph convolution has $2h + L(h^2 + h) + h + 1$ (a lift from the scalar super-node signal to width h, L square propagation layers, and a projection back to one value per node). The angle-encoding scale and bias ($2Q$), the context MLP and the subject discriminator are shared; the head differs only through its input width, which is $Q + \binom{Q}{2} + 64$ for the quantum encoder and $Q + 64$ for the twins. The subject discriminator adds $(\text{input} \times 64 + 64) + (64 \times S + S)$ parameters, where S is the number of training subjects in the fold, identically up to the same input-width difference. Table S13 lists the register realized by each qubit budget of the sweep in Section 4.8 of the main text: the register is the ordered list of branches present in the modality's feature set, truncated to the budget, so any budget at or above the branch count realizes the same circuit.

Table S12. Parameter accounting of the encoders and full models (subject discriminator excluded). Readout is the dimension handed to the head. Totals differ between the quantum model and the twins by the encoder and by the head's first layer only.

Layout	Encoder	Encoder params	Readout	Angle enc.	Context MLP	Head	Total
HRV ($Q = 5$)	AB-QGN circuit	75	15	10	5760	5315	11,160
	GCN twin, $h = 48$	7201	5	10	5760	4675	17,646
	GCN twin, $h = 4$	73	5	10	5760	4675	10,518
SWELL EDA ($Q = 5$)	AB-QGN circuit	75	15	10	5632	5315	11,032
	GCN twin, $h = 48$	7201	5	10	5632	4675	17,518
	GCN twin, $h = 4$	73	5	10	5632	4675	10,390
WESAD EDA ($Q = 4$)	AB-QGN circuit	54	10	8	5120	4995	10,177
	GCN twin, $h = 48$	7201	4	8	5120	4611	16,940
	GCN twin, $h = 4$	73	4	8	5120	4611	9,812

Table S13. Register realized by each qubit budget. Branches are taken in the physiological priority order of Section 3.2 of the main text and truncated to the budget; features of a truncated branch join the last retained one. Identical rows execute the identical circuit, so the equality of the sweep from $Q = 5$ onward is structural and independent of the seed.

Dataset / modality	Budget	Realized Q	Branches on the register
WESAD HRV, SWELL-KW HRV	4	4	vagal, sympathovagal, interval, nonlinear (+ relative-RR merged)
	5, 6, 8	5	vagal, sympathovagal, interval, nonlinear, relative-RR
WESAD EDA	4, 5, 6, 8	4	tonic, phasic, complexity, distribution
SWELL-KW EDA	4	4	tonic, phasic, complexity, distribution (+ SCR peaks merged)
	5, 6, 8	5	tonic, phasic, complexity, distribution, SCR peaks

S14. Baseline configuration, early-stopping sensitivity and multi-seed robustness

Baseline configuration.

The random forest is RandomForestClassifier with n_estimators=300 and is fixed at class_weight="balanced" with scikit-learn defaults otherwise; the gradient-boosting baseline is HistGradientBoostingClassifier with learning_rate=0.06, max_iter=400, regularization at l2_regularization=1.0, and early_stopping=True, wrapped for the reported rows in CalibratedClassifierCV using the parameters method="isotonic" and cv=3. It is worth mentioning that no search over these values was performed at any point; they were just set once and applied to every dataset, modality, fold, seed and protocol. The two internal splits that are not subject-grouped are the early-stopping validation subset (scikit-learn's default random 10% of the training fold) and the three-fold calibrator cross-fitting; the latter is the conventional practice measured in Section S9.

Early-stopping sensitivity.

Table S14 refits the uncalibrated gradient-boosting base model using the honest five-fold partitioning under three early-stopping rules: scikit-learn's random 10% validation subset (as reported), no early stopping with the full 400 iterations, and a subject-disjoint 10% validation subset created by GroupShuffleSplit where the iteration count has been selected by log-loss on that subset and the model refitted on the whole training fold. Differences are tracked with the paired Wilcoxon test over the 15-fold $\times$ seed pairs per cell.

Table S14. Gradient-boosting macro-F1 under three early-stopping rules, honest five-fold protocol, uncalibrated base model, mean $\pm$ SD over three seeds $\times$ five folds, with the mean number of boosting iterations in parentheses.

Dataset	Modality	p_{Holm} (random vs. grouped)	random 10% (reported)	no early stopping	subject-grouped 10%
SWELL	EDA	0.179	39.2 $\pm$ 5.0 (394 it.)	39.3 $\pm$ 5.3 (400 it.)	34.3 $\pm$ 8.1 (7 it.)
SWELL	HRV	0.212	52.8 $\pm$ 6.8 (400 it.)	52.8 $\pm$ 6.6 (400 it.)	50.0 $\pm$ 5.4 (16 it.)
WESAD	EDA	0.0005	64.8 $\pm$ 5.8 (399 it.)	64.7 $\pm$ 6.8 (400 it.)	57.8 $\pm$ 5.0 (11 it.)
WESAD	HRV	1.000	72.2 $\pm$ 6.4 (400 it.)	72.0 $\pm$ 6.4 (400 it.)	71.8 $\pm$ 6.1 (32 it.)

Depolarizing noise across seeds.

Table S15 repeats the re-evaluation of Section 4.10 of the main text for the three seeds $\{42, 7, 123\}$ with the same reduced epoch budget.

Table S15. Per-seed noiseless versus depolarizing ($p = 0.01$) results, WESAD, honest protocol. Each cell gives noiseless / noisy / Δ for that seed; Table 10 of the main text reports the mean, SD and range over the three seeds.

Modality	Metric	Seed 42	Seed 7	Seed 123
HRV	ECE	10.03 / 10.02 / −0.01	11.88 / 11.60 / −0.28	11.80 / 11.24 / −0.56
HRV	NLL	0.755 / 0.757 / +0.002	0.783 / 0.780 / −0.003	0.745 / 0.739 / −0.006
HRV	Brier	0.452 / 0.452 / +0.001	0.443 / 0.444 / +0.001	0.453 / 0.449 / −0.004
HRV	Macro-F1	62.66 / 62.61 / −0.05	63.71 / 64.55 / +0.84	60.28 / 61.05 / +0.77
HRV	Accuracy	66.03 / 66.04 / +0.01	68.10 / 67.82 / −0.28	63.29 / 64.11 / +0.82
EDA	ECE	16.73 / 18.68 / +1.95	21.43 / 20.74 / −0.69	19.59 / 19.74 / +0.15
EDA	NLL	1.017 / 1.038 / +0.021	0.934 / 0.915 / −0.018	1.560 / 1.559 / −0.001
EDA	Brier	0.403 / 0.418 / +0.015	0.413 / 0.406 / −0.007	0.447 / 0.447 / −0.000
EDA	Macro-F1	66.89 / 66.61 / −0.28	68.87 / 68.21 / −0.67	67.62 / 67.65 / +0.03
EDA	Accuracy	75.67 / 75.15 / −0.52	76.56 / 76.58 / +0.02	74.93 / 75.02 / +0.09

Qubit budget across seeds.

Table S16 repeats the $Q = 4$ against $Q = 5$ comparison of Section 4.8 of the main text for the three seeds, fusing each seed separately; budgets of 6 and 8 are omitted because they realize the $Q = 5$ circuit exactly (Table S13).

Table S16. Honest fused macro-F1 at $Q = 4$ and $Q = 5$ per seed, with the window-level paired comparison over 15 fold $\times$ seed pairs. WESAD EDA has four branches, so both budgets realize the same circuit there and the paired difference is identically zero.

		Fused macro-F1		Window level, $Q = 5$ minus $Q = 4$	
Dataset	Seed	$Q = 4$	$Q = 5$	HRV	EDA
WESAD	42	83.71	86.18	$+0.5$, $p_{\mathrm{Holm}} = 1.0$	0.0 (identical)
WESAD	7	77.11	82.00		
WESAD	123	78.72	76.62		
SWELL	42	61.77	61.42	-1.0, $p_{\mathrm{Holm}} = 1.0$	-0.1, $p_{\mathrm{Holm}} = 1.0$
SWELL	7	55.08	69.98		
SWELL	123	57.25	63.10		

S15. Portability check on AffectiveROAD: full detail

Section 4.11 of the main text reports the portability check on AffectiveROAD [41] under the five-fold regime with the band labels, and Table 11 there gives the inflation of every model. This section documents the rest: the front end in full, the strict leave-one-subject-out regime, two alternative labelling rules, and the calibration-split experiment of Section S9 repeated on this corpus. The corpus enters through the same feature-table interface as the two release corpora, nothing inside the pipeline changed, and the conversion script is in the code archive; the criteria it fails as a fixed-instrument corpus are C5 of Section S12, a pre-computed feature release by an independent group, and C2, a three-class protocol, for which its banded stress rating stands in. This is why it carries the protocol claim and no claim about any model.

Corpus and front end.

AffectiveROAD records nine drivers over thirteen real-world drives of about 85 minutes each (rest, city and highway segments), with an Empatica E4 wristband and a Zephyr BioHarness chest strap, and provides a continuous 0–1 stress metric rated by an observer in the car and validated by the driver afterwards. We computed the heart-rate-variability indices of the release from the Empatica E4 photoplethysmographic inter-beat-interval stream in 60-second windows stepped every five seconds (8152 windows; 30 of the 34 release columns; the four absent ones are higher-order relative-RR statistics SDSD_REL_RR, SDRR_RMSSD_REL_RR, KURT_REL_RR and SKEW_REL_RR, optional to the pipeline as sample entropy and Higuchi fractal dimension are on WESAD, and four relative-RR features remain), so the HRV register realizes all five branches. The stream is reported only where intervals pass the device's quality filter, so the retained intervals cover a median of 48.7 of every 60 seconds (range 22.3–59.9) and the successive-difference indices are computed across those gaps; very-low-frequency power is exactly zero in 64% of windows and contributes 3.9% of total power on average, so two of the five sympathovagal features are uninformative here. The electrodermal table carries the nine release features for which our extraction has an approximate counterpart (MEAN from the tonic level, MEAN_1ST_GRAD from the slope, and the mean and maximum SCR amplitude for MEAN_PEAKS and MAX_PEAKS), so the EDA register realizes four branches; the complexity branch is absent. The corpus provides no protocol condition per window in the files we used, so the three classes are bands of the stress metric fixed before any model was fitted: low ($< 1/3$), medium ($1/3$–$2/3$) and high ($\geq 2/3$), giving 809, 2322 and 5021 windows. The subject identifier is the driver, and a session is a driver $\times$ band, 27 in all. Because a band is defined by the label, such a session pools non-contiguous windows already known to share a class and is not a unit a monitor could observe. The conversion utility also supports two alternative labelling rules, discussed below.

Beyond the five-fold band-label result of the main text.

Under strict leave-one-subject-out (nine folds, three seeds) the honest scores are 31.6 (HRV), 30.5 (EDA) and 49.8 fused, so the picture of Table 11 of the main text does not depend on the cross-validation regime. The three classes are bands of the continuous metric at one third and two thirds, fixed before any model was fitted; the conversion utility also implements the empirical tertiles, and the discretization established for this corpus by Bustos et al. [52] can be obtained from it by setting the two cut points. We did not run those variants, so we make no claim about how the fused score moves with the labelling rule, and no conclusion drawn here depends on the particular cuts: the inflation under the leaky protocols is a property of the partition, measured with the labels held fixed. The calibration-split experiment of Section S9 reproduces its effect here for the same calibrator: temperature

scaling fitted on a subject-sharing split leaves the gradient-boosting expected calibration error at 21.0 (HRV) and 29.9 (EDA), and fitted subject-disjointly at 6.7 and 5.0, the same split-driven divergence as Tables S7 and S8. Isotonic calibration on the subject-sharing split reads 4.6 and 7.6 only because its argmax collapses to the majority class (macro-F1 25.7 and 25.6), which is why the main text quotes the temperature rows. Isotonic regression fitted subject-disjointly reads 10.95 (HRV) and 10.35 (EDA), with macro-F1 27.3 and 29.9, which completes the four variants. Per-class fused scores under the honest protocol are 85.7 (low stress), 52.6 (medium) and 42.9 (high): the model recovers the nine low-stress sessions and confuses the two stress bands with each other.

What could raise the honest scores on this corpus: protocol labels (rest, highway, city) rather than bands of an observer rating, and the full electrodermal feature set computed from the raw signal. Both require the drive annotations and recordings, which is the external validation named in Section 5.3 of the main text. As Section 4.11 states, no conclusion about the encoder is drawn here.

*References in this supplement are numbered **independently** of the main manuscript.*

References

1. Schmidt, P.; Reiss, A.; Duerichen, R.; Marberger, C.; Van Laerhoven, K. Introducing WESAD, a Multimodal Dataset for Wearable Stress and Affect Detection. In Proceedings of the 20th ACM International Conference on Multimodal Interaction, New York, NY, USA, 2018; ICMI '18, p. 400–408. https://doi.org/10.1145/3242969.3242985.
2. Koldijk, S.; Neerincx, M.A.; Kraaij, W. Detecting Work Stress in Offices by Combining Unobtrusive Sensors. *IEEE Transactions on Affective Computing* **2018**, *9*, 227–239. https://doi.org/10.1109/TAFFC.2016.2610975.
3. Xiang, J.Z.; Wang, Q.Y.; Fang, Z.B.; Esquivel, J.A.; Su, Z.X. A multi-modal deep learning approach for stress detection using physiological signals: integrating time and frequency domain features. *Frontiers in Physiology* **2025**, *16*, 1584299. https://doi.org/10.3389/fphys.2025.1584299.
4. Tazarv, A.; Labbaf, S.; Reich, S.M.; Dutt, N.; Rahmani, A.M.; Levorato, M. Personalized Stress Monitoring using Wearable Sensors in Everyday Settings. In Proceedings of the 2021 43rd Annual International Conference of the IEEE Engineering in Medicine & Biology Society (EMBC), 2021, pp. 7332–7335. https://doi.org/10.1109/EMBC46164.2021.9630224.
5. Sriram Kumar, P.; Govarthan, P.K.; Aleem Shaik Gadda, A.; Ganapathy, N.; Fredo Agastinose Ronickom, J. Deep Learning-Based Automated Emotion Recognition Using Multimodal Physiological Signals and Time-Frequency Methods. *IEEE Transactions on Instrumentation and Measurement* **2024**, *73*, 1–12. https://doi.org/10.1109/TIM.2024.3420349.
6. Vos, G.; Trinh, K.; Sarnyai, Z.; Rahimi Azghadi, M. Generalizable machine learning for stress monitoring from wearable devices: A systematic literature review. *International Journal of Medical Informatics* **2023**, *173*, 105026. https://doi.org/10.1016/j.ijmedinf.2023.105026.
7. Gholamiangonabadi, D.; Kiselov, N.; Grolinger, K. Deep Neural Networks for Human Activity Recognition With Wearable Sensors: Leave-One-Subject-Out Cross-Validation for Model Selection. *IEEE Access* **2020**, *8*, 133982–133994. https://doi.org/10.1109/ACCESS.2020.3010715.
8. Padha, A.; Sahoo, A. Quantum Enhanced Machine Learning for Unobtrusive Stress Monitoring. In Proceedings of the 2022 Fourteenth International Conference on Contemporary Computing, New York, NY, USA, 2022; IC3-2022, p. 476–483. https://doi.org/10.1145/3549206.3549288.
9. Rababah, B.; Farooq, B. Quantum Machine Learning in Transportation: A Case Study of Pedestrian Stress Modelling. In Proceedings of the 2025 IEEE 28th International Conference on Intelligent Transportation Systems (ITSC), 2025, pp. 3054–3059. https://doi.org/10.1109/ITSC60802.2025.11423545.
10. Onim, M.S.H.; Humble, T.S.; Thapliyal, H. Quantum Hybrid Support Vector Machines for Stress Detection in Older Adults. In Proceedings of the 2025 IEEE International Conference on Consumer Electronics (ICCE), 2025, pp. 1–4. https://doi.org/10.1109/ICCE63647.2025.10929873.
11. Padha, A.; Sahoo, A. QCLR: Quantum-LSTM contrastive learning framework for continuous mental health monitoring. *Expert Systems with Applications* **2024**, *238*, 121921. https://doi.org/10.1016/j.eswa.2023.121921.
12. Zhu, Z.; Zhou, R. TL-QCHResnet: Quantum transfer learning based on heart rate variability data. *Biomedical Signal Processing and Control* **2026**, *123*, 110563. https://doi.org/10.1016/j.bspc.2026.110563.
13. Koldijk, S.; Sappelli, M.; Verberne, S.; Neerincx, M.A.; Kraaij, W. The SWELL Knowledge Work Dataset for Stress and User Modeling Research. In Proceedings of the 16th International Conference on Multimodal Interaction, New York, NY, USA, 2014; ICMI '14, p. 291–298. https://doi.org/10.1145/2663204.2663257.
14. Ishaque, S.; Khan, N.; Krishnan, S. Trends in Heart-Rate Variability Signal Analysis. *Frontiers in Digital Health* **2021**, *3*, 639444. https://doi.org/10.3389/fdgth.2021.639444.
15. Nechyporenko, A.; Frohme, M.; Strelchuk, Y.; Omelchenko, V.; Gargin, V.; Ishchenko, L.; Alekseeva, V. Galvanic Skin Response and Photoplethysmography for Stress Recognition Using Machine Learning and Wearable Sensors. *Applied Sciences* **2024**, *14*, 11997. https://doi.org/10.3390/app142411997.

16. R, R.V.S.S.B.; Abdohwr, R.; M, K.K.; T, M.; Devika, P. Domain-Invariant Representation Learning for Cross-Subject Biomedical Signal Analysis. In Proceedings of the 2025 International Conference on Intelligent Communication Networks and Computational Techniques (ICICNCT), Sep. 2025, pp. 1–6. https://doi.org/10.1109/ICICNCT66124.2025.11232887.
17. Hughes, C.M.L.M.; Zhang, Y. Bursting the bubble: data leakage and inflated deep learning accuracy in multivariate time-series frailty classification. *Frontiers in Computer Science* **2026**, *8*, 1700489. https://doi.org/10.3389/fcomp.2026.1700489.
18. Ndungi, R.; Blekanov, I.S. Improving time series forecasting by applying the sliding window approach. *Computational nanotechnology* **2025**, *12*, 11–18. https://doi.org/10.33693/2313-223x-2025-12-2-11-18.
19. Lin, T.Y.; Goyal, P.; Girshick, R.; He, K.; Dollár, P. Focal Loss for Dense Object Detection. In Proceedings of the 2017 IEEE International Conference on Computer Vision (ICCV), 2017, pp. 2999–3007. https://doi.org/10.1109/ICCV.2017.324.
20. Ganin, Y.; Ustinova, E.; Ajakan, H.; Germain, P.; Larochelle, H.; Laviolette, F.; Marchand, M.; Lempitsky, V. Domain-Adversarial Training of Neural Networks. In *Domain Adaptation in Computer Vision Applications*; Csurka, G., Ed.; Springer International Publishing: Cham, 2017; pp. 189–209. https://doi.org/10.1007/978-3-319-58347-1_10.
21. Guo, C.; Pleiss, G.; Sun, Y.; Weinberger, K.Q. On Calibration of Modern Neural Networks. In Proceedings of the 34th International Conference on Machine Learning; Precup, D.; Teh, Y.W., Eds. PMLR, 06–11 Aug 2017, Vol. 70, *Proceedings of Machine Learning Research*, pp. 1321–1330.
22. Peng, H.; Long, F.; Ding, C. Feature selection based on mutual information criteria of max-dependency, max-relevance, and min-redundancy. *IEEE Transactions on Pattern Analysis and Machine Intelligence* **2005**, *27*, 1226–1238. https://doi.org/10.1109/TPAMI.2005.159.
23. Pérez-Salinas, A.; Cervera-Lierta, A.; Gil-Fuster, E.; Latorre, J.I. Data re-uploading for a universal quantum classifier. *Quantum* **2020**, *4*, 226. https://doi.org/10.22331/q-2020-02-06-226.
24. Rebentrost, P.; Mohseni, M.; Lloyd, S. Quantum Support Vector Machine for Big Data Classification. *Phys. Rev. Lett.* **2014**, *113*, 130503. https://doi.org/10.1103/PhysRevLett.113.130503.
25. Havlíček, V.; Córcoles, A.D.; Temme, K.; Harrow, A.W.; Kandala, A.; Chow, J.M.; Gambetta, J.M. Supervised learning with quantum-enhanced feature spaces. *Nature* **2019**, *567*, 209–212. https://doi.org/10.1038/s41586-019-0980-2.
26. McClean, J.R.; Boixo, S.; Smelyanskiy, V.N.; Babbush, R.; Neven, H. Barren plateaus in quantum neural network training landscapes. *Nature Communications* **2018**, *9*, 4812. https://doi.org/10.1038/s41467-018-07090-4.
27. Mordacci, M.; Ferrari, D.; Amoretti, M. Multi-Class Quantum Convolutional Neural Networks. In Proceedings of the 2024 Workshop on Quantum Search and Information Retrieval, New York, NY, USA, 2024; QUASAR ’24, p. 9–16. https://doi.org/10.1145/3660318.3660326.
28. Rajamohana, S.P.; Yelamali, V.; Soni, P.; Vanahalli, M.K.; Prasad, P. Hybrid Quantum Graph Neural Network for Brain Tumor MR Image Classification. *Journal of Scientific & Industrial Research (JSIR)* **2025**, *84*, 60–71. https://doi.org/10.56042/jsir.v84i1.14112.
29. Gupta, H.; Varshney, H.; Sharma, T.K.; Pachauri, N.; Verma, O.P. Comparative performance analysis of quantum machine learning with deep learning for diabetes prediction. *Complex & Intelligent Systems* **2022**, *8*, 3073–3087. https://doi.org/10.1007/s40747-021-00398-7.
30. Liu, C.; Deng, Y.; Wu, Y.; Yang, R.; Wang, Z.; Zhang, L.; Chen, S.; Zhang, T.; Liu, Y.; Ding, Y.; et al. Graph Neural Networks in EEG-Based Emotion Recognition: A Survey. In *Data Science: Foundations and Applications*; Wong, R.C.W.; Tong, H.; Lu, H.; Kwok, J.; Salim, F.; Song, Y.; Yiu, M.L., Eds.; Springer Nature Singapore: Singapore, 2027; pp. 581–598. https://doi.org/10.1007/978-981-92-1947-6_46.
31. Baucas, M.J.; Spachos, P.; Plataniotis, K.N. Federated Learning and Blockchain-Enabled Fog-IoT Platform for Wearables in Predictive Healthcare. *IEEE Transactions on Computational Social Systems* **2023**, *10*, 1732–1741. https://doi.org/10.1109/TCSS.2023.3235950.
32. Schuld, M.; Sweke, R.; Meyer, J.J. Effect of data encoding on the expressive power of variational quantum-machine-learning models. *Phys. Rev. A* **2021**, *103*, 032430. https://doi.org/10.1103/PhysRevA.103.032430.
33. Mitarai, K.; Negoro, M.; Kitagawa, M.; Fujii, K. Quantum circuit learning. *Phys. Rev. A* **2018**, *98*, 032309. https://doi.org/10.1103/PhysRevA.98.032309.

34. Schuld, M.; Bergholm, V.; Gogolin, C.; Izaac, J.; Killoran, N. Evaluating analytic gradients on quantum hardware. *Phys. Rev. A* **2019**, *99*, 032331. https://doi.org/10.1103/PhysRevA.99.032331.
35. Adarsh, V.; Gangadharan, G.R. Mental stress detection from ultra-short heart rate variability using explainable graph convolutional network with network pruning and quantisation. *Machine Learning* **2024**, *113*, 5467–5494. https://doi.org/10.1007/s10994-023-06504-9.
36. Nkurikiyeyezu, K. Biometrics for Stress Monitoring. Kaggle dataset. Available online: https://www.kaggle.com/datasets/qiriro/stress, 2019. (accessed on 28 July 2026).
37. Nkurikiyeyezu, K.; Yokokubo, A.; Lopez, G. Effect of Person-specific Biometrics in Improving Generic Stress Predictive Models. *Sensors and Materials* **2020**, *32*, 703–722. https://doi.org/10.18494/SAM.2020.2650.
38. Healey, J.A.; Picard, R.W. Detecting Stress During Real-World Driving Tasks Using Physiological Sensors. *IEEE Transactions on Intelligent Transportation Systems* **2005**, *6*, 156–166. https://doi.org/10.1109/TITS.2005.848368.
39. Birjandtalab, J.; Cogan, D.; Pouyan, M.B.; Nourani, M. A Non-EEG Biosignals Dataset for Assessment and Visualization of Neurological Status. In Proceedings of the 2016 IEEE International Workshop on Signal Processing Systems (SiPS), Oct 2016, pp. 110–114. https://doi.org/10.1109/SiPS.2016.27.
40. Hosseini, S.; Gottumukkala, R.; Katragadda, S.; Bhupatiraju, R.T.; Ashkar, Z.; Borst, C.W.; Cochran, K. A Multimodal Sensor Dataset for Continuous Stress Detection of Nurses in a Hospital. *Scientific Data* **2022**, *9*, 255. https://doi.org/10.1038/s41597-022-01361-y.
41. Haouij, N.E.; Poggi, J.M.; Sevestre-Ghalila, S.; Ghozi, R.; Jaïdane, M. AffectiveROAD system and database to assess driver's attention. In Proceedings of the 33rd Annual ACM Symposium on Applied Computing, New York, NY, USA, 2018; SAC '18, p. 800–803. https://doi.org/10.1145/3167132.3167395.
42. Sabour, R.M.; Benezeth, Y.; De Oliveira, P.; Chappé, J.; Yang, F. UBFC-Phys: A Multimodal Database For Psychophysiological Studies of Social Stress. *IEEE Transactions on Affective Computing* **2023**, *14*, 622–636. https://doi.org/10.1109/TAFFC.2021.3056960.
43. Chaptoukaev, H.; Strizhkova, V.; Panariello, M.; Dalpaos, B.; Reka, A.; Manera, V.; Thümmler, S.; Ismailova, E.; W., N.; Bremond, F.; et al. StressID: a Multimodal Dataset for Stress Identification. In Proceedings of the Advances in Neural Information Processing Systems 36. Neural Information Processing Systems Foundation, Inc. (NeurIPS), 2023, NeurIPS 2023, p. 29798–29811. https://doi.org/10.52202/075280-1297.
44. Markova, V.; Ganchev, T.; Kalinkov, K. CLAS: A Database for Cognitive Load, Affect and Stress Recognition. In Proceedings of the 2019 International Conference on Biomedical Innovations and Applications (BIA), Nov 2019, pp. 1–4. https://doi.org/10.1109/BIA48344.2019.8967457.
45. Beh, W.K.; Wu, Y.H.; Wu, A.Y. MAUS: A Dataset for Mental Workload Assessment on N-Back Task Using Wearable Sensor, 2021. arXiv:2111.02561.
46. Iqbal, T.; Simpkin, A.J.; Roshan, D.; Glynn, N.; Killilea, J.; Walsh, J.; Molloy, G.; Ganly, S.; Ryman, H.; Coen, E.; et al. Stress Monitoring Using Wearable Sensors: A Pilot Study and Stress-Predict Dataset. *Sensors* **2022**, *22*, 8135. https://doi.org/10.3390/s22218135.
47. Amin, M.R.; Wickramasuriya, D.; Faghih, R.T. A Wearable Exam Stress Dataset for Predicting Cognitive Performance in Real-World Settings (version 1.0.0). PhysioNet. Available online: https://physionet.org/content/wearable-exam-stress/1.0.0/, 2022. (accessed on 3 August 2026).
48. Miranda-Correa, J.A.; Abadi, M.K.; Sebe, N.; Patras, I. AMIGOS: A Dataset for Affect, Personality and Mood Research on Individuals and Groups. *IEEE Transactions on Affective Computing* **2021**, *12*, 479–493. https://doi.org/10.1109/TAFFC.2018.2884461.
49. Koelstra, S.; Mühl, C.; Soleymani, M.; Lee, J.S.; Yazdani, A.; Ebrahimi, T.; Pun, T.; Nijholt, A.; Patras, I. DEAP: A Database for Emotion Analysis Using Physiological Signals. *IEEE Transactions on Affective Computing* **2012**, *3*, 18–31. https://doi.org/10.1109/T-AFFC.2011.15.
50. Katsigiannis, S.; Ramzan, N. DREAMER: A Database for Emotion Recognition Through EEG and ECG Signals From Wireless Low-cost Off-the-Shelf Devices. *IEEE Journal of Biomedical and Health Informatics* **2018**, *22*, 98–107. https://doi.org/10.1109/JBHI.2017.2688239.

51. Sharma, K.; Castellini, C.; van den Broek, E.L.; Albu-Schaeffer, A.; Schwenker, F. A Dataset of Continuous Affect Annotations and Physiological Signals for Emotion Analysis. *Scientific Data* **2019**, *6*, 196. https://doi.org/10.1038/s41597-019-0209-0.
52. Bustos, C.; Elhaouij, N.; Solé-Ribalta, A.; Borge-Holthoefer, J.; Lapedriza, A.; Picard, R. Predicting Driver Self-Reported Stress by Analyzing the Road Scene. In Proceedings of the 2021 9th International Conference on Affective Computing and Intelligent Interaction (ACII), Sep. 2021, pp. 1–8. https://doi.org/10.1109/ACII52823.2021.9597438.
